# Integrating reductive and synthetic approaches in biology using man-made cell-like compartments

**DOI:** 10.1038/srep04722

**Published:** 2014-04-17

**Authors:** Wataru Aoki, Masato Saito, Ri-ichiroh Manabe, Hirotada Mori, Yoshinori Yamaguchi, Eiichi Tamiya

**Affiliations:** 1 Department of Applied Physics, Graduate School of Engineering, Osaka University, 2-1 Yamadaoka, Suita, Osaka 565-0871, Japan; 2 Japan Society for the Promotion of Science, 2-1 Yamadaoka, Suita, Osaka 565-0871, Japan; 3 Genome Network Analysis Support Facility, Division of Genomics Technologies, RIKEN Center for Life Science Technologies, 1-7-22 Suehiro-cho, Tsurumi-ku, Yokohama, Kanagawa 230-0045, Japan; 4 Graduate School of Biological Sciences, Nara Institute of Science and Technology, 8916-5 Takayama, Ikoma, Nara 630-0101, Japan

## Abstract

We propose ‘integrated synthetic genetics' as a novel methodology that integrates reductive and synthetic approaches used in life science research. Integrated synthetic genetics enables determinations of sets of genes required for the functioning of any biological subsystem. This method utilizes artificial cell-like compartments, including a randomly introduced whole gene library, strictly defined components for *in vitro* transcription and translation and a reporter that fluoresces ‘only when a particular function of a target biological subsystem is active.' The set of genes necessary for the target biological subsystem can be identified by isolating fluorescent artificial cells and multiplex next-generation sequencing of genes included in these cells. The importance of this methodology is that screening for the set of genes involved in a subsystem and reconstructing the entire subsystem can be done simultaneously. This methodology can be applied to any biological subsystem of any species and may remarkably accelerate life science research.

Life science research seeks to elucidate the relationships between genotypes and phenotypes. This typically involves reductive (genetic and omics research) and synthetic (synthetic biology) approaches. Genetic and omics research seeks to identify genes involved in a biological subsystem of interest such as transcription, translation, signal transduction, genome repair and metabolism. This approach enables identifying individual genes in a target biological subsystem, although the entirety of the subsystem is not readily characterized. To overcome this, synthetic approaches are used, in which a known set of genes is isolated and combined to reconstruct a target biological subsystem with the aim of a complete proof for the entirety of the subsystem. Life science research combines these two methodologies. However, it is often difficult to fill in information gaps in a target subsystem on the basis of these approaches, and tremendous amounts of time and effort are required to completely understand a subsystem's functions.

To integrate reductive and synthetic approaches, we propose ‘integrated synthetic genetics', a novel approach that integrates the advantages of reductive and synthetic approaches. This method provides for simultaneous high-throughput implementation of screening for genes involved in a particular subsystem and reconstructing the entire subsystem. The core of this approach is incorporating artificial cell-like compartments, including an *in vitro* transcription and translation system (PURE system)^1^. The elements of a PURE system are strictly determined, and a functional protein can be synthesized by introducing any gene fragment in these artificial compartments^2^. Because as many as 10^8^ artificial cell-like compartments can be constructed and the functions of the genes inside these compartments can be evaluated while maintaining genotype–phenotype associations, they have been used for the directed evolution of proteins and RNAs^3^,^4^,^5^,^6^.

Figure 1 shows an outline of our proposed methodology. First, a whole gene library from a target organism is prepared and artificial cell-like compartments that contain randomly introduced library components are constructed (Fig. 1a). Simultaneously, a reporter that fluoresces ‘only when a particular function of a target biological subsystem is active' is introduced (Fig. 1b). Next, a liposome library that contains various combinations of genes is constructed (Fig. 1c). Because proteins are synthesized using these introduced gene combinations and express their functions within the PURE system, these liposomes will fluoresce if they contain that set of genes required for the target biological subsystem's function (Fig. 1c). These fluorescent liposomes are isolated by FACS (Fig. 1d), and the genes in each of the fluorescent liposomes are determined by multiplex next-generation sequencing^7^ (Fig. 1e). Because each liposome also contains many irrelevant genes, those genes that are common in fluorescent liposomes are identified by hierarchical cluster analysis (Fig. 1f). Finally, these common factors are considered to be a set of genes that are required for a target biological subsystem. The importance of this methodology is that screening for genes involved in a subsystem and reconstructing the whole subsystem can be done simultaneously on the basis of the artificial cell-like compartments that have strictly defined contents and genetic-like screening that begins with a whole gene library. This methodology can be realized due to the large-scale information processing capacity of next-generation sequencing.

## Results and Discussion

### β-Galactoside hydrolysis subsystem

**Figure 2:**
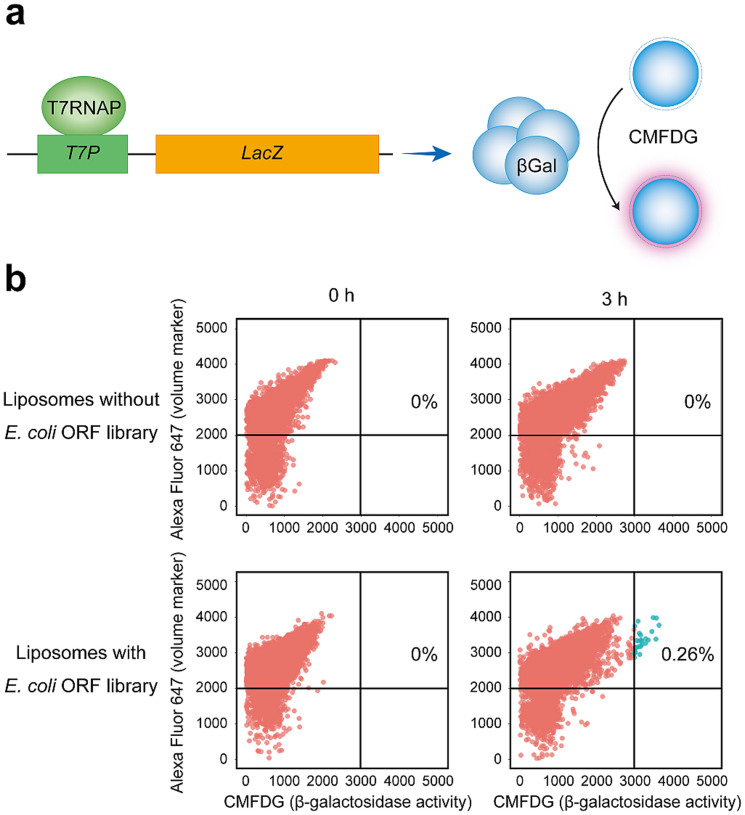
Ultrahigh-throughput reconstruction of the β-Galactoside hydrolysis subsystem. (a) Fluorescent reporter for β-galactoside hydrolysis activity. When *LacZ* that hydrolyzes β-galactosides is expressed in a liposome, CMFDG is hydrolyzed and fluoresces. (b) FACS analysis of the liposome library. When liposomes containing the PURE system solution, CMFDG, a transferrin-Alexa Fluor 647 conjugate and 5 nM *E. coli* ORF library were constructed, liposomes that emitted CMFDG-derived fluorescence (upper right quadrant) occurred at a rate of 0.26%. No fluorescence was observed for liposomes without the *E. coli* ORF library.

To demonstrate the feasibility of our methodology, we selected the *Escherichia coli* ‘β-galactoside hydrolysis subsystem' as our target. In *E. coli*, β-galactosidase encoded for by *LacZ* is necessary and sufficient for β-galactoside hydrolysis^8^. Thus, this target represents the simplest of model systems. To detect this subsystem, we used 5-chloromethylfluorescein di-β-D-galactopyranoside (CMFDG) as the reporter. CMFDG is a non-fluorescent molecule that contains two galactose moieties and fluoresces when these galactose moieties are hydrolysed (Fig. 2a). Using a bulk assay, we verified β-galactoside hydrolysis activity in the PURE system solution. We mixed *LacZ* with a T7 promoter (T7P-LacZ), a PURE system solution and CMFDG and then incubated this mixture at 37°C. This resulted in intense fluorescence derived from CMFDG, which indicated a reliable level of activity with this PURE system (Supplementary Fig. 1).

### Construction of the *E. coli* ORF library

We constructed an *E. coli* ORF library comprising 4,123 genes with a T7 promoter, on the basis of the ASKA library that contains 4,132 *E. coli* strains harbouring an *E. coli* gene plasmid library. To simplify the construction of the *E. coli* ORF library, 4,132 *E. coli* strains were divided into groups with approximately 100 strains per group. Each group was cultured in Luria–Bertani medium, and plasmids from each group were purified. Gene fragments with a T7 promoter were amplified using PCR. Finally, equal amounts of the amplified gene fragments were mixed to prepare the *E. coli* ORF library. We used deep sequencing to check the quality of this library. Almost all genes (96.7%) were sequenced at least once, which assured the library's quality (Supplementary Fig. 2 and Supplementary Table 1).

### IVTT reaction in liposomes

We attempted ultrahigh-throughput reconstruction of the ‘β-galactoside hydrolysis subsystem' by starting with the *E. coli* ORF library. First, we prepared a liposome library that contained the PURE system solution, 100 μM CMFDG, 1 μM transferrin-Alexa Fluor 647 conjugate (volume marker) and 5 nM *E. coli* ORF library. Microscopic inspection indicated that the average size and volume of these liposomes were 2.4 μm and 7.2 fL, respectively (Supplementary Fig. 3), which indicated that approximately 20 genes were randomly incorporated in each liposome. Using the formula for combination with repetitions, the probability of having a given target gene among 20 genes randomly chosen from 4,123 genes was 0.48%. Subsequently, these liposomes were incubated at 37°C to allow for gene expression and analysed by FACS. By FACS analysis, particles that emitted Alexa Fluor 647-derived fluorescence were classified as liposomes. This showed that CMFDG-derived fluorescence was not detected in liposomes devoid of the *E. coli* ORF library, whereas 0.26% of liposomes that contained the *E. coli* ORF library fluoresced (Fig. 2b). This indicated that fluorescent signals were derived from functioning genes incorporated in these liposomes. The theoretical and experimental proportions of fluorescent liposomes (0.48% and 0.26%, respectively) were similar. These results indicated that genes were distributed in a random manner and that once distributed, the genes in these liposomes were correctly translated into functional proteins.

### Multiplex next-generation sequencing

**Figure 3:**
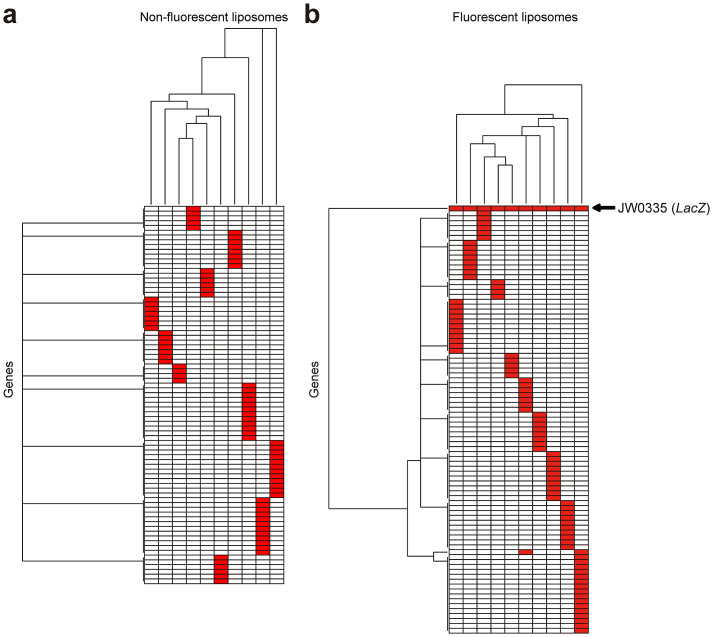
(a) Hierarchical cluster analysis for genes included in non-fluorescent liposomes. Ten non-fluorescent liposomes that did not show β-galactoside hydrolysis activity were isolated, and the genes included in these liposomes were determined by multiplex next-generation sequencing using HiSeq 2500. In the hierarchical cluster analysis for these genes, there was no commonality among the genes included in each liposome, which indicated a random pattern. Each row represents a separate gene, each column represents a separate liposome and genes found in each liposome are shown in red. (b) Hierarchical cluster analysis for the genes included in fluorescent liposomes. Hierarchical cluster analysis revealed that *LacZ* was a clear cluster that was included in all fluorescent liposomes and with no other common factors.

**Figure 4:**
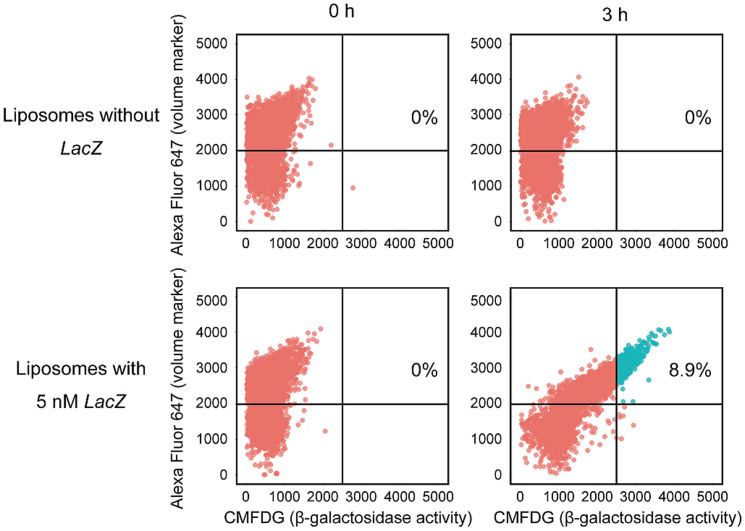
β-Galactoside hydrolysis activity in artificial cell-like compartments. Multiplex sequencing identified *LacZ* as a common gene that was included in fluorescent liposomes. To verify this, liposomes that contained the PURE system solution, 100 μM CMFDG, 1 μM transferrin-Alexa Fluor 647 conjugate (volume marker) and 5 nM *LacZ* were constructed and assayed for their reproducibility of β-galactoside hydrolysis activity. As a negative control, liposomes without *LacZ* were also constructed. Liposomes were incubated at 37°C and analysed by FACS. At 0 h, CMFDG-derived fluorescence (abscissa) was not detected in either group of liposomes, while after 3 h of incubation, intense fluorescence was detected only in liposomes that contained 5 nM *LacZ*, which verified the reconstruction of β-galactoside hydrolysis activity. Almost all liposomes had shifted to the right and 8.9% of these liposomes were in the upper right quadrant, which indicated intense fluorescence.

To identify the genes required for the β-galactoside hydrolysis subsystem, fluorescent liposomes and control non-fluorescent liposomes were isolated, and the genes included in them were determined by multiplex next-generation sequencing (Supplementary Table 2). Each liposome contained numerous genes irrelevant to the β-galactoside hydrolysis subsystem. Thus, we used hierarchical cluster analysis for the genes in the isolated liposomes to detect any specific patterns and to identify common genes. Hierarchical cluster analysis revealed no common factor(s) in our control analysis of non-fluorescent liposomes (Fig. 3a). In contrast, *LacZ* was a clear cluster that was included in all fluorescent liposomes and with no other common factors (Fig. 3b). This indicated successful ultrahigh-throughput reconstruction of the β-galactoside hydrolysis subsystem from the *E. coli* ORF library by integrated synthetic genetics. Furthermore, the conclusions drawn from our cluster analysis were confirmed by additional data that virtually all liposomes constructed using *LacZ* only were fluorescence positive (Fig. 4). Although we used the β-galactoside hydrolysis subsystem as a target in this study, similar ultrahigh-throughput reconstructions can be performed for any biological subsystem using an appropriate reporter. Integrated synthetic genetics can be applied to more complex systems such as cancer. One of the fundamental characteristics of cancer cells is unlimited cell proliferation which involves promoting cell survival and blocking apoptosis^19^. Consistently, it is known that a few key hallmarks related to apoptosis, cytoskeleton and genomic instability are significantly enriched in tumor genomic alterations^19^,^20^. Reconstruction and modelling of cancer hallmarks-specific networks will provide insights into cancer therapies.

In conclusion, in this study, we successfully constructed integrated synthetic genetics as a novel method to integrate reductive and synthetic approaches. This system combines the advantages of reductive and synthetic approaches and has three beneficial features (Supplementary Fig. 4). First, this method provides for simultaneous high-throughput implementation of screening and reconstruction, which have been previously used as fundamentally distinct methods in biological research. Second, if an appropriate reporter is available, this method provides for ultrahigh-throughput reconstruction of any biological subsystem of any species. Using a cDNA library, this system may even be applicable to non-model organisms for which a whole gene library is unavailable. Third, even when a target subsystem involves numerous unknown factors, screening for genes that are necessary and sufficient is feasible using multiplex sequencing. For example, to address a subsystem that involves five unknown factors within the context of all *E. coli* genes, conventional methods would require 4123^5^ ≈ 10^18^ combinations of experiments, which are practically impossible to implement. With our approach, assuming that 200 genes are randomly introduced into one liposome, the probability that this liposome contains all five unknown genes would be 10^−7^, which would correspond to a 10^11^-fold increase in efficiency compared with a conventional approach and therefore, detection becomes sufficiently realistic.

As demonstrated in this study using the β-galactoside hydrolysis subsystem, our method offers the advantage of faster identification, even for single gene identification, as compared with conventional methods. In practical terms, our method takes approximately one week to complete liposome construction, reactions, isolation, multiplex sequencing and data analysis. Thus, our proposed method provides a novel method for life science research and has the potential to substantially enhance research efficiency.

## Methods

### *E. coli* ORF library

The ASKA library (GFP non-fusion type)^9^ contained all 4,132 genes of *E. coli* and was provided by the NBRP National Institute of Genetics (Mishima, Japan). All 4,132 genes were amplified by PCR using ASKA library plasmids as previously described^10^, with some modifications. In brief, 4,132 *E. coli* strains obtained from the NBRP were divided into groups with approximately 100 strains per group. Each group was cultured in 100 mL of Luria–Bertani medium + chloramphenicol (0.5% *w/v* yeast extract, 1% *w/v* tryptone, 1% *w/v* NaCl and 20 μg/mL of chloramphenicol). Next, plasmids were extracted from each group and gene fragments were amplified using the following common primers: ASKA forward primer, 5′-GGCC*TAATACGACTCACTATAGG*AGAAATCATAAAAAATTTATTTGCTTTGTGAGCGG-3′, and ASKA reverse primer, 5′-GTTATTGCTCAGCGG*TTA*GCGGCCGCATAGGCC-3′. ASKA forward primers contained the T7 promoter sequence (*Italicized*) for gene expression by the PURE system, and ASKA reverse primers contained the stop codon (*Italicized*). The amplified gene fragments in each group were purified, and equal amounts were mixed to prepare the *E. coli* ORF library with the added T7 promoter. The average length of all *E. coli* genes was 880 bp; this value was used to estimate the molarity of the *E. coli* ORF library.

### *In vitro* transcription and translation (IVTT) system

The IVTT system, PURE system, used in this study was purchased from GeneFrontier Corporation (Chiba, Japan). The composition of the PURE system was previously described^1^,^11^. In brief, the PURE system contained purified ribosomes, translation initiation factors, elongation factors, release factors, aminoacyl-tRNA synthetases, methionyl-tRNA transformylase, T7 RNA polymerase and a DnaK chaperone. In addition, this system contained tRNAs, NTPs, creatine phosphate, 10-formyl-5,6,7,8-tetrahydrofolic acid, 20 amino acids, creatine kinase, myokinase, nucleoside-diphosphate kinase and phyrophosphatase.

### IVTT reaction under bulk conditions

The IVTT reaction solution was prepared by mixing the PURE system solution, DNA fragments amplified from the ASKA library and 100 μM 5-chloromethylfluorescein di-β-d-galactopyranoside (CMFDG; Life Technologies, Carlsbad, CA, USA). CMFDG has two galactose moieties and is one of the most sensitive substrates for galactosidases. Hydrolysis of non-fluorescent CMFDG can be monitored by an increase in its fluorescence. The reaction solution was incubated at 37°C and fluorescent signals were monitored every 10 min at λ_ex_ = 490 ± 10 nm and λ_em_ = 516 ± 10 nm using an Infinite M1000 fluorescence microplate reader (TECAN, Männedorf, Switzerland).

### IVTT reaction in liposomes

Liposomes were constructed by the water-in-oil emulsion-transfer method as previously described^12^,^13^,^14^, with some modifications. In brief, 1 mL of liquid paraffin containing 250 μg of 1-palmitoyl-2-oleoyl-sn-glycero-3-phosphocholine (Avanti Polar Lipids, Alabaster, AL, USA) and 25 μg of cholesterol (Nakalai Tesque, Kyoto, Japan) were mixed with the IVTT reaction solution using a syringe pump to prepare water-in-oil emulsion droplets^3^. The IVTT reaction solution contained 20 μL of the PURE system solution supplemented with 5 nM *E. coli* ORF library, 200 mM sucrose, 0.5 U/uL of RNase inhibitor (RNasin plus, Promega, Madison, WI, USA), 100 μM CMFDG and 1 μM transferrin-Alexa Fluor 647 conjugate (Life Technologies) as a volume marker. The water-in-oil emulsion droplets were mixed with a magnetic stirrer for 1 min and then equilibrated on ice for 10 min to stabilize the emulsions. The mixture was gently placed in 150 μL of the PURE system solution that contained 200 mM glucose in a microtube and was centrifuged at 15,000 × *g* for 30 min. Prepared liposomes were removed through an opening at the bottom of the tube. It is important that the liposomes are dispersed in the PURE system solution to prolong protein production in these liposomes^15^. The prepared liposomes were incubated at 37°C for protein production.

### FACS analysis

A JSAN cell sorter (Bay Bioscience, Hyogo, Japan) and a FACSAria (Becton Dickinson, Franklin Lakes, NJ, USA) were used for liposome sorting and analysis. CMFDG-derived fluorescence and Alexa Fluor 647-derived fluorescence were monitored separately using a dual band pass filter. Among the particles detected by FACS analysis, those that emitted Alexa Fluor 647-derived fluorescence were classified as liposomes. CMFDG-derived fluorescent liposomes were classified as liposomes that contained the genes required for the β-galactoside hydrolysis subsystem and sorted accordingly. As a negative control, non-fluorescent liposomes that did not emit CMFDG-derived fluorescence were similarly isolated.

### Illumina sequencing

To evaluate the quality of the *E. coli* ORF library and determine the genes included in the isolated liposomes, multiplex next-generation sequencing was done using HiSeq 2500 (Illumina, San Diego, CA, USA). First, gene fragments in the isolated liposomes were amplified by 45 PCR cycles using the primers noted above (i.e. ASKA forward and reverse primers) and KOD-Plus- (Toyobo, Osaka, Japan). The concentrations of amplified gene fragments were determined using Quant-iT PicoGreen (Life Technologies). Next, the liposome-derived DNA fragments and the *E. coli* ORF library were pre-treated for HiSeq 2500 according to the Nextera XT DNA preparation kit protocol (Illumina). In brief, input DNA was fragmented by a transposome and the dual indexes were tagged by limited-cycle PCR, which allowed for discriminating between DNA fragments derived from different samples. Equal amounts of DNA fragments tagged with dual indexes were mixed for 50-bp single-read sequencing on HiSeq 2500 in rapid-run mode, and approximately 68 million mapped reads were obtained. Next-generation sequencing was done by the Genome Network Analysis Support Facility of Riken.

### Data analysis

Mapping of the output data from HiSeq 2500 was done for *E. coli* ORF nucleotide sequences obtained from Genobase (http://ecoli.naist.jp/GB8-dev/index.jsp?page = genome_download.jsp) using Bowtie2^16^. The abundance of ORFeome clones was quantified using R software. To verify the quality of the *E. coli* ORF library, the number of reads for each of the 4,123 genes was counted using sequence data (Supplementary Table 1). To identify the necessary and sufficient conditions for the β-galactoside hydrolysis subsystem, the genes in the isolated liposomes were determined. To remove non-specific mapping, identified genes were listed using a threshold of 10,000 reads (Supplementary Table 2). Hierarchical cluster analysis using Cluster 3.0^17^ was used to detect any specific patterns among the genes in liposomes. To organize clusters, complete linkage was used as the clustering method and Euclidean distances were used as similarity measures. JAVA Treeview^18^ was used to visualize the clustering results.

## Author Contributions

W.A. conceived the project and M.S., Y.Y. and E.T. supervised the project. W.A. performed experiments and data analysis. R.M. provided advice on method development with respect to next generation sequencing. H.M. contributed to constructing the *E. coli* ORF library. The manuscript was prepared by W.A. and edited by M.S., R.M., H.M., Y.Y. and E.T.

## Supplementary Material

Supplementary Information Supplementary Information

## Figures and Tables

**Figure 1:**
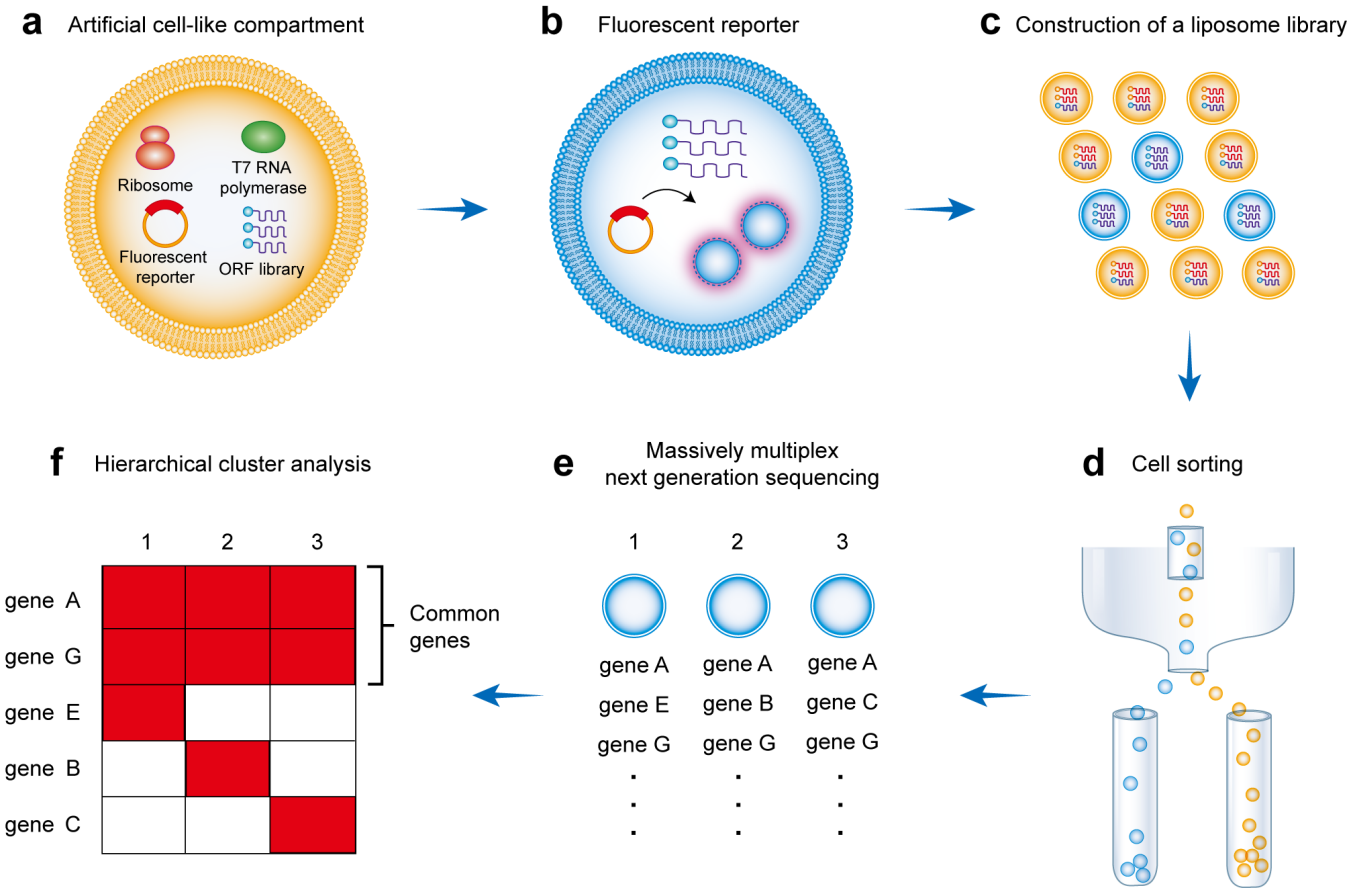
Strategy used for integrated synthetic genetics. (a) Construction of an artificial cell-like compartment. The artificial cell contains ribosomes, RNA polymerases, fluorescent reporters and a randomly distributed ORF library. (b) Fluorescent reporter. The reporter fluoresces only when the target biological subsystem's function is active. (c) Construction of a liposome library. Reconstructing the function of the target subsystem occurs in a subpopulation of the liposome library (shown in blue) that contains that set of genes required for the target biological subsystem, resulting in fluorescence. (d) Sorting fluorescent liposomes by FACS. (e) Identifying incorporated genes by multiplex next-generation sequencing. (f) Identifying common factors by hierarchical cluster analysis.

## References

[b1] Shimizu Y. *et al.* Cell-free translation reconstituted with purified components. Nat Biotechnol 19, 751–755 (2001). 11479568 10.1038/90802

[b2] Yu W. *et al.* Synthesis of functional protein in liposome. J Biosci Bioeng 92, 590–593 (2001). 16233152 10.1263/jbb.92.590

[b3] Tawfik D. S. & Griffiths A. D. Man-made cell-like compartments for molecular evolution. Nat Biotechnol 16, 652–656 (1998). 9661199 10.1038/nbt0798-652

[b4] Sunami T. *et al.* Femtoliter compartment in liposomes for *in vitro* selection of proteins. Anal Biochem 357, 128–136 (2006). 16889743 10.1016/j.ab.2006.06.040

[b5] Fujii S., Matsuura T., Sunami T., Kazuta Y. & Yomo T. *In vitro* evolution of alpha-hemolysin using a liposome display. Proc Natl Acad Sci U S A 110, 16796–16801 (2013). 24082135 10.1073/pnas.1314585110 PMC3801045

[b6] Ichihashi N. *et al.* Darwinian evolution in a translation-coupled RNA replication system within a cell-like compartment. Nature commun 4, 2494 (2013). 24088711 10.1038/ncomms3494

[b7] Meyer M., Stenzel U., Myles S., Prufer K. & Hofreiter M. Targeted high-throughput sequencing of tagged nucleic acid samples. Nucleic Acids Res 35, e97 (2007). 17670798 10.1093/nar/gkm566 PMC1976447

[b8] Nolan G. P., Fiering S., Nicolas J. F. & Herzenberg L. A. Fluorescence-activated cell analysis and sorting of viable mammalian cells based on beta-d-galactosidase activity after transduction of *Escherichia coli lacZ*. Proc Natl Acad Sci U S A 85, 2603–2607 (1988). 3128790 10.1073/pnas.85.8.2603 PMC280046

[b9] Kitagawa M. *et al.* Complete set of ORF clones of *Escherichia coli* ASKA library (a complete set of *E. coli* K-12 ORF archive): unique resources for biological research. DNA Res 12, 291–299 (2005). 16769691 10.1093/dnares/dsi012

[b10] Niwa T. *et al.* Bimodal protein solubility distribution revealed by an aggregation analysis of the entire ensemble of *Escherichia coli* proteins. Proc Natl Acad Sci U S A 106, 4201–4206 (2009). 19251648 10.1073/pnas.0811922106 PMC2657415

[b11] Shimizu Y., Kanamori T. & Ueda T. Protein synthesis by pure translation systems. Methods 36, 299–304 (2005). 16076456 10.1016/j.ymeth.2005.04.006

[b12] Pautot S., Frisken B. J. & Weitz D. A. Production of unilamellar vesicles using an inverted emulsion. Langmuir 19, 2870–2879 (2003).

[b13] Nishimura K. *et al.* Population analysis of structural properties of giant liposomes by flow cytometry. Langmuir 25, 10439–10443 (2009). 19670878 10.1021/la902237y

[b14] Nishikawa T., Sunami T., Matsuura T., Ichihashi N. & Yomo T. Construction of a gene screening system using giant unilamellar liposomes and a fluorescence-activated cell sorter. Anal Chem 84, 5017–5024 (2012). 22519524 10.1021/ac300678w

[b15] Noireaux V. & Libchaber A. A vesicle bioreactor as a step toward an artificial cell assembly. Proc Natl Acad Sci U S A 101, 17669–17674 (2004). 15591347 10.1073/pnas.0408236101 PMC539773

[b16] Langmead B. & Salzberg S. L. Fast gapped-read alignment with Bowtie 2. Nat Methods 9, 357–359 (2012). 22388286 10.1038/nmeth.1923 PMC3322381

[b17] de Hoon M. J., Imoto S., Nolan J. & Miyano S. Open source clustering software. Bioinformatics 20, 1453–1454 (2004). 14871861 10.1093/bioinformatics/bth078

[b18] Saldanha A. J. Java Treeview--extensible visualization of microarray data. Bioinformatics 20, 3246–3248 (2004). 15180930 10.1093/bioinformatics/bth349

[b19] Wang E. *et al.* Cancer systems biology in the genome sequencing era: Part 1, dissecting and modeling of tumor clones and their networks. Sem Cancer Biol 23, 279–285 (2013). 10.1016/j.semcancer.2013.06.002 23791722

[b20] Wang E. *et al.* Cancer systems biology in the genome sequencing era: Part 2, evolutionary dynamics of tumor clonal networks and drug resistance. Sem Cancer Biol 23, 286–292 (2013). 10.1016/j.semcancer.2013.06.001 23792107

