# Supplementary material for: Integrating reductive and synthetic approaches in biology using man-made cell-like compartments
Source: Sci Rep. 2014 Apr 17;4:4722. doi: 10.1038/srep04722 (PMC3989557; doi:10.1038/srep04722)
Supplement: Supplementary Information [file srep04722-s1.pdf]

Scientific Reports

SUPPLEMENTARY INFORMATION

TITLE

Integrating reductive and synthetic approaches in biology using man-made cell-like compartments

AUTHORS AND AFFILIATIONS

Wataru Aoki*, Masato Saito, Ri-ichiroh Manabe, Hirotada Mori, Yoshinori Yamaguchi, Eiichi Tamiya*

Supplementary Figures

Supplementary Figure 1. Verification of β-galactoside hydrolysis activity by bulk assay.

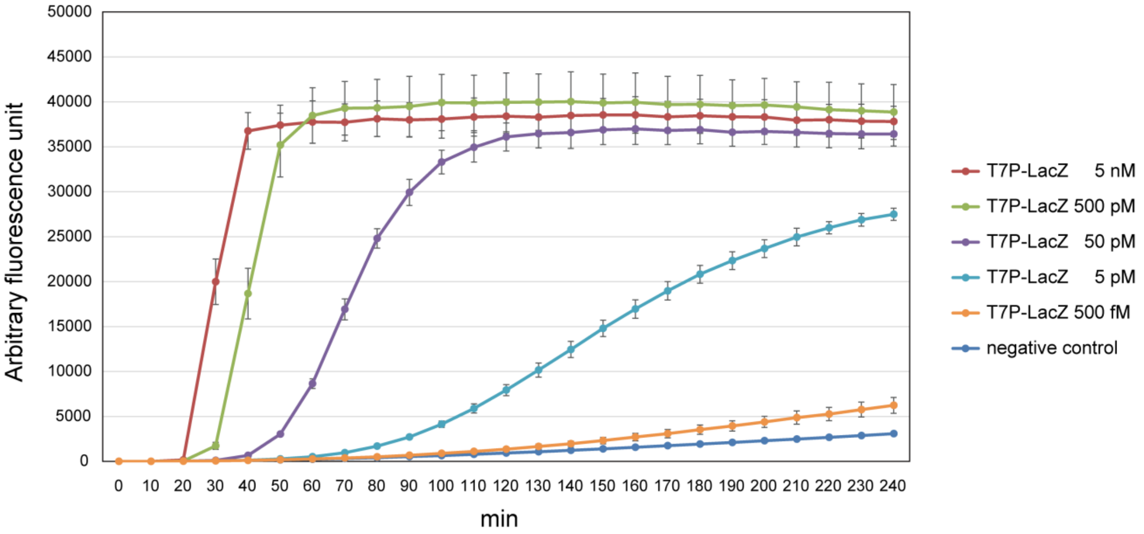

LacZ with a T7 promoter (T7P-LacZ) was mixed with a PURE system solution and CMFDG, and then incubated in 96-well plates at 37°C. Fluorescent signals were measured at 10-min intervals. Higher fluorescence indicated stronger β-galactoside hydrolysis activity. These results indicated that 5 pM T7P-LacZ was sufficient to provide a reliable level of activity. Results are means ± S.D.'s of three independent experiments.

Supplementary Figure 2. Sequencing reads of the *E. coli* ORF library.

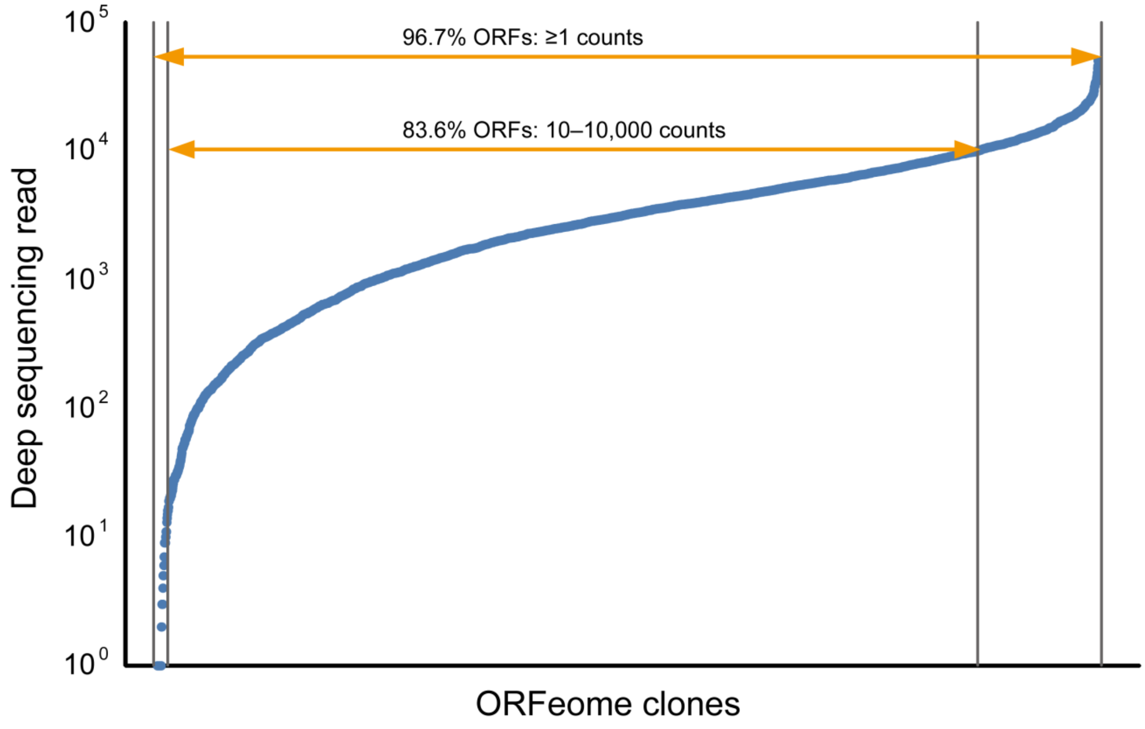

The *E. coli* ORF library (input library) was analyzed using HiSeq 2500 and the majority of ORFeome clones (complete set of ORFs in the *E. coli* genome) were quantified. Almost all ORFeome clones (96.7%) were sequenced at least once. Details for the number of reads are shown in Supplementary Table 1.

Supplementary Figure 3. Construction of artificial cell-like compartments.

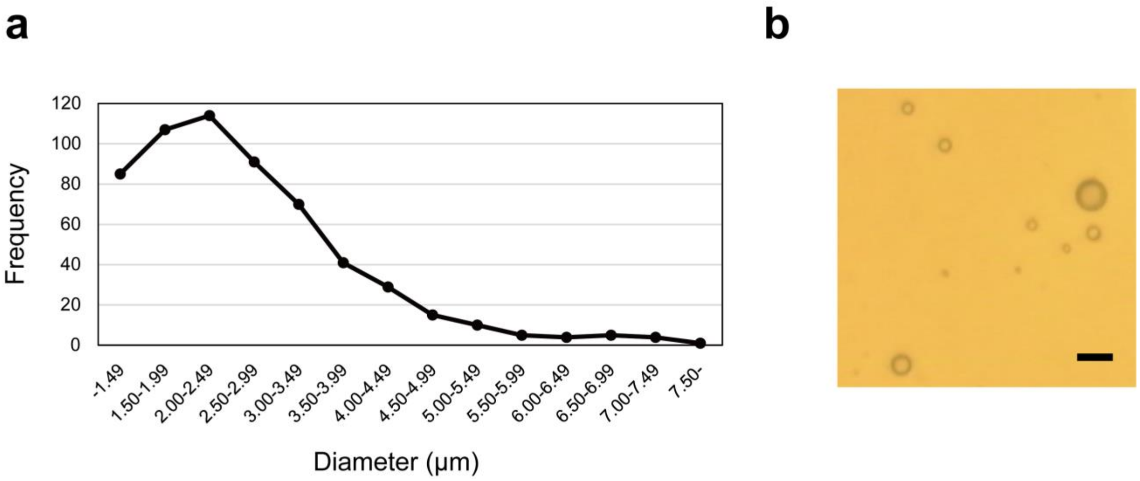

(a) Size distribution of liposomes. Constructed artificial cell-like compartments (liposomes) were observed under a microscope and images were acquired at random points. Based on a size distribution analysis of 855 photographed liposomes, the median diameter was determined to be 2.4 μm and the median volume was determined to be 7.2 fL. (b) Representative image of liposomes. Scale bar = 10 μm.

Supplementary Figure 4. Advantages of integrated synthetic genetics.

a

Methodologies	Random screening	Rational screening	Proof probablity	Throughput
Forward genetics/omics	✓	×	×	Middle/high
Reverse genetics	×	✓	×	Low
Synthetic biology	×	✓	✓	Low
“Integrated synthetic genetics”	✓	✓	✓	High

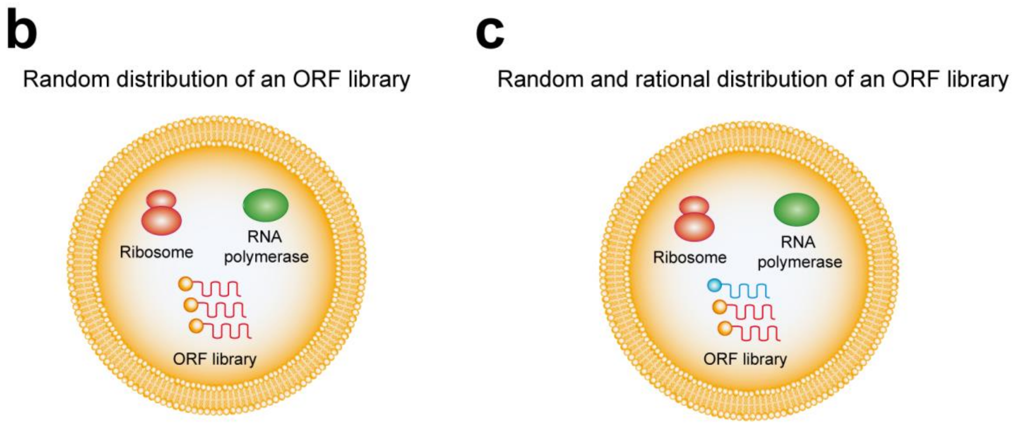

(a) Our proposed integrated synthetic genetics has all the advantages of the primary methodologies used in modern biological sciences, including forward genetics/omics, reverse genetics, and synthesis. Artificial cell-like compartments with strictly defined contents provides for determining the necessary and sufficient conditions for a target biological subsystem, which is an advantage of a synthetic approach. This system employs FACS and multiplex next-generation sequencing, which incorporates remarkably high throughputs. (b) Random distribution of an ORF library provides for screening of the genotype required for a target phenotype, as in forward genetics. (c) Introducing a specific gene (shown in blue) in addition to a randomly distributed ORF library provides for rational exploration of the specific gene’s effects on a target phenotype, as in reverse genetics.

Supplementary Tables

Supplementary Table 1. Sequencing reads for the *E. coli* ORF library. The *E. coli* ORF library was analyzed using HiSeq 2500 and the number of reads for each gene was counted. The number of reads was normalized as reads per kilobase of DNA per million mapped reads (RPKM).

Supplementary Table 2. Genes included in isolated fluorescent and non-fluorescent liposomes. Because of the large amplification bias during PCR amplification of the genes in isolated liposomes, quantitative results cannot be provided. Thus, the result for each gene is shown qualitatively as either detected or not detected.

Supplementary Table 1
Deep sequencing reads for the *E. coli* ORF library

ORF No.	ORF name	Gene length (bp)	Read counts	Reads per kilobase of cDNA per million mapped reads (RPKM)
JW0001	thrA	2,463	4,674	380
JW0002	thrB	933	3,345	716
JW0003	thrC	1,287	12,255	1,901
JW0004	yaaX	297	2,278	1,529
JW0005	yaaA	777	4,798	1,234
JW0006	yaaJ	1,431	8,716	1,219
JW0007	talB	954	1,575	329
JW0008	mog	588	4,131	1,405
JW0009	yaaH	567	5,453	1,915
JW0010	yaaW	714	2,641	740
JW0012	yaaI	405	1,349	666
JW0013	dnaK	1,917	1,448	151
JW0014	dnaJ	1,131	1,564	276
JW0015	insL	1,113	0	0
JW0018	nhaA	1,167	2,431	415
JW0019	nhaR	906	3,059	673
JW0020	insB	504	17	7
JW0021	insA	276	6	4
JW0022	rpsT	264	338	257
JW0023	ribF	942	3,604	767
JW0024	ileS	2,817	6,335	451
JW0025	lspA	495	3,856	1,563
JW0026	fkpB	450	3,527	1,563
JW0027	ispH	951	14,499	3,034
JW0028	rihC	915	34,473	7,530
JW0029	dapB	822	1,282	314
JW0030	carA	1,149	2,293	402
JW0031	carB	3,222	1,635	101
JW0033	caiF	396	1,993	1,006
JW0035	caiD	894	6,962	1,557
JW0036	caiC	1,569	1,411	180
JW0037	caiB	1,218	2,415	397
JW0038	caiA	1,143	1,718	300
JW0039	caiT	1,515	200	26
JW0040	fixA	771	10,105	2,627
JW0041	fixB	942	3,699	786
JW0042	fixC	1,287	1,514	236
JW0043	fixX	288	772	535
JW0044	yaaU	1,332	7,716	1,154
JW0045	kefF	531	6,151	2,318
JW0046	kefC	1,863	3,267	351
JW0047	folA	480	4,854	2,024
JW0048	apaH	843	25,396	6,013
JW0049	apaG	378	4,643	2,448
JW0050	ksgA	822	871	215
JW0051	pdxA	990	10,673	2,159
JW0052	surA	1,287	14,970	2,333
JW0053	imp	2,355	6,978	593
JW0054	djlA	816	2,314	567
JW0055	yabP	651	3,363	1,031
JW0056	yabQ	159	49	61
JW0057	rluA	660	3,311	1,001
JW0058	hepA	2,907	2,665	183
JW0059	polB	2,352	6,696	570
JW0060	araD	696	17,888	5,133
JW0061	araA	1,503	18,933	2,519
JW0062	araB	1,701	17,070	2,007

JW0063	araC	879	2,682	612
JW0065	thiQ	699	7,943	2,273
JW0066	thiP	1,611	12,577	1,566
JW0067	tbpA	984	4,993	1,016
JW0068	sgrR	1,656	3,548	429
JW0069	setA	1,179	6,032	1,018
JW0070	leuD	606	4,211	1,380
JW0071	leuC	1,401	4,117	586
JW0073	leuA	1,572	23,740	3,019
JW0074	leuL	87	62	144
JW0075	leuO	945	998	211
JW0076	ilvI	1,725	5,211	602
JW0077	ilvH	492	3,349	1,357
JW0078	fruR	1,005	23	4
JW0079	mraZ	459	2,502	1,089
JW0080	mraW	942	7,807	1,656
JW0081	ftsL	366	1,233	677
JW0082	ftsI	1,767	3,637	412
JW0083	murE	1,488	9,395	1,262
JW0084	murF	1,359	8,215	1,207
JW0085	mraY	1,083	2,638	488
JW0086	murD	1,317	15,124	2,299
JW0087	ftsW	1,245	7,157	1,145
JW0088	murG	1,068	3,191	600
JW0089	murC	1,476	2,948	398
JW0090	ddlB	921	16,935	3,667
JW0091	ftsQ	831	3,464	834
JW0092	ftsA	1,263	0	0
JW0093	ftsZ	1,152	34,137	5,899
JW0094	lpxC	918	5,295	1,158
JW0096	secA	2,706	7,000	518
JW0097	mutT	390	2,920	1,494
JW0099	yacF	744	1,272	341
JW0100	coaE	621	8,928	2,863
JW0101	guaC	1,044	12,790	2,448
JW0102	hofC	1,203	3,631	606
JW0103	hofB	1,386	4,428	638
JW0104	ppdD	441	10,466	4,735
JW0105	nadC	894	3,718	835
JW0106	ampD	552	7,800	2,817
JW0107	ampE	855	7,341	1,716
JW0108	aroP	1,374	21,324	3,097
JW0109	pdhR	765	4,779	1,254
JW0110	aceE	2,664	3,446	259
JW0111	aceF	1,893	5,876	622
JW0112	lpd	1,425	3,906	548
JW0113	yacH	1,854	4,549	491
JW0114	acnB	2,598	5,925	457
JW0115	yacL	363	2,976	1,631
JW0116	speD	795	2,906	734
JW0117	speE	867	2,656	613
JW0118	yacC	348	987	565
JW0119	cueO	1,551	5,612	720
JW0120	gcd	2,391	10,665	893
JW0122	can	663	3,026	913
JW0123	yadG	927	4,364	940
JW0124	yadH	771	15,195	3,939
JW0125	yadI	441	3,322	1,505
JW0126	yadE	1,230	2,840	463
JW0127	panD	381	1,544	812
JW0129	panC	852	617	145

JW0130	panB	795	3,292	831
JW0131	yadC	1,239	824	133
JW0132	yadK	597	993	334
JW0133	yadL	606	843	275
JW0134	yadM	570	1,933	674
JW0135	htrE	2,598	356	27
JW0136	ecpD	741	899	244
JW0137	yadN	585	1,350	459
JW0138	folK	480	2,977	1,240
JW0141	dksA	456	409	179
JW0142	sfsA	705	2,819	803
JW0144	hrpB	2,430	3,102	255
JW0145	mrcB	2,535	5,842	461
JW0146	fhuA	2,244	2,453	220
JW0147	fhuC	798	6,460	1,614
JW0148	fhuD	891	2,128	474
JW0149	fhuB	1,983	4,837	488
JW0150	hemL	1,281	1,879	292
JW0152	yadR	345	1,632	943
JW0153	yadS	624	2,913	933
JW0154	btuF	801	5,177	1,296
JW0155	pfs	699	530	151
JW0156	dgt	1,518	1,577	209
JW0157	degP	1,425	4,355	614
JW0159	yaeH	387	0	0
JW0161	dapD	825	4,529	1,092
JW0162	glnD	2,673	8,790	657
JW0163	map	795	3,304	832
JW0164	rpsB	726	2,968	814
JW0165	tsf	852	5,476	1,282
JW0166	pyrH	726	5,269	1,453
JW0167	frr	558	1,755	627
JW0168	dxr	1,197	812	136
JW0169	ispU	762	3,040	799
JW0171	yaeL	1,353	2,834	418
JW0172	yaeT	2,433	1,794	148
JW0173	hlpA	486	1,354	556
JW0174	lpxD	1,026	8,216	1,596
JW0175	fabZ	456	1,747	768
JW0176	lpxA	789	8,231	2,081
JW0177	lpxB	1,149	3,333	582
JW0178	rnhB	597	11,615	3,886
JW0179	dnaE	3,483	3,576	207
JW0180	accA	960	2,599	542
JW0181	ldcC	2,142	11,765	1,097
JW0182	yaeR	390	1,451	740
JW0183	tilS	1,299	7,142	1,105
JW0184	rof	255	558	437
JW0185	yaeP	201	273	270
JW0186	yaeQ	546	1,109	404
JW0187	yaeJ	423	2,261	1,067
JW0188	nlpE	711	6,580	1,846
JW0190	proS	1,719	4,427	515
JW0191	yaeB	708	7,413	2,090
JW0192	rcsF	405	5,921	2,935
JW0193	metQ	816	4,059	992
JW0194	metI	654	4,959	1,511
JW0195	metN	1,032	18,219	3,527
JW0196	gmhB	576	6,643	2,293
JW0197	dkgB	804	949	235
JW0198	yafC	915	1,904	416

JW0200	yafE	624	8,056	2,578
JW0202	gloB	756	923	243
JW0203	yafS	723	2,519	698
JW0204	rnhA	468	509	220
JW0205	dnaQ	732	2,903	791
JW0206	yafT	786	381	98
JW0207	yafU	339	478	279
JW0208	yafF	189	247	255
JW0210	ivy	474	3,974	1,667
JW0212	lpcA	579	8,697	2,990
JW0213	yafJ	768	4,204	1,092
JW0214	yafK	741	559	151
JW0215	yafQ	279	320	226
JW0216	dinJ	261	2,043	1,560
JW0217	yafL	750	3,913	1,047
JW0218	yafM	498	460	182
JW0221	dinB	1,056	9,143	1,729
JW0222	yafN	294	257	175
JW0223	yafO	399	466	228
JW0224	yafP	453	630	280
JW0225	ykfJ	267	830	619
JW0226	prfH	501	1,699	675
JW0227	pepD	1,458	17,268	2,364
JW0228	gpt	459	2,786	1,206
JW0229	frsA	1,245	6,052	968
JW0230	crl	402	1,916	946
JW0231	phoE	1,056	1,654	315
JW0232	proB	1,104	6,513	1,179
JW0233	proA	1,254	1,900	303
JW0234	ykfI	342	1,010	590
JW0235	yafW	318	2,527	1,590
JW0236	ykfG	477	885	368
JW0239	ykfB	468	4,400	1,876
JW0240	yafY	858	4,308	1,000
JW0242	yafZ	822	31,844	7,728
JW0243	ykfA	864	33,880	7,799
JW0244	perR	894	4,530	1,009
JW0246	insI	1,152	15	2
JW0247	insO	426	6,152	2,874
JW0250	insH	1,017	0	0
JW0253	mmuM	933	4,807	1,023
JW0254	afuC	1,047	20,169	3,843
JW0255	afuB	363	1,237	679
JW0256	insB	504	13	5
JW0257	insA	276	5	4
JW0258	ykgN	339	214	126
JW0259	yagB	351	919	522
JW0260	yagA	1,155	11,635	2,025
JW0261	yagE	930	3,893	833
JW0262	yagF	1,968	0	0
JW0263	yagG	1,383	9,125	1,319
JW0264	yagH	1,611	1	0
JW0265	yagI	759	3,114	826
JW0266	argF	1,005	5,307	1,052
JW0268	insB	504	9	4
JW0269	insA	276	3	2
JW0270	yagJ	732	952	262
JW0271	yagK	627	21	7
JW0272	yagL	699	461	133
JW0273	yagM	855	1,488	349
JW0274	yagN	441	2,113	951

JW0275	intF	1,401	257	36
JW0276	yagP	411	3,825	1,849
JW0277	yagQ	957	12,053	2,517
JW0278	yagR	2,199	8,728	794
JW0279	yagS	957	40,344	8,407
JW0280	yagT	690	22,909	6,597
JW0281	yagU	615	11,613	3,762
JW0282	ykgJ	330	2,484	1,501
JW0284	yagW	1,644	12,230	1,495
JW0285	yagX	2,526	3,545	280
JW0286	yagY	669	9,085	2,709
JW0287	yagZ	588	9,124	3,112
JW0291	eaeH	888	7,725	1,740
JW0293	insF	867	140	32
JW0298	ykgD	855	13,894	3,249
JW0300	ykgF	1,428	8,761	1,231
JW0302	ykgH	669	688	204
JW0303	betA	1,671	9,937	1,188
JW0304	betB	1,473	12,026	1,634
JW0305	betI	588	6,968	2,371
JW0306	betT	2,034	6,128	601
JW0307	yahA	1,089	2,640	483
JW0308	yahB	933	15,439	3,306
JW0309	yahC	498	2,397	968
JW0310	yahD	606	4,139	1,355
JW0311	yahE	864	8,013	1,853
JW0312	yahF	1,548	9,585	1,241
JW0313	yahG	1,419	3,003	424
JW0315	yahI	951	8,068	1,695
JW0316	yahJ	1,383	18,999	2,754
JW0317	yahK	1,050	12,541	2,388
JW0318	yahL	816	1,850	456
JW0320	yahN	672	8,444	2,514
JW0321	yahO	276	204	144
JW0322	prpR	1,587	9,090	1,150
JW0323	prpB	891	16,370	3,668
JW0324	prpC	1,170	5,758	980
JW0325	prpD	1,452	23,434	3,222
JW0326	prpE	1,887	16,821	1,782
JW0327	codB	1,260	13,461	2,129
JW0328	codA	1,284	20,972	3,264
JW0330	cynT	660	12,600	3,813
JW0331	cynS	471	5,547	2,356
JW0332	cynX	1,155	17,216	2,966
JW0333	lacA	612	327	106
JW0334	lacY	1,254	35	6
JW0335	lacZ	3,075	17,009	1,104
JW0336	lacI	1,083	5,368	983
JW0337	mhpR	948	4,861	1,019
JW0338	mhpA	1,665	24,210	2,912
JW0339	mhpB	945	1,591	335
JW0340	mhpC	882	20,136	4,559
JW0341	mhpD	810	4,646	1,145
JW0342	mhpF	951	15,843	3,329
JW0343	mhpE	1,014	6,169	1,219
JW0345	yaiL	540	639	238
JW0346	frmB	834	12,915	3,097
JW0347	frmA	1,110	3,329	599
JW0348	frmR	276	4,043	2,936
JW0349	yaiO	774	7,149	1,848
JW0350	yaiX	216	101	92

JW0351	insC	411	0	0
JW0352	insD	906	30	7
JW0355	yaiP	1,197	7,039	1,175
JW0356	yaiS	558	290	103
JW0357	tauA	963	0	0
JW0358	tauB	768	3,230	840
JW0359	tauC	828	3,537	852
JW0360	tauD	852	2,277	536
JW0361	hemB	975	4,223	857
JW0362	yaiT	1,461	1,574	215
JW0363	insF	867	151	35
JW0366	yaiV	669	1,659	495
JW0368	sbmA	1,221	3,096	508
JW0369	yaiW	1,095	7,446	1,362
JW0370	yaiY	309	5,312	3,413
JW0372	ddlA	1,095	6,867	1,257
JW0373	yaiB	261	133	99
JW0374	phoA	1,416	13,667	1,926
JW0376	yaiC	1,116	842	149
JW0377	proC	810	10,900	2,689
JW0378	yaiI	459	7,207	3,140
JW0379	aroL	525	8,668	3,290
JW0380	yaiA	192	368	388
JW0381	aroM	678	3,477	1,028
JW0382	yaiE	285	2,937	2,049
JW0383	ykiA	282	595	420
JW0384	rdgC	912	2,852	628
JW0385	mak	909	9,147	2,012
JW0386	araJ	1,185	12,758	2,150
JW0387	sbcC	3,147	1,263	81
JW0388	sbcD	1,203	22,116	3,671
JW0389	phoB	690	6,707	1,942
JW0390	phoR	1,296	11,039	1,699
JW0391	brnQ	1,320	2,502	378
JW0393	malZ	1,818	22,108	2,432
JW0394	yajB	582	2,512	863
JW0395	queA	1,071	20,457	3,818
JW0396	tgt	1,128	7,160	1,267
JW0397	yajC	333	436	265
JW0398	secD	1,848	1,745	190
JW0399	secF	972	10,666	2,197
JW0400	yajD	348	719	412
JW0401	tsx	885	2,843	643
JW0403	ybaD	450	752	334
JW0404	ribD	1,104	11,198	2,029
JW0405	ribE	471	18,517	7,869
JW0406	nusB	420	5,202	2,475
JW0407	thiL	978	4,645	948
JW0408	pgpA	519	4,694	1,804
JW0409	yajO	975	1,664	340
JW0410	dxs	1,863	5,489	591
JW0411	ispA	900	19,567	4,339
JW0412	xseB	243	3,455	2,828
JW0413	thiI	1,449	12,322	1,707
JW0415	panE	912	0	0
JW0418	cyoE	891	8,042	1,805
JW0419	cyoD	330	1,331	803
JW0420	cyoC	615	2,283	741
JW0421	cyoB	1,992	1,240	124
JW0422	cyoA	948	10,755	2,273
JW0423	ampG	1,476	17,312	2,351

JW0424	yajG	579	4,583	1,586
JW0426	tig	1,299	3,781	582
JW0427	clpP	624	4,757	1,522
JW0428	clpX	1,275	11,389	1,789
JW0429	lon	2,355	1,174	100
JW0430	hupB	273	304	226
JW0431	ppiD	1,872	25	3
JW0432	ybaV	372	1,271	682
JW0433	ybaW	399	1,498	747
JW0434	ybaX	696	9,033	2,601
JW0435	ybaE	1,701	11,127	1,307
JW0436	cof	819	627	151
JW0437	ybaO	459	5,373	2,347
JW0438	mdlA	1,773	14,096	1,592
JW0440	glnK	339	2,201	1,291
JW0441	amtB	1,287	10,207	1,581
JW0442	tesB	861	20,338	4,720
JW0443	ybaY	573	4,122	1,433
JW0444	ybaZ	390	3,843	1,972
JW0445	ybaA	354	454	260
JW0448	maa	552	601	218
JW0449	hha	219	28	24
JW0450	ybaJ	375	433	229
JW0451	acrB	3,150	19,534	1,240
JW0452	acrA	1,194	18,698	3,132
JW0453	acrR	648	736	228
JW0454	kefA	3,363	3	0
JW0455	ybaM	162	268	331
JW0456	priC	528	705	268
JW0457	ybaN	378	1,393	730
JW0458	apt	552	6,441	2,318
JW0459	dnaX	1,932	7,962	823
JW0460	ybaB	330	2,170	1,318
JW0461	recR	606	1,492	492
JW0462	htpG	1,875	4,773	506
JW0463	adk	645	1,016	318
JW0464	hemH	963	7,617	1,576
JW0465	aes	960	1,283	266
JW0466	gsk	1,305	5,040	768
JW0467	ybaL	1,677	6,721	798
JW0468	fsr	1,221	7,802	1,276
JW0469	ushA	1,653	7,597	918
JW0470	ybaK	480	2,336	972
JW0471	ybaP	795	2,913	731
JW0472	ybaQ	342	333	191
JW0473	copA	2,505	2,857	228
JW0474	ybaS	933	2,083	443
JW0475	ybaT	1,293	2,378	367
JW0476	cueR	408	1,158	566
JW0478	ybbK	918	7,249	1,573
JW0479	ybbL	678	1,703	500
JW0482	ybbO	810	2,577	635
JW0483	tesA	627	6,534	2,070
JW0484	ybbA	687	8,836	2,570
JW0485	ybbP	2,415	4,701	389
JW0486	rhsD	4,281	3,179	149
JW0487	ybbC	369	216	119
JW0488	ylbH	711	17,795	4,974
JW0489	ybbD	261	191	149
JW0491	ybbB	1,095	9,891	1,798
JW0492	ybbS	927	4,016	864

JW0493	allA	483	6,115	2,538
JW0494	allR	816	18,653	4,565
JW0495	gcl	1,782	9,764	1,100
JW0496	hyi	777	2,633	673
JW0497	glxR	879	8,388	1,912
JW0498	ybbV	279	227	162
JW0499	ybbW	1,455	4,059	555
JW0500	allB	1,362	4,193	616
JW0501	ybbY	1,302	13,871	2,123
JW0502	glxK	1,146	3,268	570
JW0503	ylbA	786	945	239
JW0504	allC	1,236	7,280	1,182
JW0505	allD	1,050	7,865	1,496
JW0506	fdrA	1,668	3,435	410
JW0507	ylbE	261	1,140	874
JW0508	ylbE	1,002	7,518	1,501
JW0509	ylbF	816	6,122	1,504
JW0510	ybcF	894	17,174	3,827
JW0511	purK	1,068	7,794	1,463
JW0512	purE	510	718	281
JW0513	lpxH	723	585	161
JW0514	ppiB	495	483	194
JW0515	cysS	1,386	2,313	336
JW0516	ybcI	522	1,059	405
JW0518	folD	867	7,281	1,679
JW0519	sfmA	543	1,766	653
JW0520	sfmC	693	321	93
JW0521	sfmD	2,604	7,223	555
JW0525	intD	1,164	4,526	774
JW0526	ybcC	264	785	600
JW0527	ybcD	237	1,886	1,592
JW0529	insF	867	194	44
JW0530	renD	186	295	308
JW0531	emrE	333	527	317
JW0532	ybcK	1,527	737	97
JW0533	ybcL	552	482	175
JW0534	ybcM	798	147	37
JW0535	ybcN	456	3,139	1,373
JW0536	ninE	171	80	89
JW0537	ybcO	291	1,186	818
JW0538	rusA	363	1,405	775
JW0539	ybcQ	384	1,479	771
JW0540	insH	1,017	0	0
JW0543	essD	216	138	126
JW0544	ybcS	498	7,102	2,854
JW0546	borD	294	269	186
JW0548	ybcW	207	62	60
JW0549	nohB	546	2,004	733
JW0551	ybcY	585	598	204
JW0552	ylcE	186	231	242
JW0553	appY	750	430	114
JW0554	ompT	954	592	124
JW0555	envY	762	3,601	942
JW0556	ybcH	891	3,839	858
JW0557	nfrA	2,973	1,817	122
JW0558	nfrB	2,238	782	69
JW0560	cusR	684	6,896	2,015
JW0561	cusC	1,374	1,856	270
JW0562	cusF	333	220	131
JW0563	cusB	1,224	3,528	575
JW0564	cusA	3,144	3,545	226

JW0565	pheP	1,377	12,075	1,752
JW0566	ybdG	1,248	1,707	273
JW0567	nfnB	654	869	264
JW0569	ybdJ	249	533	425
JW0570	ybdK	1,119	1,751	315
JW0572	insL	1,113	1	0
JW0576	fes	1,125	10,151	1,806
JW0577	ybdZ	219	1,606	1,463
JW0578	entF	3,882	3,708	192
JW0579	fepE	1,134	3,247	573
JW0580	fepC	816	6,494	1,595
JW0581	fepG	993	8,757	1,762
JW0582	fepD	1,005	15,196	3,017
JW0583	ybdA	1,251	1,456	232
JW0584	fepB	957	1,906	399
JW0585	entC	1,176	3,262	555
JW0586	entE	1,611	10,841	1,345
JW0587	entB	858	8,382	1,946
JW0588	entA	747	15,720	4,206
JW0589	ybdB	414	1,738	839
JW0590	cstA	2,106	3,207	305
JW0591	ybdD	198	448	456
JW0592	ybdH	1,089	4,768	876
JW0593	ybdL	1,161	1,470	252
JW0594	ybdM	630	3,615	1,148
JW0595	ybdN	1,221	8,226	1,349
JW0596	ybdO	903	387	86
JW0597	dsbG	747	223	60
JW0598	ahpC	564	1,080	383
JW0599	ahpF	1,566	9,837	1,253
JW0600	uspG	429	380	178
JW0601	ybdR	1,239	1,487	241
JW0602	rnk	411	6,884	3,348
JW0603	rna	807	3,806	941
JW0604	citT	1,464	11,934	1,631
JW0605	citG	879	15,908	3,600
JW0606	citX	552	11,949	4,323
JW0608	citE	909	3,983	877
JW0609	citD	297	4,466	3,014
JW0610	citC	1,059	11,191	2,108
JW0611	citA	1,659	24,073	2,902
JW0612	citB	681	5,975	1,745
JW0613	dcuC	477	6,939	2,916
JW0616	dcuC	1,092	37,546	6,848
JW0617	crcA	561	2,209	786
JW0618	cspE	210	31	31
JW0619	crcB	384	677	352
JW0620	ybeH	228	0	0
JW0621	ybeM	564	0	0
JW0622	tatE	204	558	553
JW0623	lipA	966	9,813	2,028
JW0624	ybeF	954	2,657	557
JW0626	ybeD	264	337	259
JW0627	dacA	1,212	1,723	284
JW0628	rlpA	1,089	2,501	458
JW0629	mrdB	1,113	13,373	2,404
JW0630	mrdA	1,902	16,174	1,702
JW0631	ybeA	468	5,949	2,535
JW0633	cobC	612	5,717	1,857
JW0634	nadD	642	3,578	1,113
JW0635	holA	1,032	5,017	970

JW0636	rlpB	582	1,957	670
JW0637	leuS	2,583	1,374	107
JW0638	ybeL	483	2,184	902
JW0640	ybeR	708	1,351	380
JW0641	djlB	1,428	1,064	150
JW0642	ybeT	555	501	182
JW0643	ybeU	708	398	112
JW0644	djlC	1,452	1,381	192
JW0645	hscC	1,671	10,745	1,285
JW0646	rihA	936	10,572	2,255
JW0647	gltL	726	12,336	3,398
JW0648	gltK	675	428	127
JW0649	gltJ	741	8,378	2,252
JW0652	insH	1,017	0	0
JW0654	lnt	1,539	4,565	592
JW0655	ybeX	879	8,813	2,002
JW0656	ybeY	468	1,669	716
JW0657	ybeZ	1,080	15,307	2,820
JW0658	miaB	1,425	6,028	845
JW0659	ubiF	1,176	18,222	3,092
JW0660	asnB	1,665	5,986	718
JW0661	nagD	753	6,842	1,813
JW0662	nagC	1,221	5,107	838
JW0663	nagA	1,149	9,078	1,580
JW0664	nagB	801	739	183
JW0665	nagE	1,947	10,808	1,111
JW0666	glnS	1,665	8,205	988
JW0667	ybfM	1,407	12,783	1,807
JW0668	ybfN	327	656	398
JW0669	fur	447	2,396	1,069
JW0671	fldA	531	945	357
JW0673	ybfF	765	3,236	847
JW0674	seqA	546	2,351	866
JW0675	pgm	1,641	5,574	678
JW0676	ybfP	495	1,214	487
JW0679	potE	1,320	3,298	497
JW0680	speF	2,199	10,959	995
JW0683	kdpD	2,685	1,626	121
JW0684	kdpC	573	3,003	1,051
JW0685	kdpB	2,049	3,816	373
JW0686	kdpA	1,674	2,281	270
JW0687	kdpF	90	24	48
JW0688	ybfA	207	1,334	1,291
JW0689	rhsC	4,194	3,970	188
JW0691	ybfB	327	419	258
JW0692	ybfO	1,434	7,769	1,081
JW0693	ybfC	570	335	117
JW0694	ybfQ	255	82	63
JW0695	ybfL	1,062	532	101
JW0696	ybfD	762	640	169
JW0697	ybgA	510	2,560	1,003
JW0698	phr	1,419	9,130	1,283
JW0699	ybgH	1,482	17,234	2,322
JW0700	ybgI	744	7,292	1,960
JW0701	ybgJ	657	3,246	984
JW0702	ybgK	933	9,181	1,963
JW0703	ybgL	735	9,289	2,532
JW0704	nei	792	2,506	631
JW0707	ybgP	729	7,607	2,092
JW0709	ybgD	567	4,353	1,532
JW0710	gltA	1,284	23,091	3,590

JW0711	sdhC	390	2,936	1,500
JW0712	sdhD	348	1,880	1,088
JW0713	sdhA	1,767	11,090	1,259
JW0714	sdhB	717	1,346	377
JW0715	sucA	2,802	7,458	533
JW0716	sucB	1,218	11,949	1,965
JW0717	sucC	1,167	9,247	1,580
JW0718	sucD	870	10,612	2,437
JW0719	mngR	723	1,476	405
JW0720	mngA	1,977	5,750	582
JW0721	mngB	2,634	8,537	651
JW0722	cydA	1,569	12,881	1,640
JW0723	cydB	1,140	11,746	2,060
JW0724	ybgT	114	58	102
JW0725	ybgE	294	1,499	1,021
JW0726	ybgC	405	1,767	872
JW0727	tolQ	693	2,114	609
JW0728	tolR	429	4,835	2,250
JW0729	tolA	1,266	2,578	408
JW0731	pal	522	2,120	809
JW0732	ybgF	792	5,247	1,325
JW0733	nadA	1,044	3,413	652
JW0734	pnuC	720	2,029	568
JW0735	zitB	942	3,063	649
JW0736	ybgS	381	5,492	2,876
JW0737	aroG	1,053	12,545	2,382
JW0738	gpmA	753	2,923	776
JW0739	galM	1,041	18,880	3,624
JW0740	galK	1,149	14,235	2,480
JW0741	galT	1,047	5,148	985
JW0742	galE	1,017	3,713	731
JW0743	modF	1,473	5,908	801
JW0744	modE	789	8,154	2,066
JW0746	modA	774	9,386	2,424
JW0747	modB	690	2,656	765
JW0748	modC	1,059	229	43
JW0749	ybhA	819	713	174
JW0750	ybhE	996	4,058	812
JW0752	ybhH	1,053	4,516	859
JW0753	ybhI	1,434	10,951	1,524
JW0755	ybhC	1,284	50,144	7,811
JW0756	ybhB	477	4,808	2,022
JW0757	bioA	1,290	3,846	598
JW0758	bioB	1,041	5,564	1,066
JW0759	bioF	1,155	21,424	3,706
JW0760	bioC	756	10,858	2,867
JW0761	bioD	678	10,923	3,204
JW0762	uvrB	2,022	8,054	796
JW0763	ybhK	909	5,824	1,279
JW0764	moaA	990	1,302	265
JW0765	moaB	513	3,016	1,172
JW0766	moaC	486	4,154	1,714
JW0767	moaD	246	1,425	1,149
JW0768	moaE	453	11,144	4,914
JW0769	ybhL	705	4,717	1,331
JW0770	ybhM	714	430	121
JW0771	ybhN	957	6,176	1,282
JW0772	ybhO	1,242	9,195	1,478
JW0773	ybhP	762	18,431	4,828
JW0774	ybhQ	411	5,928	2,887
JW0777	ybhS	1,134	2,916	512

JW0779	ybhG	999	7,514	1,499
JW0780	ybiH	672	5,279	1,565
JW0781	rhlE	1,365	19,915	2,907
JW0783	ybiA	483	6,341	2,618
JW0784	dinG	2,151	13,234	1,230
JW0785	ybiB	963	17,243	3,566
JW0786	ybiC	1,086	6,984	1,279
JW0787	ybiJ	261	2,375	1,816
JW0788	ybiI	267	2,313	1,724
JW0790	fiu	2,283	8,965	785
JW0794	glnQ	723	9,450	2,610
JW0795	glnP	660	8,544	2,585
JW0796	glnH	747	1,238	333
JW0797	dps	504	632	250
JW0798	rhtA	888	2,718	613
JW0799	ompX	516	2,493	967
JW0800	ybiP	1,584	7,143	905
JW0801	mntR	468	8,310	3,553
JW0802	ybiR	1,119	2,399	426
JW0803	ybiS	921	3,695	800
JW0804	ybiT	1,593	15,228	1,910
JW0805	ybiU	1,266	1,541	242
JW0806	ybiV	816	216	53
JW0807	ybiW	2,433	3,785	311
JW0808	ybiY	927	5,779	1,247
JW0810	moeB	750	11,516	3,069
JW0811	moeA	1,236	8,940	1,444
JW0812	iaaA	966	10,943	2,257
JW0815	yliC	921	6,786	1,479
JW0816	yliD	912	9,077	1,975
JW0817	yliE	2,349	1,462	124
JW0818	yliF	1,329	667	102
JW0819	yliG	1,326	3,228	486
JW0820	yliH	384	1,821	947
JW0821	yliI	1,116	10,717	1,922
JW0822	yliJ	627	1,118	356
JW0823	dacC	1,203	16,393	2,721
JW0824	deoR	759	3,019	793
JW0826	cmr	1,233	5,807	936
JW0827	ybjH	285	1,354	945
JW0829	ybjJ	1,209	4,317	716
JW0831	ybjL	1,686	18,826	2,234
JW0832	ybjM	378	763	406
JW0833	grxA	258	529	413
JW0834	ybjC	288	934	647
JW0835	nfsA	723	6,327	1,747
JW0836	rimK	903	4,218	932
JW0837	ybjN	477	2,699	1,128
JW0838	potF	1,113	10,371	1,863
JW0840	potH	954	14,032	2,946
JW0841	potI	846	7,197	1,703
JW0842	ybjO	489	5,693	2,328
JW0843	rumB	1,128	1,407	249
JW0844	artJ	732	651	178
JW0845	artM	669	1,944	582
JW0846	artQ	717	790	221
JW0847	artI	732	3,502	953
JW0848	artP	729	1,922	526
JW0849	ybjP	516	1,017	392
JW0850	ybjQ	324	1,262	777
JW0851	ybjR	831	11,353	2,723

JW0854	ltaE	1,002	6,515	1,307
JW0855	poxB	1,719	10,433	1,214
JW0857	hcp	1,653	18,341	2,220
JW0858	ybjE	900	5,062	1,126
JW0859	aqpZ	696	4,916	1,414
JW0860	ybjD	1,659	25,645	3,092
JW0861	ybjX	993	5,991	1,207
JW0862	macA	1,116	2,241	398
JW0863	macB	1,947	2,700	278
JW0864	cspD	225	0	0
JW0865	clpS	321	2,559	1,599
JW0866	clpA	2,277	3,862	340
JW0867	infA	219	654	593
JW0868	aat	705	4,732	1,344
JW0869	cydC	1,722	25,800	2,997
JW0870	cydD	1,767	6,053	683
JW0871	trxB	966	11,369	2,357
JW0872	lrp	495	642	257
JW0873	ftsK	3,990	582	29
JW0874	lolA	615	2,729	889
JW0875	ycaJ	1,344	3,581	532
JW0876	serS	1,293	1,343	206
JW0878	dmsB	618	7,865	2,541
JW0879	dmsC	864	8,102	1,866
JW0880	ycaC	627	1,856	592
JW0881	ycaD	1,149	8,372	1,458
JW0883	ycaN	909	2,030	448
JW0884	ycaK	591	361	122
JW0885	pflA	741	4,093	1,102
JW0886	pflB	2,283	13,375	1,171
JW0887	focA	858	6,968	1,616
JW0888	ycaO	1,761	0	0
JW0889	ycaP	693	777	226
JW0890	serC	1,089	1,123	206
JW0891	aroA	1,284	7,837	1,218
JW0892	ycaL	765	654	174
JW0893	cmk	684	1,803	523
JW0894	rpsA	1,674	927	111
JW0895	ihfB	285	295	208
JW0897	msbA	1,749	3,598	413
JW0898	lpxK	987	5,407	1,093
JW0899	ycaQ	1,233	4,377	708
JW0900	ycaR	183	86	94
JW0901	kdsB	747	2,682	718
JW0902	ycbJ	894	4,572	1,021
JW0903	ycbC	780	4,866	1,245
JW0904	smtA	786	3,040	773
JW0905	mukF	1,323	1,302	195
JW0906	mukE	678	2,588	763
JW0907	mukB	4,461	2,007	91
JW0908	ycbB	1,848	5,580	603
JW0909	ycbK	549	4,961	1,809
JW0910	ycbL	648	10,659	3,281
JW0911	aspC	1,191	16,901	2,837
JW0912	ompF	1,089	2,066	380
JW0913	asnS	1,401	11,048	1,579
JW0914	pncB	1,203	6,893	1,144
JW0915	pepN	2,613	6,552	503
JW0916	ssuB	768	11,222	2,917
JW0918	ssuD	1,146	3,452	607
JW0919	ssuA	960	5,445	1,138

JW0920	ssuE	576	5,296	1,844
JW0922	ycbR	702	957	270
JW0923	ycbS	2,601	8,204	631
JW0924	ycbT	1,071	2,298	426
JW0925	ycbU	543	1,783	660
JW0928	pyrD	1,011	2,435	479
JW0931	ycbY	2,109	20,860	1,979
JW0932	uup	1,908	5,974	626
JW0933	pqiA	1,254	8,144	1,294
JW0934	pqiB	1,641	801	98
JW0936	rmf	168	669	784
JW0937	fabA	519	8,888	3,428
JW0938	ycbZ	1,761	0	0
JW0939	ycbG	453	1,677	738
JW0940	ompA	1,041	8,785	1,688
JW0941	sulA	510	86	34
JW0942	yccR	630	1,305	411
JW0944	yccF	447	3,396	1,513
JW0945	helD	2,055	17,811	1,731
JW0947	yccT	663	11,706	3,520
JW0952	yccK	330	1,205	730
JW0953	yccA	660	4,298	1,293
JW0954	hyaA	1,119	9,989	1,782
JW0955	hyaB	1,794	15,310	1,707
JW0956	hyaC	708	5,313	1,497
JW0957	hyaD	588	24,097	8,172
JW0958	hyaE	399	4,625	2,308
JW0959	hyaF	858	7,618	1,766
JW0960	appC	1,545	1,587	205
JW0961	appB	1,137	5,496	969
JW0963	appA	1,299	12,685	1,953
JW0964	yccC	2,181	20,577	1,888
JW0966	yccZ	1,140	5,962	1,042
JW0967	ymcA	2,097	2,679	256
JW0968	ymcB	747	1,119	299
JW0969	ymcC	645	4,233	1,310
JW0971	insA	276	4	3
JW0972	insB	504	9	4
JW0974	cspG	213	886	828
JW0975	ymcE	231	200	172
JW0976	gnsA	174	193	222
JW0977	yccM	1,074	9,149	1,701
JW0979	torT	1,029	4,038	785
JW0980	torR	693	7,355	2,116
JW0981	torC	1,173	233	40
JW0982	torA	2,547	9,688	761
JW0983	torD	600	881	297
JW0984	cbpM	306	5,580	3,641
JW0985	cbpA	921	1,942	420
JW0986	yccE	1,257	1,218	196
JW0987	agp	1,242	10,574	1,708
JW0988	yccJ	228	385	336
JW0989	wrbA	597	6,529	2,193
JW0993	ycdI	591	9,600	3,231
JW0994	rarA	801	1,099	273
JW0995	ycdK	387	3,558	1,837
JW0997	ycdM	1,149	9,771	1,698
JW0998	ycdC	639	2,979	931
JW0999	putA	3,963	1,382	70
JW1001	putP	1,509	9,820	1,305
JW1002	ycdN	732	18,212	4,961

JW1003	ycdO	1,128	6,025	1,068
JW1004	ycdB	1,272	502	78
JW1005	phoH	1,065	9,209	1,730
JW1006	ycdP	414	404	197
JW1007	ycdQ	1,326	6,151	921
JW1010	ycdS	2,424	3,116	256
JW1012	insF	867	164	36
JW1015	ycdU	987	1,120	228
JW1017	ycdX	738	9,717	2,635
JW1018	ycdY	555	6,817	2,445
JW1020	csgG	834	559	135
JW1021	csgF	417	622	300
JW1022	csgE	390	143	72
JW1023	csgD	651	412	126
JW1024	csgB	456	572	251
JW1025	csgA	456	966	427
JW1026	csgC	333	73	43
JW1031	ymdA	312	327	211
JW1032	ymdB	534	8,965	3,348
JW1034	mdoC	1,158	6,669	1,149
JW1035	mdoG	1,536	9,671	1,261
JW1037	mdoH	2,544	26,892	2,116
JW1039	msyB	375	1,224	656
JW1040	mdtG	1,227	10,969	1,788
JW1041	lpxL	921	6,532	1,409
JW1042	yceA	1,053	17,205	3,266
JW1043	yceI	576	4,481	1,560
JW1044	yceJ	567	1,033	364
JW1045	yceO	141	78	111
JW1046	solA	1,119	13,265	2,363
JW1048	dinI	246	180	146
JW1049	pyrC	1,047	24,526	4,677
JW1050	yceB	561	685	242
JW1051	grxB	648	7,378	2,260
JW1052	mdtH	1,209	0	0
JW1053	rimJ	585	1,351	462
JW1054	yceH	648	14,376	4,431
JW1055	mviM	924	9,213	1,994
JW1056	mviN	1,536	9,198	1,202
JW1057	flgN	417	940	450
JW1058	flgM	294	1,029	703
JW1059	flgA	660	10,441	3,155
JW1060	flgB	417	4,140	1,968
JW1061	flgC	405	2,444	1,210
JW1062	flgD	696	14,056	4,020
JW1063	flgE	1,209	4,081	677
JW1064	flgF	756	9,978	2,632
JW1065	flgG	783	5,231	1,335
JW1067	flgI	1,098	20,189	3,670
JW1068	flgJ	942	17,424	3,705
JW1069	flgK	1,644	703	86
JW1070	flgL	954	8,072	1,698
JW1071	rne	3,186	2,421	152
JW1072	rluC	960	7,790	1,622
JW1074	yceD	522	1,523	584
JW1075	rpmF	174	1,110	1,286
JW1077	fabH	954	4,753	999
JW1078	fabD	930	19,009	4,077
JW1079	fabG	735	10,440	2,837
JW1080	acpP	237	233	197
JW1081	fabF	1,242	6,560	1,051

JW1082	pabC	810	15,162	3,731
JW1083	yceG	1,023	3,811	746
JW1084	tmk	642	6,598	2,054
JW1085	holB	1,005	4,852	960
JW1086	ycfH	798	5,279	1,324
JW1087	ptsG	1,434	3,592	500
JW1088	fhuE	2,190	5,887	540
JW1089	hinT	360	1,016	566
JW1090	ycfL	378	1,286	684
JW1092	ycfN	825	5,670	1,371
JW1093	nagZ	1,026	4,028	781
JW1095	ndh	1,305	12,747	1,956
JW1096	ycfJ	540	6,062	2,249
JW1098	ycfR	258	349	267
JW1100	mfd	3,447	1,432	83
JW1101	ycfT	1,074	2,306	426
JW1104	lolE	1,245	4,586	737
JW1105	ycfX	912	7,422	1,624
JW1106	cobB	840	14,386	3,428
JW1107	ycfZ	789	428	109
JW1109	potD	1,047	14,271	2,731
JW1110	potC	795	689	171
JW1111	potB	828	7,134	1,720
JW1112	potA	1,137	4,234	745
JW1113	pepT	1,227	6,734	1,095
JW1114	ycfD	1,122	9,837	1,754
JW1115	phoQ	1,461	21,321	2,926
JW1116	phoP	672	1,184	351
JW1117	purB	1,371	8,663	1,264
JW1119	trmU	1,107	3,607	653
JW1120	ymfB	462	4,471	1,927
JW1121	ymfC	654	7,875	2,396
JW1122	icd	1,251	4,377	701
JW1123	ymfD	666	556	166
JW1125	lit	894	518	115
JW1126	intE	1,128	754	133
JW1127	ymfG	246	697	564
JW1128	ymfH	312	1,700	1,082
JW1130	ymfJ	309	1,348	865
JW1131	ymfK	675	1,024	306
JW1133	ymfL	570	911	318
JW1134	ymfM	339	3,830	2,255
JW1135	ymfN	1,368	12,317	1,800
JW1136	ymfR	183	1,047	1,135
JW1137	ymfO	474	3,967	1,677
JW1139	ymfQ	585	7,229	2,460
JW1140	ycfK	630	3,845	1,220
JW1142	tfaE	603	1,727	573
JW1144	pin	555	2,491	896
JW1145	mcrA	834	206	50
JW1147	elbA	324	50	31
JW1148	ycgX	405	240	120
JW1149	ycgE	732	658	180
JW1150	ycgF	1,212	846	141
JW1151	ycgZ	237	113	95
JW1152	ymgA	273	96	69
JW1153	ymgB	267	138	103
JW1154	ymgC	249	183	145
JW1156	ymgF	219	168	154
JW1162	ycgI	213	272	251
JW1163	minE	267	564	415

JW1164	minD	813	381	95
JW1165	minC	696	0	0
JW1166	ycgJ	369	1,598	867
JW1167	ycgK	402	303	150
JW1168	ycgL	327	256	155
JW1169	ycgM	660	2,599	788
JW1172	umuD	420	157	76
JW1173	umuC	1,269	3,944	624
JW1175	nhaB	1,542	2,158	280
JW1176	fadR	720	1,995	556
JW1177	ycgB	1,533	2,050	265
JW1178	dadA	1,299	4,098	627
JW1179	dadX	1,071	5,689	1,064
JW1181	ldcA	915	6,947	1,516
JW1183	ycgR	735	1,558	426
JW1184	ymgE	255	239	187
JW1185	ycgY	441	100	45
JW1186	treA	1,698	2,392	283
JW1193	ycgV	2,868	5,934	415
JW1194	ychF	1,092	7,567	1,389
JW1195	pth	585	3,051	1,039
JW1196	ychH	279	178	128
JW1198	prsA	948	1,135	239
JW1199	ispE	852	2,936	693
JW1200	lolB	624	6,147	1,963
JW1201	hemA	1,257	5,282	842
JW1202	prfA	1,083	28	5
JW1203	prmC	834	4,454	1,063
JW1204	ychQ	393	314	156
JW1205	ychA	810	3,880	958
JW1206	kdsA	855	7,880	1,839
JW1207	chaA	1,101	1,441	260
JW1208	chaB	231	64	53
JW1209	chaC	717	6,402	1,787
JW1210	ychN	354	1,432	816
JW1211	ychP	1,254	29,322	4,676
JW1212	narL	651	3,308	1,014
JW1213	narX	1,797	19,462	2,169
JW1214	narK	1,392	4,147	595
JW1215	narG	3,744	1,240	67
JW1216	narH	1,539	6,100	788
JW1217	narJ	711	10,424	2,937
JW1218	narI	678	3,667	1,083
JW1219	tpr	102	33	66
JW1220	purU	843	4,128	978
JW1221	ychJ	459	2,567	1,115
JW1222	rssA	945	5,111	1,074
JW1223	rssB	1,014	5,505	1,087
JW1224	galU	909	6,075	1,332
JW1225	hns	414	679	323
JW1226	tdk	618	1,482	479
JW1227	ychG	591	3,581	1,206
JW1228	adhE	2,676	4,020	303
JW1229	ychE	648	427	130
JW1235	oppA	1,632	10,227	1,257
JW1236	oppB	921	3,756	814
JW1237	oppC	909	6,807	1,489
JW1238	oppD	1,014	7,639	1,512
JW1239	oppF	1,005	4,171	828
JW1240	yciU	330	288	173
JW1241	cls	1,461	9,333	1,282

JW1242	kch	1,254	1,051	167
JW1243	yciI	297	656	447
JW1245	yciA	399	543	275
JW1246	yciB	540	968	360
JW1247	yciC	744	2,367	631
JW1248	ompW	639	2,847	887
JW1249	yciE	507	984	389
JW1250	yciF	501	155	62
JW1251	yciG	180	316	352
JW1252	trpA	807	3,886	963
JW1253	trpB	1,194	4,691	787
JW1254	trpC	1,359	12,722	1,870
JW1255	trpD	1,596	1,742	218
JW1256	trpE	1,563	14,025	1,794
JW1257	trpL	45	0	0
JW1258	yciV	882	9	2
JW1261	rluB	876	4,318	978
JW1262	btuR	591	4,403	1,482
JW1263	yciK	759	4,972	1,306
JW1264	sohB	1,050	8,732	1,661
JW1265	yciN	252	892	703
JW1266	topA	2,598	2,402	185
JW1267	cysB	975	5,935	1,214
JW1268	acnA	2,676	8,628	645
JW1269	ribA	591	11,554	3,897
JW1270	pgpB	765	4,229	1,104
JW1271	yciS	309	214	139
JW1272	yciM	1,170	10,256	1,757
JW1273	pyrF	738	27,572	7,448
JW1274	yciH	327	1,076	651
JW1275	osmB	219	126	113
JW1276	yciT	750	2,947	784
JW1278	gmr	1,986	1,706	171
JW1279	rnb	1,935	1,001	104
JW1281	fabI	789	4,578	1,156
JW1282	ycjD	354	370	212
JW1283	sapF	807	3,079	764
JW1284	sapD	993	12,856	2,580
JW1285	sapC	891	5,911	1,323
JW1286	sapB	966	2,388	490
JW1287	sapA	1,644	2,577	314
JW1288	ymjA	246	216	171
JW1289	puuP	1,386	5,955	856
JW1291	puuD	765	2,063	541
JW1292	puuR	558	0	0
JW1293	puuC	1,488	16,215	2,174
JW1294	puuB	1,281	4,579	715
JW1295	puuE	1,266	4,557	719
JW1296	pspF	978	3,834	779
JW1297	pspA	669	1,212	362
JW1298	pspB	225	231	203
JW1299	pspC	360	1,474	822
JW1300	pspD	222	596	531
JW1301	pspE	315	993	635
JW1302	ycjM	1,680	887	105
JW1303	ycjN	1,293	2,516	389
JW1304	ycjO	882	13,241	2,986
JW1305	ycjP	843	2,411	573
JW1306	ycjQ	1,053	6,980	1,317
JW1308	ycjS	1,056	5,980	1,129
JW1309	ycjT	2,268	3,934	348

JW1310	ycjU	660	4,558	1,379
JW1311	ycjV	969	4,707	966
JW1312	ompG	906	3,872	854
JW1313	ycjW	999	1,569	314
JW1314	ycjX	1,398	38,286	5,475
JW1315	ycjF	1,062	11,159	2,103
JW1316	tyrR	1,542	9,865	1,280
JW1317	tpx	507	1,929	759
JW1318	ycjG	966	7,141	1,477
JW1319	mpaA	789	2,263	573
JW1321	ycjZ	900	5,304	1,177
JW1322	mppA	1,614	9,986	1,234
JW1323	ynaI	1,032	0	0
JW1324	insH	1,017	0	0
JW1326	ynaJ	258	435	332
JW1327	uspE	951	0	0
JW1328	fnr	753	1,474	392
JW1329	ogt	516	0	0
JW1331	abgB	1,446	631	87
JW1333	abgR	909	1,500	330
JW1334	ydaL	564	784	279
JW1336	ydaN	984	5,455	1,104
JW1337	dbpA	1,374	6,812	991
JW1338	ydaO	936	2,489	532
JW1339	intR	1,236	2,569	414
JW1341	ydaC	210	57	55
JW1343	recT	810	5,328	1,309
JW1344	recE	2,601	1,473	113
JW1345	racC	276	138	99
JW1346	ydaE	171	44	52
JW1347	kil	222	104	94
JW1349	ydaF	156	65	80
JW1351	racR	477	153	64
JW1352	ydaS	297	0	0
JW1353	ydaT	423	500	235
JW1354	ydaU	858	2,987	696
JW1355	ydaV	747	2,387	640
JW1358	trkG	1,458	482	67
JW1359	ynaK	264	293	224
JW1360	ydaY	360	436	240
JW1361	ynaA	1,029	3,175	617
JW1363	insH	981	0	0
JW1366	stfR	3,363	2,515	148
JW1367	tfaR	576	916	319
JW1368	pinR	591	878	298
JW1369	ynaE	267	83	62
JW1370	uspF	435	601	274
JW1371	ompN	1,134	7,938	1,403
JW1372	ydbK	3,525	2,139	121
JW1374	hslJ	423	618	296
JW1375	ldhA	990	2,225	447
JW1376	ydbH	2,640	2,518	191
JW1377	ynbE	186	30	34
JW1379	feaR	906	1,301	287
JW1380	feaB	1,500	29,303	3,894
JW1381	tynA	2,274	1,267	111
JW1382	maoC	2,046	1,734	172
JW1383	paaA	930	8,506	1,830
JW1384	paaB	288	1,055	725
JW1385	paaC	747	11,373	3,034
JW1387	paaE	1,071	2,457	458

JW1388	paaF	768	1,415	368
JW1389	paaG	789	692	177
JW1390	paaH	1,428	17,914	2,518
JW1391	paaI	423	2,368	1,117
JW1392	paaJ	1,206	5,666	938
JW1394	paaX	951	3,389	710
JW1395	paaY	591	3,425	1,150
JW1397	insD	906	35	8
JW1400	insC	411	1	0
JW1401	insI	1,152	5	1
JW1402	ydbA	3,324	6,808	410
JW1403	ydbC	861	10,334	2,389
JW1405	ynbA	606	6,314	2,076
JW1406	ynbB	897	1,516	340
JW1407	ynbC	1,758	6,968	795
JW1408	ynbD	1,293	950	147
JW1409	azoR	606	4,196	1,383
JW1411	ydcF	801	4,541	1,125
JW1412	aldA	1,440	16,251	2,268
JW1413	gapC	252	153	121
JW1416	ydcA	174	116	131
JW1417	trg	1,641	2,442	299
JW1419	ydcJ	1,344	31,777	4,732
JW1420	mdoD	1,626	8,082	1,000
JW1423	rimL	540	0	0
JW1424	ydcK	981	8,392	1,704
JW1425	tehA	993	14,650	2,946
JW1426	tehB	594	1,086	364
JW1427	ydcL	669	2,643	795
JW1430	ydcN	537	6,175	2,288
JW1431	ydcP	1,962	3,502	358
JW1432	yncJ	231	937	803
JW1433	ydcQ	438	1,013	459
JW1434	ydcR	1,407	4,041	576
JW1435	ydcS	1,146	3,086	540
JW1436	ydcT	1,014	13,876	2,730
JW1437	ydcU	942	2,184	462
JW1438	ydcV	795	6,971	1,746
JW1439	ydcW	1,425	6,834	963
JW1441	ydcY	234	257	219
JW1442	ydcZ	450	1,735	778
JW1445	yncC	666	769	232
JW1446	yncD	2,103	4,730	453
JW1447	yncE	1,062	3,146	595
JW1449	yncG	618	1,025	333
JW1451	rhsE	2,049	5,509	537
JW1452	ydcD	483	266	110
JW1453	yncI	747	112	30
JW1455	ydcC	1,137	944	166
JW1456	ydcE	234	89	76
JW1457	yddH	570	2,662	928
JW1458	nhoA	846	2,872	681
JW1459	yddE	894	11,115	2,481
JW1460	narV	681	3,451	1,009
JW1461	narW	696	2,710	783
JW1462	narY	1,545	16,216	2,109
JW1463	narZ	3,741	0	0
JW1464	narU	1,389	7,102	1,021
JW1466	yddJ	336	150	91
JW1467	yddK	957	1,457	304
JW1468	yddL	291	127	89

JW1469	yddG	825	36,837	8,901
JW1470	fdnG	3,048	3,469	229
JW1471	fdnH	885	1,889	428
JW1472	fdnI	654	2,095	639
JW1474	adhP	1,011	13,369	2,648
JW1477	osmC	432	1,405	643
JW1478	ddpF	927	3,984	858
JW1479	ddpD	987	4,884	988
JW1480	ddpC	897	3,556	791
JW1481	ddpB	1,023	0	0
JW1483	ddpX	582	5,316	1,825
JW1484	dos	2,400	8,592	718
JW1486	yddW	1,320	19,422	2,937
JW1487	gadC	1,536	5,185	676
JW1488	gadB	1,401	11,270	1,614
JW1489	pqqL	2,796	3,094	221
JW1490	yddB	2,373	5,752	483
JW1492	ydeM	1,158	1,334	229
JW1494	ydeO	762	427	111
JW1495	ydeP	2,280	12,509	1,096
JW1496	ydeQ	915	1,125	247
JW1497	ydeR	504	545	215
JW1498	ydeS	531	100	37
JW1499	ydeT	1,149	1,143	200
JW1500	hipA	1,323	1	0
JW1501	hipB	267	0	0
JW1502	ydeU	1,401	14,536	2,081
JW1503	ydeK	3,978	1,044	52
JW1504	ydeV	1,593	687	87
JW1505	ydeW	954	7,380	1,542
JW1506	ego	1,536	6,361	828
JW1507	lsrC	1,029	3,164	614
JW1508	lsrD	993	12,428	2,502
JW1509	lsrB	1,023	12,863	2,509
JW1510	lsrF	876	349	79
JW1511	lsrG	291	76	53
JW1512	tam	759	8,892	2,336
JW1514	uxaB	1,452	20,438	2,816
JW1516	yneG	360	1,669	925
JW1517	yneH	927	2,396	518
JW1519	yneJ	882	3,211	727
JW1520	yneK	1,116	1,124	201
JW1521	ydeA	1,191	13,262	2,216
JW1522	marC	666	422	126
JW1525	marB	219	560	512
JW1527	ydeE	1,188	2,164	365
JW1528	ydeH	891	170	38
JW1529	ydeI	393	402	205
JW1530	ydeJ	519	2,700	1,036
JW1531	dcp	2,046	6,471	632
JW1532	ydfG	747	3,775	1,009
JW1533	ydfH	687	12,829	3,721
JW1534	ydfZ	204	356	347
JW1535	ydfI	1,461	8,556	1,171
JW1536	ydfJ	1,284	9,915	1,541
JW1537	ydfK	267	82	62
JW1538	pinQ	591	1,022	345
JW1539	tfaQ	576	887	302
JW1540	stfQ	963	20,545	4,243
JW1541	nohA	570	3,325	1,171
JW1544	cspI	213	0	0

JW1545	ydfP	498	689	280
JW1546	ydfQ	534	1,720	638
JW1547	ydfR	312	146	95
JW1549	cspB	216	123	117
JW1550	cspF	213	89	83
JW1551	ydfT	753	319	85
JW1553	rem	252	94	74
JW1554	hokD	156	75	96
JW1555	relE	288	0	0
JW1556	relB	240	106	91
JW1557	ydfV	306	850	552
JW1558	flxA	333	385	222
JW1559	ydfW	150	58	74
JW1560	ydfX	291	928	642
JW1561	dicC	231	64	56
JW1562	dicA	408	114	55
JW1563	ydfA	156	321	417
JW1564	ydfB	129	23	35
JW1565	ydfC	219	194	181
JW1566	dicB	189	389	415
JW1567	ydfD	192	105	108
JW1568	ydfE	921	3,768	820
JW1570	insD	657	28	9
JW1571	intQ	1,197	1,259	216
JW1572	rspB	1,020	5,606	1,097
JW1573	rspA	1,215	8,358	1,371
JW1574	ynfA	327	240	149
JW1575	ynfB	342	1,377	797
JW1576	speG	561	3,390	1,206
JW1579	ynfE	2,427	1,546	127
JW1581	ynfG	618	1,285	415
JW1586	dgsA	1,221	12,798	2,094
JW1587	ynfL	894	475	105
JW1588	ynfM	1,254	3,569	567
JW1590	ydgD	822	2,843	687
JW1591	mdtI	330	148	90
JW1592	mdtJ	366	242	133
JW1593	ydgG	1,035	5,552	1,073
JW1594	pntB	1,389	9,656	1,385
JW1595	pntA	1,533	9,672	1,259
JW1596	ydgH	945	0	0
JW1597	ydgI	1,383	8,159	1,181
JW1598	folM	723	2,424	667
JW1599	ydgC	336	2,687	1,593
JW1600	rstA	729	1,813	496
JW1601	rstB	1,302	6,914	1,067
JW1602	tus	930	3,908	845
JW1603	fumC	1,404	17,999	2,571
JW1604	fumA	1,647	11,044	1,340
JW1605	manA	1,176	7,746	1,314
JW1606	ydgA	1,509	1,690	224
JW1607	uidC	1,266	3,886	616
JW1608	uidB	1,374	8,545	1,247
JW1609	uidA	1,812	8,074	891
JW1610	uidR	591	3,067	1,035
JW1611	hdhA	768	2,234	579
JW1612	malI	1,029	4,975	961
JW1613	malX	1,593	3,778	474
JW1614	malY	1,173	3,971	675
JW1615	add	1,002	5,151	1,029
JW1617	ydgT	216	104	99

JW1618	ydgK	441	6,126	2,786
JW1619	rsxA	582	950	327
JW1620	rsxB	579	5,764	1,987
JW1621	rsxC	2,223	9,749	876
JW1622	rsxD	1,059	6,241	1,176
JW1623	rsxG	621	10,008	3,212
JW1624	rsxE	696	1,386	400
JW1625	nth	636	1,091	341
JW1626	ydgR	1,503	12,415	1,653
JW1627	gst	606	1,995	656
JW1628	pdxY	864	10,885	2,509
JW1629	tyrS	1,275	7,166	1,119
JW1630	pdxH	657	3,339	1,013
JW1631	ydhA	330	313	187
JW1632	ydhH	1,110	10,583	1,910
JW1633	slyB	468	652	280
JW1635	ydhI	237	559	473
JW1636	ydhJ	858	1,183	276
JW1637	ydhK	2,013	8,661	862
JW1638	sodC	522	0	0
JW1639	ydhF	897	1,145	253
JW1642	nemA	1,098	14,068	2,558
JW1643	gloA	408	914	448
JW1644	rnt	648	7,682	2,368
JW1645	lhr	4,617	2,921	126
JW1646	ydhD	348	4,394	2,515
JW1648	sodB	582	1,383	470
JW1649	ydhP	1,170	6,810	1,166
JW1650	purR	1,026	8,599	1,681
JW1651	ydhB	933	2,917	623
JW1652	ydhC	1,212	9,434	1,551
JW1653	cfa	1,149	2,114	369
JW1654	ribC	642	1,339	413
JW1655	mdtK	1,374	5,958	868
JW1656	ydhQ	1,257	11,818	1,873
JW1657	ydhR	306	362	237
JW1658	ydhS	1,605	2,267	283
JW1659	ydhT	813	1,432	352
JW1660	ydhU	786	1,951	494
JW1662	ydhW	648	3,352	1,026
JW1664	ydhY	627	1,123	360
JW1665	ydhZ	210	107	102
JW1666	pykF	1,413	3,650	517
JW1667	lpp	237	163	135
JW1668	ynhG	1,005	9,790	1,947
JW1669	sufE	417	66	31
JW1670	sufS	1,221	4,793	785
JW1671	sufD	1,272	4,682	736
JW1672	sufC	747	681	184
JW1674	sufA	369	3,322	1,792
JW1675	ydiH	270	95	70
JW1676	ydiI	411	3,608	1,750
JW1677	ydiJ	3,057	9,698	635
JW1678	ydiK	1,113	0	0
JW1679	ydiL	357	134	73
JW1680	ydiM	1,215	843	138
JW1682	ydiB	867	2,036	466
JW1683	aroD	759	1,893	501
JW1684	ydiF	1,596	10,288	1,289
JW1686	ydiP	912	1,964	432
JW1688	ydiR	939	5,274	1,124

JW1689	ydiS	1,290	2,747	429
JW1690	ydiT	294	971	666
JW1692	pps	2,379	6,308	531
JW1693	ydiA	834	3,639	876
JW1694	aroH	1,047	9,486	1,811
JW1695	ydiE	192	78	82
JW1696	ydiU	1,437	16,413	2,288
JW1697	ydiV	714	544	154
JW1698	nlpC	465	5,390	2,316
JW1699	btuD	750	2,551	679
JW1700	btuE	552	274	99
JW1701	btuC	981	17,859	3,633
JW1702	ihfA	300	462	311
JW1703	pheT	2,388	2,689	225
JW1705	pheM	45	0	0
JW1706	rplT	357	2,029	1,139
JW1707	rpmI	198	544	549
JW1709	thrS	1,929	7,899	823
JW1710	arpB	1,416	1,622	229
JW1711	ydiY	759	1,906	503
JW1713	ydiZ	291	184	126
JW1714	yniA	861	49	12
JW1715	yniB	537	2,203	825
JW1716	yniC	669	564	168
JW1718	ydjN	1,392	14,242	2,040
JW1719	ydjO	816	563	137
JW1720	cedA	264	1,540	1,172
JW1721	katE	2,262	5,328	472
JW1722	chbG	750	758	204
JW1723	chbF	1,353	6,707	987
JW1724	chbR	843	2,444	580
JW1725	chbA	351	1,137	645
JW1726	chbC	1,359	6,595	968
JW1727	chbB	321	190	117
JW1728	osmE	339	592	355
JW1729	nadE	828	4,843	1,169
JW1730	ydjQ	888	5,855	1,314
JW1731	ydjR	576	4,377	1,524
JW1732	spy	486	1,879	774
JW1733	astE	969	3,118	645
JW1734	astB	1,344	7,707	1,141
JW1736	astA	1,035	8,894	1,716
JW1737	astC	1,221	16,986	2,780
JW1738	xthA	807	2,102	522
JW1739	ydjX	711	1,728	484
JW1741	ydjZ	708	3,510	991
JW1742	ynjA	549	2,900	1,053
JW1747	ynjF	621	3,331	1,066
JW1748	nudG	408	1,353	666
JW1749	ynjH	273	601	442
JW1750	gdhA	1,344	4,055	607
JW1752	topB	1,962	5,188	532
JW1753	selD	1,044	5,508	1,051
JW1754	ydjA	552	14,055	5,080
JW1755	sppA	1,857	23,653	2,542
JW1756	ansA	1,017	1,112	218
JW1757	pncA	642	3,635	1,124
JW1758	ydjE	1,359	626	92
JW1759	ydjF	759	396	104
JW1760	ydjG	981	2,984	609
JW1762	ydjI	837	7,592	1,813

JW1763	ydjJ	1,044	5,279	1,008
JW1765	ydjL	1,077	13,732	2,548
JW1766	yeaC	273	968	708
JW1767	yeaA	414	1,128	544
JW1768	gapA	996	13,849	2,781
JW1769	yeaD	885	3,640	824
JW1770	yeaE	855	360	84
JW1771	mipA	747	886	238
JW1772	yeaG	1,935	7,080	733
JW1773	yeaH	1,284	2,103	328
JW1774	yeaI	1,476	369	50
JW1776	yeaK	504	6,661	2,637
JW1778	yeaL	447	7,870	3,513
JW1779	yeaM	822	3,369	819
JW1780	yeaN	1,182	1,046	178
JW1781	yeaO	348	5,290	3,024
JW1782	yoaF	255	423	331
JW1784	yeaQ	249	265	215
JW1785	yoaG	183	391	435
JW1786	yeaR	360	641	352
JW1787	yeaS	639	1,355	424
JW1788	yeaT	924	9,606	2,074
JW1789	yeaU	1,086	8,127	1,497
JW1792	yeaX	966	3,470	717
JW1793	rnd	1,128	7,320	1,297
JW1794	fadD	1,686	488	58
JW1795	yeaY	582	3,500	1,209
JW1796	yeaZ	696	5,790	1,652
JW1797	yoaA	1,911	5,190	544
JW1800	yoaH	180	708	788
JW1801	pabB	1,362	3,052	452
JW1802	yeaB	579	2,768	956
JW1803	sdaA	1,365	3,755	546
JW1804	yoaD	1,599	9,829	1,233
JW1805	yoaE	1,557	3,092	399
JW1806	manX	972	7,787	1,598
JW1807	manY	801	1,233	309
JW1808	manZ	861	4,345	1,010
JW1809	yobD	459	484	207
JW1811	rrmA	810	9,021	2,228
JW1812	cspC	210	304	292
JW1813	yobF	144	34	48
JW1814	yebO	288	115	78
JW1815	yobG	144	187	262
JW1816	kdgR	792	4,958	1,246
JW1818	htpX	882	4,581	1,038
JW1819	prc	2,049	875	84
JW1821	yebR	552	5,657	2,047
JW1822	yebS	1,284	1,875	293
JW1823	yebT	2,634	804	61
JW1827	pphA	657	160	48
JW1828	yebY	342	140	83
JW1829	yebZ	873	1,888	433
JW1830	yobA	375	714	378
JW1831	holE	231	350	304
JW1832	yobB	657	1,707	519
JW1833	exoX	663	3,635	1,096
JW1834	ptrB	2,061	2,730	266
JW1835	yebE	660	5,868	1,771
JW1836	yebF	369	878	472
JW1837	yebG	291	129	88

JW1838	purT	1,179	5,275	896
JW1839	eda	642	1,290	403
JW1840	edd	1,812	1,237	137
JW1841	zwf	1,476	9,373	1,274
JW1842	yebK	870	673	154
JW1843	pykA	1,443	3,890	540
JW1844	lpxM	972	4,385	895
JW1847	znuC	756	2,903	763
JW1848	znuB	786	2,985	758
JW1849	ruvB	1,011	5,812	1,146
JW1850	ruvA	612	3,643	1,188
JW1852	ruvC	522	22,913	8,770
JW1853	yebC	741	7,102	1,913
JW1854	nudB	453	845	373
JW1855	aspS	1,773	16,971	1,917
JW1857	yecE	819	1,249	307
JW1859	yecO	744	1,985	535
JW1860	yecP	972	9,994	2,047
JW1861	torZ	2,430	219	18
JW1862	torY	1,101	10,669	1,939
JW1863	cutC	747	3,045	819
JW1865	argS	1,734	13,540	1,565
JW1867	flhE	393	1,746	893
JW1868	flhA	2,079	1,437	138
JW1869	flhB	1,149	11,339	1,978
JW1870	cheZ	645	6,523	2,028
JW1871	cheY	390	2,612	1,342
JW1872	cheB	1,050	1,750	335
JW1873	cheR	861	2,087	487
JW1874	tap	1,602	25,280	3,159
JW1875	tar	1,662	15,888	1,912
JW1876	cheW	504	2,193	870
JW1877	cheA	1,965	3,414	349
JW1878	motB	927	9,496	2,042
JW1879	motA	888	6,399	1,435
JW1880	flhC	579	2,566	884
JW1881	flhD	360	90	51
JW1882	insH	1,017	0	0
JW1884	yecG	429	315	149
JW1886	otsB	801	2,707	673
JW1887	araH	990	1,959	395
JW1888	araG	1,515	9,490	1,255
JW1889	araF	990	6,967	1,407
JW1890	yecI	504	2,146	856
JW1891	yecJ	252	269	221
JW1892	yecR	324	1,548	951
JW1893	ftn	498	1,538	618
JW1894	yecH	240	32	28
JW1895	tyrP	1,212	2,738	454
JW1896	yecA	666	3,390	1,021
JW1897	pgsA	549	5,792	2,109
JW1898	uvrC	1,833	7,167	779
JW1899	uvrY	657	16,890	5,131
JW1900	yecF	225	150	130
JW1901	sdiA	723	8,791	2,427
JW1902	yecC	753	6,000	1,592
JW1903	yecS	669	2,411	716
JW1905	fliY	801	2,416	606
JW1906	fliZ	552	1,207	433
JW1907	fliA	720	4,669	1,295
JW1908	fliC	1,497	11,072	1,474

JW1909	fliD	1,407	8,900	1,262
JW1910	fliS	411	797	390
JW1911	fliT	366	1,776	971
JW1912	amyA	1,488	9,345	1,250
JW1913	yedD	414	2,952	1,414
JW1914	yedE	1,206	16,889	2,795
JW1915	yedF	234	972	824
JW1916	yedK	669	2,737	814
JW1917	yedL	480	2,895	1,194
JW1918	yedN	192	63	64
JW1920	yedM	351	324	186
JW1921	fliE	315	3,840	2,429
JW1922	fliF	1,659	1,208	147
JW1923	fliG	996	5,228	1,049
JW1924	fliH	687	3,303	962
JW1925	fliI	1,374	10,644	1,545
JW1926	fliJ	444	4,401	1,973
JW1927	fliK	1,128	6,781	1,195
JW1928	fliL	465	1,240	528
JW1929	fliM	1,005	6,887	1,368
JW1930	fliN	414	3,219	1,560
JW1932	fliP	738	811	219
JW1933	fliQ	270	565	414
JW1934	fliR	786	2,144	545
JW1935	rcsA	624	48	17
JW1936	dsrB	189	218	230
JW1938	yedP	816	2,121	519
JW1940	yodC	183	202	222
JW1941	yedI	918	3,586	777
JW1942	yedA	921	1,673	361
JW1943	vsr	471	5,898	2,493
JW1944	dcm	1,419	3,895	550
JW1945	yedJ	696	1,405	399
JW1946	yedR	366	1,085	591
JW1948	yedS	210	124	117
JW1949	yedS	405	1,142	558
JW1950	hchA	852	1,776	419
JW1951	yedV	1,359	623	92
JW1953	yedX	414	463	224
JW1954	yedY	1,005	4,961	985
JW1955	yedZ	636	2,013	632
JW1956	yodA	651	237	74
JW1958	yeeI	798	934	236
JW1961	yeeL	705	607	175
JW1962	shiA	1,317	14,186	2,152
JW1963	amn	1,455	8,202	1,123
JW1964	yeeN	717	656	183
JW1965	yeeO	1,644	453	55
JW1966	cbl	951	3,421	718
JW1967	nac	918	984	215
JW1968	erfK	933	2,854	613
JW1969	cobT	1,080	5,649	1,044
JW1970	cobS	744	3,291	880
JW1971	cobU	546	4,031	1,468
JW1972	insH	1,017	0	0
JW1975	insD	906	43	10
JW1978	insC	411	0	0
JW1980	yoeE	261	291	215
JW1982	flu	3,120	4,921	316
JW1983	yeeR	1,533	4,038	527
JW1984	yeeS	447	11,315	5,061

JW1985	yeeT	222	167	148
JW1986	yeeU	369	4,118	2,212
JW1987	yeeV	375	5,048	2,694
JW1988	yeeW	195	358	364
JW1989	yeeX	396	682	339
JW1990	yeeA	1,059	8,154	1,538
JW1991	sbmC	474	2,898	1,226
JW1993	sbcB	1,428	8,413	1,179
JW1994	yeeD	228	307	270
JW1995	yeeE	1,059	8,505	1,603
JW1998	yeeZ	825	8,071	1,952
JW2000	hisL	51	0	0
JW2001	hisG	900	6,038	1,335
JW2002	hisD	1,305	5,692	874
JW2003	hisC	1,071	7,363	1,376
JW2004	hisB	1,068	3,863	724
JW2005	hisH	591	2,877	973
JW2006	hisA	738	2,549	690
JW2007	hisF	777	2,336	598
JW2008	hisI	612	7,696	2,517
JW2010	ugd	1,167	943	163
JW2011	gnd	1,407	2,477	353
JW2012	wbbL	282	115	85
JW2014	insH	1,017	0	0
JW2016	wbbL	474	262	111
JW2017	wbbK	1,119	218	40
JW2018	wbbJ	591	189	63
JW2019	wbbI	993	478	96
JW2020	wbbH	1,167	338	57
JW2021	glf	1,104	326	60
JW2022	rfbX	1,248	431	70
JW2023	rfbC	558	316	112
JW2024	rfbA	882	475	107
JW2025	rfbD	900	4,078	903
JW2026	rfbB	1,086	2,289	422
JW2027	galF	894	5,196	1,168
JW2028	wcaM	1,395	11,237	1,604
JW2029	wcaL	1,221	10,457	1,711
JW2030	wcaK	1,281	7,173	1,121
JW2031	wzxC	1,479	2,957	399
JW2032	wcaJ	1,395	5,424	775
JW2033	cpsG	1,371	5,106	744
JW2034	cpsB	1,437	18,018	2,508
JW2035	wcaI	1,224	13,320	2,181
JW2037	fcl	966	15,050	3,110
JW2038	gmd	1,122	7,209	1,279
JW2039	wcaF	549	3,968	1,444
JW2040	wcaE	747	654	175
JW2041	wcaD	1,218	4,270	699
JW2042	wcaC	1,218	6,015	986
JW2043	wcaB	489	1,785	732
JW2044	wcaA	840	2,804	667
JW2045	wzc	2,163	7,250	674
JW2046	wzb	444	1,598	716
JW2047	wza	1,140	6,071	1,066
JW2049	asmA	1,854	5,081	549
JW2050	dcd	582	6,723	2,297
JW2051	udk	642	2,358	736
JW2052	yegE	3,318	467	28
JW2053	alkA	849	10,664	2,506
JW2054	yegD	1,353	14,512	2,134

JW2055	yegI	1,947	689	71
JW2056	yegJ	462	225	99
JW2057	yegK	762	6,447	1,686
JW2058	yegL	660	3,937	1,187
JW2060	mdtB	3,123	3,273	210
JW2061	mdtC	3,078	2,850	186
JW2062	mdtD	1,416	2,595	367
JW2063	baeS	1,404	3,965	565
JW2064	baeR	723	3,310	914
JW2066	yegQ	1,362	3,314	485
JW2067	ogrK	219	558	506
JW2070	yegS	900	2,091	467
JW2073	insF	867	143	33
JW2074	gatR	339	184	109
JW2075	gatD	1,041	9,202	1,754
JW2076	gatC	1,356	12,495	1,835
JW2077	gatB	285	369	256
JW2078	gatA	321	323	204
JW2081	gatA	315	516	330
JW2082	gatZ	1,263	17,630	2,791
JW2085	yegT	1,278	1,525	238
JW2086	yegU	1,005	5,977	1,189
JW2087	yegV	966	5,585	1,156
JW2088	yegW	747	623	167
JW2090	thiD	801	7,313	1,826
JW2091	thiM	789	2,962	748
JW2092	yohL	273	408	294
JW2093	yohM	825	5,372	1,299
JW2095	yehA	1,035	6,299	1,213
JW2096	yehB	2,481	4,465	359
JW2097	yehC	720	874	242
JW2098	yehD	543	644	238
JW2099	yehE	282	40	28
JW2100	mrp	1,110	13,594	2,449
JW2101	metG	2,034	3,454	340
JW2102	molR	825	1,312	319
JW2105	yehI	3,633	4,920	271
JW2106	yehK	318	184	116
JW2108	yehM	2,280	714	63
JW2110	yehQ	1,845	2,578	281
JW2112	yehS	471	42	17
JW2115	mlrA	732	3,926	1,074
JW2116	yehW	732	14,829	4,039
JW2117	yehX	927	4,378	943
JW2118	yehY	1,158	7,511	1,294
JW2119	yehZ	918	6,518	1,420
JW2120	bglX	2,298	11,464	995
JW2121	dld	1,716	23,829	2,774
JW2124	yohD	579	4,425	1,526
JW2125	yohF	762	19,923	5,219
JW2128	dusC	948	11,152	2,359
JW2129	yohJ	399	858	426
JW2130	yohK	696	7,085	2,031
JW2131	cdd	885	14,154	3,201
JW2132	sanA	720	439	122
JW2133	yeiT	1,239	14,374	2,314
JW2134	yeiA	1,236	11,816	1,912
JW2135	mglC	1,011	2,042	402
JW2136	mglA	1,521	3,284	432
JW2137	mglB	999	21,462	4,283
JW2138	galS	1,041	7,171	1,373

JW2139	yeiB	1,158	4,004	694
JW2140	folE	669	3,919	1,174
JW2141	yeiG	837	5,209	1,240
JW2142	cirA	1,992	11,469	1,150
JW2143	lysP	1,470	9,905	1,345
JW2144	yeiE	882	9,585	2,169
JW2145	yeiH	1,050	0	0
JW2146	nfo	858	2,105	490
JW2147	yeiI	1,089	25,625	4,697
JW2148	yeiJ	1,251	6,238	997
JW2149	rihB	942	12,947	2,754
JW2150	yeiL	660	1,577	478
JW2151	yeiM	1,251	16,508	2,633
JW2152	yeiN	939	2,511	533
JW2153	yeiC	942	5,382	1,141
JW2154	fruA	1,692	16,030	1,904
JW2155	fruK	939	6,814	1,444
JW2156	fruB	1,131	9,140	1,618
JW2157	setB	1,182	15,068	2,541
JW2160	yeiQ	1,467	23,982	3,274
JW2161	yeiR	987	9,294	1,885
JW2162	yeiU	714	1,183	332
JW2163	spr	567	363	127
JW2164	rtn	1,557	3,619	464
JW2165	yejA	1,815	20,438	2,252
JW2166	yejB	1,095	7,424	1,353
JW2167	yejE	1,026	13,677	2,671
JW2168	yejF	1,590	2,526	318
JW2169	yejG	345	250	147
JW2171	rsuA	696	2,348	668
JW2172	yejH	1,761	3,751	425
JW2173	rplY	285	693	480
JW2174	yejK	1,008	7,337	1,454
JW2175	yejL	228	672	591
JW2176	yejM	1,761	1,701	194
JW2179	insH	1,017	0	0
JW2181	narP	648	7,456	2,299
JW2182	ccmH	1,053	20,166	3,835
JW2183	ccmG	558	4,452	1,593
JW2184	ccmF	1,944	21,938	2,258
JW2185	ccmE	480	2,629	1,101
JW2186	ccmD	210	1,718	1,633
JW2187	ccmC	738	4,543	1,228
JW2188	ccmB	663	6,505	1,966
JW2190	napC	603	3,110	1,027
JW2192	napH	864	1,742	402
JW2193	napG	696	1,070	309
JW2194	napA	2,487	11,578	933
JW2195	napD	264	243	187
JW2196	napF	495	2,733	1,106
JW2197	eco	489	1,311	536
JW2198	mqo	1,647	5,555	678
JW2199	yojI	1,644	4,880	594
JW2200	alkB	651	3,030	930
JW2201	ada	1,065	13,576	2,546
JW2203	ompC	1,104	466	85
JW2204	rcsD	2,673	22,060	1,654
JW2205	rcsB	651	1,662	506
JW2213	atoS	1,827	9,470	1,037
JW2214	atoC	1,386	12,596	1,820
JW2215	atoD	663	5,776	1,740

JW2216	atoA	651	3,072	949
JW2217	atoE	1,323	5,062	762
JW2218	atoB	1,185	16,017	2,706
JW2219	yfaP	777	5,157	1,325
JW2220	yfaQ	1,650	9,785	1,192
JW2221	yfaS	486	2,535	1,042
JW2222	yfaS	4,104	740	36
JW2223	yfaT	624	9,029	2,898
JW2224	yfaA	1,689	3,369	396
JW2225	gyrA	2,628	5,937	451
JW2226	ubiG	723	4,227	1,160
JW2227	yfaL	3,753	2,335	124
JW2228	nrdA	2,286	6,044	529
JW2229	nrdB	1,131	4,468	788
JW2230	yfaE	255	1,099	855
JW2231	inaA	651	3,731	1,141
JW2232	yfaH	207	7	7
JW2233	glpQ	1,077	10,651	1,982
JW2234	glpT	1,359	2,955	435
JW2235	glpA	1,629	15,698	1,936
JW2236	glpB	1,260	1,421	226
JW2237	glpC	1,191	2,074	350
JW2238	yfaD	900	4,089	909
JW2239	yfaU	804	34,193	8,471
JW2240	yfaV	1,290	8,032	1,244
JW2241	yfaW	1,218	13,479	2,218
JW2242	yfaX	783	1,528	392
JW2243	yfaY	1,203	7,581	1,259
JW2245	yfaO	426	1,209	570
JW2246	ais	603	2,240	745
JW2248	yfbF	969	5,078	1,044
JW2249	yfbG	1,983	6,956	705
JW2250	yfbH	891	3,871	872
JW2251	arnT	1,653	3,787	459
JW2252	yfbW	336	286	169
JW2254	pmrD	267	149	111
JW2255	menE	1,356	13,703	2,017
JW2256	menC	963	8,275	1,713
JW2257	menB	858	16,669	3,886
JW2258	yfbB	759	13,578	3,565
JW2260	menF	1,296	11,888	1,834
JW2261	elaB	306	1,230	797
JW2262	elaA	462	840	361
JW2263	elaC	918	6,410	1,396
JW2265	yfbK	1,728	1,703	197
JW2266	yfbL	972	7,014	1,451
JW2267	yfbM	504	356	141
JW2268	yfbN	717	612	171
JW2269	yfbO	477	171	72
JW2270	yfbP	852	607	143
JW2271	nuoN	1,278	27,563	4,316
JW2272	nuoM	1,530	2,992	392
JW2273	nuoL	1,842	9,817	1,066
JW2274	nuoK	303	1,380	914
JW2275	nuoJ	555	3,217	1,162
JW2276	nuoI	543	2,333	859
JW2277	nuoH	978	6,414	1,310
JW2278	nuoG	2,733	3,156	231
JW2279	nuoF	1,338	4,938	740
JW2280	nuoE	501	4,745	1,884
JW2283	nuoA	444	4,285	1,921

JW2284	lrhA	939	3,013	639
JW2285	insB	504	19	7
JW2286	insA	276	10	7
JW2287	yfbQ	1,218	5,426	892
JW2288	yfbR	600	4,308	1,437
JW2289	yfbS	1,833	13,621	1,485
JW2291	yfbU	495	2,541	1,031
JW2292	yfbV	456	487	216
JW2293	ackA	1,203	5,716	947
JW2294	pta	2,145	6,488	608
JW2295	yfcC	1,521	11,540	1,517
JW2296	yfcD	543	925	341
JW2298	yfcF	645	2,302	709
JW2299	yfcG	648	3,071	951
JW2300	folX	363	484	264
JW2301	yfcH	894	2,875	645
JW2302	yfcI	891	2,603	583
JW2303	hisP	774	6,174	1,589
JW2304	hisM	717	1,263	350
JW2305	hisQ	687	1,718	502
JW2306	hisJ	783	834	211
JW2307	argT	783	1,676	426
JW2308	ubiX	570	6,074	2,132
JW2309	purF	1,518	20,845	2,749
JW2310	cvpA	489	2,068	854
JW2312	folC	1,269	8,683	1,367
JW2313	accD	915	3,817	837
JW2314	dedA	660	1,335	403
JW2315	truA	813	4,222	1,042
JW2316	usg	1,014	2,828	560
JW2317	pdxB	1,137	11,982	2,109
JW2318	flk	996	9,746	1,945
JW2319	yfcJ	1,179	4,253	724
JW2320	fabB	1,221	3,389	555
JW2322	yfcL	279	54	37
JW2324	yfcA	810	3,092	763
JW2325	mepA	825	2,316	562
JW2326	aroC	1,086	855	158
JW2328	yfcN	552	3,043	1,091
JW2329	yfcO	822	845	205
JW2330	yfcP	540	2,133	786
JW2331	yfcQ	489	279	112
JW2332	yfcR	513	1,079	418
JW2333	yfcS	753	2,568	676
JW2334	yfcT	897	15,831	3,528
JW2335	yfcU	1,737	4,798	556
JW2336	yfcV	564	1,130	401
JW2337	sixA	486	5,796	2,384
JW2338	yfcX	2,145	2,637	246
JW2339	yfcY	1,311	7,942	1,210
JW2340	yfcZ	285	184	126
JW2341	fadL	1,347	2,939	437
JW2342	yfdF	1,059	752	142
JW2343	vacJ	756	6,260	1,663
JW2344	yfdC	933	3,454	738
JW2345	intS	1,158	1,902	330
JW2346	yfdG	363	76	41
JW2347	yfdH	921	338	73
JW2350	yfdK	441	140	65
JW2352	yfdM	276	161	114
JW2355	yfdO	369	2,257	1,227

JW2356	yfdP	447	2,151	961
JW2357	yfdQ	825	1,318	321
JW2358	yfdR	537	1,018	382
JW2359	yfdS	363	395	220
JW2361	dsdC	936	1,204	256
JW2362	dsdX	1,338	3,084	458
JW2363	dsdA	1,329	4,401	661
JW2364	emrY	1,539	966	126
JW2365	emrK	1,164	640	111
JW2366	evgA	615	177	58
JW2367	evgS	3,594	504	29
JW2368	yfdE	1,146	5,923	1,036
JW2369	yfdV	945	2,104	442
JW2370	oxc	1,695	13,286	1,567
JW2371	frc	1,251	4,678	750
JW2372	yfdX	636	1,485	468
JW2373	ypdI	276	50	38
JW2374	yfdY	243	133	108
JW2375	ddg	921	1,738	376
JW2376	yfdZ	1,239	3,919	633
JW2378	ypdB	735	3,814	1,040
JW2379	ypdC	858	2,101	490
JW2380	ypdD	2,496	7,252	583
JW2381	ypdE	1,038	2,854	546
JW2382	ypdF	1,086	618	115
JW2383	ypdG	1,248	3,709	595
JW2385	glk	966	6,164	1,276
JW2386	yfeO	1,257	5,456	864
JW2387	ypeC	327	136	82
JW2388	mntH	1,239	10,033	1,619
JW2389	nupC	1,203	2,899	484
JW2393	yfeC	345	409	238
JW2394	yfeD	393	243	126
JW2395	gltX	1,416	4,040	569
JW2396	xapR	885	5,396	1,210
JW2397	xapB	1,257	3,607	574
JW2398	xapA	834	4,845	1,152
JW2399	yfeN	765	1,414	372
JW2400	yfeR	927	4,586	987
JW2403	ligA	2,016	3,838	381
JW2404	zipA	987	2,356	478
JW2406	cysZ	762	5,582	1,456
JW2407	cysK	972	2,149	440
JW2408	ptsH	258	292	226
JW2409	ptsI	1,728	2,731	320
JW2410	crr	510	590	231
JW2411	pdxK	852	8,849	2,073
JW2412	yfeK	375	171	92
JW2413	yfeS	735	343	94
JW2414	cysM	912	11,839	2,591
JW2415	cysA	1,098	8,247	1,502
JW2416	cysW	876	1,817	416
JW2417	cysU	834	3,130	753
JW2418	cysP	1,017	3,581	705
JW2420	yfeT	858	8,262	1,926
JW2421	yfeU	897	7,388	1,641
JW2422	murP	1,425	3,643	512
JW2424	yfeX	900	6,590	1,465
JW2425	yfeY	576	2,865	988
JW2426	yfeZ	450	1,970	878
JW2427	ypeA	426	1,041	489

JW2428	amiA	870	1,831	418
JW2429	hemF	900	2,240	497
JW2430	yfeG	1,053	1,845	351
JW2431	yffI	501	3,303	1,315
JW2432	eutL	660	1,969	598
JW2433	eutC	888	3,071	691
JW2434	eutB	1,362	3,374	494
JW2435	eutA	1,404	3,180	455
JW2436	eutH	1,227	3,893	637
JW2437	eutG	1,188	3,531	596
JW2438	eutJ	837	6,587	1,570
JW2439	eutE	1,404	10,891	1,553
JW2440	cchB	288	678	463
JW2441	cchA	294	1,308	884
JW2442	eutI	1,017	9,516	1,870
JW2443	eutT	804	3,840	954
JW2444	eutQ	702	7,248	2,062
JW2445	eutP	480	1,588	654
JW2446	ypfE	336	308	180
JW2447	maeB	2,280	7,542	661
JW2448	talA	951	3,219	680
JW2449	tktB	2,004	8,367	835
JW2450	ypfG	1,044	3,041	585
JW2451	yffH	576	385	133
JW2452	aegA	1,980	22,118	2,233
JW2453	narQ	1,701	8,116	956
JW2454	acrD	3,114	7,334	472
JW2455	yffB	357	691	388
JW2456	dapE	1,128	8,389	1,486
JW2457	ypfN	201	86	84
JW2459	ypfI	2,016	7,936	788
JW2460	ypfJ	864	3,364	774
JW2461	purC	714	2,109	590
JW2462	nlpB	1,035	6,559	1,260
JW2463	dapA	879	7,322	1,663
JW2464	gcvR	573	5,902	2,058
JW2465	bcp	471	831	353
JW2466	hyfA	618	1,402	455
JW2467	hyfB	2,019	5,671	562
JW2468	hyfC	948	13,695	2,880
JW2469	hyfD	1,440	2,518	349
JW2470	hyfE	651	1,879	577
JW2471	hyfF	1,581	7,671	971
JW2472	hyfG	1,668	1	0
JW2473	hyfH	546	4,543	1,659
JW2475	hyfJ	414	2,206	1,068
JW2476	hyfR	2,013	5,989	595
JW2477	focB	849	8,119	1,913
JW2478	yfgO	1,062	3,888	731
JW2479	yfgC	1,464	3,680	502
JW2480	yfgD	360	1,977	1,097
JW2482	uraA	1,290	4,150	641
JW2483	upp	627	4,332	1,378
JW2484	purM	1,038	9,726	1,871
JW2485	purN	639	3,730	1,164
JW2486	ppk	2,067	3,454	334
JW2487	ppx	1,542	12,955	1,680
JW2488	yfgF	2,244	5,144	458
JW2490	yfgI	540	2,950	1,099
JW2491	guaA	1,578	14,144	1,801
JW2493	xseA	1,371	7	1

JW2496	yfgL	1,179	12,550	2,123
JW2497	yfgM	621	2,808	899
JW2498	hisS	1,275	8,860	1,395
JW2499	ispG	1,119	12,766	2,274
JW2500	yfgA	1,014	3,451	680
JW2501	yfgB	1,155	4,190	725
JW2502	ndk	432	144	67
JW2503	pbpC	2,313	11,977	1,041
JW2504	yfhM	4,962	1,002	41
JW2505	sseA	846	2,456	579
JW2507	pepB	1,284	2,961	462
JW2508	yfhJ	201	112	115
JW2509	fdx	336	571	340
JW2510	hscA	1,851	5,053	543
JW2511	hscB	516	2,291	882
JW2512	iscA	324	57	35
JW2513	iscU	387	688	361
JW2514	iscS	1,215	5,501	907
JW2515	iscR	489	4,015	1,641
JW2516	yfhQ	741	5,223	1,408
JW2517	suhB	804	11,275	2,800
JW2518	yfhR	882	3,744	851
JW2520	hcaT	1,140	10,584	1,851
JW2521	hcaR	891	11,640	2,613
JW2522	hcaE	1,362	3,706	546
JW2523	hcaF	519	2,190	839
JW2524	hcaC	321	538	331
JW2525	hcaB	813	7,348	1,810
JW2526	hcaD	1,203	4,065	677
JW2527	yphA	495	2,471	998
JW2528	yphB	873	791	181
JW2530	yphD	999	3,753	749
JW2531	yphE	1,512	33,653	4,448
JW2532	yphF	984	2,218	454
JW2535	glyA	1,254	8,920	1,421
JW2536	hmp	1,191	2,954	494
JW2537	glnB	339	822	483
JW2538	yfhA	1,335	3,519	528
JW2539	yfhG	714	4,048	1,135
JW2541	purL	3,888	645	33
JW2542	yfhD	1,419	4,511	635
JW2543	tadA	537	4,708	1,747
JW2545	yfhH	849	2,302	542
JW2546	yfhL	261	30	23
JW2547	acpS	381	2,683	1,399
JW2548	pdxJ	732	2,217	606
JW2549	recO	729	0	0
JW2550	era	906	4,519	996
JW2551	rnc	681	3,660	1,071
JW2552	lepB	975	3,554	730
JW2553	lepA	1,800	6,346	706
JW2554	rseC	480	6,572	2,728
JW2555	rseB	957	3,955	822
JW2556	rseA	651	3,589	1,101
JW2557	rpoE	576	30	11
JW2558	nadB	1,623	17,552	2,159
JW2559	yfiC	738	4,076	1,103
JW2560	srmB	1,335	8,284	1,241
JW2561	yfiE	882	3,728	845
JW2562	yfiK	588	3,189	1,077
JW2563	yfiD	384	383	201

JW2564	ung	690	3,888	1,129
JW2565	yfiF	1,038	9,533	1,827
JW2566	trxC	420	1,975	940
JW2568	yfiQ	2,661	4,456	336
JW2569	pssA	1,356	9,739	1,433
JW2570	yfiM	273	2,253	1,646
JW2571	kgtP	1,299	3,122	481
JW2573	clpB	2,574	6,106	475
JW2575	yfiH	732	9,078	2,467
JW2576	rluD	981	11,043	2,256
JW2577	yfiO	738	10,088	2,733
JW2578	yfiA	342	1,724	1,007
JW2579	pheL	48	0	0
JW2580	pheA	1,161	12,989	2,232
JW2581	tyrA	1,122	1,314	236
JW2582	aroF	1,071	10,646	1,981
JW2584	yfiR	519	1,390	540
JW2585	yfiN	1,227	0	0
JW2586	yfiB	483	2,612	1,078
JW2587	rplS	348	547	316
JW2588	trmD	768	2,023	530
JW2590	rpsP	249	1,194	960
JW2592	ypjD	867	5,151	1,189
JW2594	grpE	594	2,552	860
JW2596	uppS	879	4,562	1,037
JW2598	smpA	342	1,930	1,118
JW2599	yfjF	291	701	480
JW2600	yfjG	477	434	181
JW2601	smpB	483	3,762	1,549
JW2602	intA	1,242	6,041	969
JW2603	yfjH	957	582	122
JW2604	alpA	213	96	92
JW2605	yfjI	1,410	943	134
JW2607	yfjJ	627	374	120
JW2608	yfjK	2,190	10,413	956
JW2609	yfjL	1,617	2,473	303
JW2610	yfjM	264	98	73
JW2611	yfjN	1,074	3,433	643
JW2614	yfjQ	822	3,156	770
JW2615	yfjR	702	3,860	1,097
JW2618	yfjT	468	639	272
JW2619	yfjU	315	351	221
JW2621	yfjV	201	1,131	1,127
JW2623	yfjW	1,704	408	48
JW2624	yfjX	459	3,809	1,653
JW2625	yfjY	483	3,548	1,472
JW2626	yfjZ	318	1,049	655
JW2627	ypjF	330	1,991	1,207
JW2629	ypjB	792	397	99
JW2631	ygaR	687	89	27
JW2633	yqaD	273	127	94
JW2635	ygaF	1,269	9,555	1,502
JW2636	gabD	1,449	9,214	1,274
JW2637	gabT	1,281	10,754	1,679
JW2638	gabP	1,401	11,098	1,580
JW2639	csiR	663	3,115	939
JW2640	ygaU	450	1,623	719
JW2641	yqaE	159	773	968
JW2642	ygaV	300	481	314
JW2643	ygaP	525	1,104	422
JW2644	stpA	405	157	76

JW2645	ygaW	450	2,029	899
JW2646	ygaC	345	1,446	834
JW2647	ygaM	342	5,926	3,463
JW2648	nrdH	246	361	292
JW2649	nrdI	411	3,122	1,516
JW2650	nrdE	2,145	10,333	967
JW2651	nrdF	960	4,532	942
JW2652	proV	1,203	3,833	636
JW2653	proW	1,065	14,825	2,783
JW2654	proX	993	14,864	2,994
JW2655	ygaX	267	1,245	924
JW2657	ygaZ	738	5,914	1,597
JW2658	ygaH	336	5,700	3,379
JW2659	mprA	531	1,252	468
JW2660	emrA	1,173	4,541	773
JW2661	emrB	1,539	3,869	504
JW2662	luxS	516	3,242	1,256
JW2663	gshA	1,557	17,404	2,231
JW2664	yqaA	429	11	5
JW2665	yqaB	567	2,448	858
JW2666	csrA	186	406	441
JW2667	alaS	2,631	5,631	429
JW2668	recX	501	2,144	851
JW2669	recA	1,062	3,261	615
JW2670	ygaD	498	6,546	2,617
JW2671	mltB	1,086	0	0
JW2673	srlB	372	3,060	1,636
JW2674	srlD	780	3,663	936
JW2675	gutM	360	2,321	1,290
JW2676	srlR	774	1,610	418
JW2680	norV	1,440	14,301	1,986
JW2681	norW	1,134	18,480	3,252
JW2683	hydN	528	5,453	2,067
JW2686	ascB	1,425	4,172	586
JW2687	hycI	471	3,382	1,441
JW2688	hycH	411	5,514	2,684
JW2689	hycG	768	2,937	767
JW2690	hycF	543	2,364	874
JW2691	hycE	1,710	4,909	572
JW2692	hycD	924	1,271	275
JW2693	hycC	1,827	8,337	916
JW2694	hycB	612	4,153	1,356
JW2695	hycA	462	4,891	2,120
JW2696	hypA	351	3,799	2,151
JW2697	hypB	873	16,651	3,800
JW2698	hypC	273	2,118	1,558
JW2699	hypD	1,122	16,308	2,902
JW2700	hypE	969	5,791	1,192
JW2701	fhlA	2,079	2,547	246
JW2702	ygbA	354	855	480
JW2703	mutS	2,562	4,882	381
JW2704	pphB	657	1,656	494
JW2705	ygbI	798	1,634	412
JW2706	ygbJ	909	6,145	1,350
JW2707	ygbK	1,167	18,242	3,128
JW2708	ygbL	639	3,502	1,092
JW2709	ygbM	777	3,560	923
JW2710	ygbN	1,365	11,933	1,746
JW2712	nlpD	1,140	543	95
JW2713	pcm	627	6,699	2,134
JW2714	surE	762	5,616	1,475

JW2715	truD	1,050	9,385	1,785
JW2716	ispF	480	4,353	1,814
JW2717	ispD	711	3,857	1,085
JW2718	ftsB	312	1,918	1,232
JW2719	ygbE	324	367	221
JW2720	cysC	606	5,790	1,897
JW2721	cysN	1,428	13,111	1,833
JW2722	cysD	909	11,831	2,597
JW2723	iap	1,038	12,679	2,435
JW2725	ygbT	918	5,778	1,249
JW2726	ygcH	600	1,424	475
JW2728	ygcJ	1,092	1,264	231
JW2729	ygcK	483	647	270
JW2730	ygcL	1,509	897	119
JW2731	ygcB	2,667	1,940	145
JW2732	cysH	735	1,880	517
JW2733	cysI	1,713	23,892	2,797
JW2734	cysJ	1,800	18,039	2,004
JW2735	ygcM	366	882	480
JW2736	ygcN	1,272	17,578	2,766
JW2737	ygcO	261	3,611	2,749
JW2738	ygcP	576	4,648	1,604
JW2746	yqcE	1,278	18,324	2,872
JW2748	ygcF	672	2,795	828
JW2750	eno	1,299	8,290	1,277
JW2751	pyrG	1,638	4,663	572
JW2752	mazG	792	4,944	1,249
JW2753	chpA	336	228	135
JW2754	chpR	249	479	390
JW2755	relA	2,235	3,147	283
JW2756	rumA	1,302	17,710	2,722
JW2757	barA	2,757	681	50
JW2758	gudD	1,341	14,485	2,156
JW2759	gudX	1,341	17,583	2,621
JW2760	gudP	1,353	10,083	1,485
JW2761	yqcA	450	2,356	1,052
JW2762	yqcB	783	9,171	2,341
JW2763	yqcC	330	471	283
JW2764	syd	546	7,617	2,787
JW2765	yqcD	849	5,801	1,360
JW2766	ygdH	1,365	2,841	417
JW2767	sdaC	1,290	3,094	478
JW2768	sdaB	1,368	6,650	969
JW2770	fucO	1,152	10,525	1,825
JW2771	fucA	648	1,306	403
JW2772	fucP	1,317	7,854	1,192
JW2773	fucI	1,776	2,346	264
JW2774	fucK	1,449	13,570	1,878
JW2775	fucU	423	924	435
JW2776	fucR	732	3,506	957
JW2777	ygdE	1,101	25,545	4,637
JW2778	ygdD	396	4,839	2,430
JW2779	gcvA	918	7,272	1,585
JW2781	csdA	1,206	17,275	2,861
JW2782	ygdK	444	4,922	2,214
JW2783	ygdL	807	18,750	4,635
JW2784	mltA	1,098	7,142	1,299
JW2786	argA	1,332	9,506	1,430
JW2787	recD	1,827	4,929	542
JW2788	recB	3,543	1,039	59
JW2789	ptr	2,889	4,905	338

JW2790	recC	3,369	3,389	201
JW2791	ppdC	324	1,275	796
JW2794	ppdA	471	567	241
JW2795	thyA	795	5,338	1,342
JW2796	lgt	876	10,363	2,361
JW2797	ptsP	2,247	2,483	221
JW2798	nudH	531	1,347	507
JW2799	mutH	690	3,026	879
JW2800	ygdQ	714	1,943	544
JW2801	ygdR	219	74	66
JW2802	tas	1,041	24,031	4,615
JW2803	ygeD	1,194	15,540	2,603
JW2804	aas	2,160	26,396	2,446
JW2805	galR	1,032	11,231	2,173
JW2806	lysA	1,263	13,112	2,078
JW2807	lysR	936	8,267	1,766
JW2808	ygeA	693	2,655	762
JW2809	araE	1,419	5,540	777
JW2810	kduD	762	4,482	1,172
JW2811	kduI	837	1,731	412
JW2813	yqeG	1,230	1,581	257
JW2815	yqeI	810	1,019	249
JW2817	yqeK	426	351	165
JW2818	ygeF	447	275	121
JW2819	ygeG	492	423	171
JW2820	ygeH	1,377	639	93
JW2824	ygeL	156	29	35
JW2826	insD	906	38	8
JW2829	insC	411	1	1
JW2831	ygeP	300	156	105
JW2833	ygeR	756	2,191	574
JW2835	xdhB	879	5,638	1,279
JW2836	xdhC	480	5,011	2,079
JW2837	ygeV	1,779	14,966	1,684
JW2839	ygeX	1,197	8,885	1,477
JW2840	ygeY	1,212	6,540	1,080
JW2841	hyuA	1,386	5,876	844
JW2842	yqeA	933	8,601	1,843
JW2843	yqeB	1,626	3,075	379
JW2845	ygfJ	579	534	186
JW2847	ssnA	1,329	3,308	500
JW2848	ygfM	780	0	0
JW2849	xdhD	2,871	11,110	773
JW2850	ygfO	1,401	1,521	218
JW2857	idi	549	2,097	758
JW2858	lysS	1,518	6,772	892
JW2860	recJ	1,734	11,207	1,296
JW2861	dsbC	711	2,567	716
JW2862	xerD	897	2,797	629
JW2863	fldB	522	1,620	613
JW2864	ygfX	408	6,497	3,189
JW2865	ygfY	267	2,879	2,144
JW2866	ygfZ	981	5,072	1,037
JW2867	yqfA	660	3,756	1,135
JW2868	yqfB	312	764	492
JW2869	bglA	1,440	13,494	1,872
JW2870	ygfF	744	9,197	2,477
JW2871	gcvP	2,874	7,333	511
JW2872	gcvH	390	3,626	1,857
JW2873	gcvT	1,095	9,651	1,765
JW2874	visC	1,203	7,104	1,181

JW2875	ubiH	1,179	8,691	1,471
JW2876	pepP	1,326	5,740	865
JW2878	zapA	330	775	464
JW2879	ygfA	549	0	0
JW2880	serA	1,233	7,769	1,261
JW2882	yqfE	231	533	461
JW2883	argP	894	5,590	1,252
JW2884	yliK	2,145	8,841	821
JW2885	argK	996	7,997	1,604
JW2886	ygfG	786	5,290	1,337
JW2887	ygfH	1,479	12,479	1,685
JW2889	yggE	741	5,946	1,600
JW2890	argO	636	2,498	784
JW2891	mscS	861	5,123	1,190
JW2892	fbaA	1,080	7,642	1,420
JW2893	pgk	1,164	11,087	1,906
JW2894	epd	1,020	4,959	972
JW2895	yggC	714	1,671	471
JW2896	yggD	510	1,511	587
JW2897	yggF	966	7,348	1,513
JW2900	cmtA	1,389	6,218	897
JW2901	cmtB	444	387	175
JW2903	yggG	759	1,938	511
JW2904	speB	921	5,911	1,285
JW2905	speA	1,977	19,243	1,953
JW2906	yqgB	147	222	300
JW2907	yqgC	216	969	888
JW2908	yqgD	252	457	361
JW2909	metK	1,155	7,797	1,354
JW2910	galP	1,395	13,174	1,899
JW2911	sprT	498	2,543	1,023
JW2912	endA	708	2,889	819
JW2913	yggJ	732	2,643	718
JW2914	gshB	951	13,146	2,763
JW2915	yqgE	564	2,578	915
JW2916	yqgF	417	7,353	3,517
JW2917	yggR	981	18,858	3,840
JW2918	yggS	705	3,265	926
JW2919	yggT	567	12,154	4,281
JW2921	yggV	594	4,221	1,420
JW2922	yggW	1,137	5,201	918
JW2923	yggM	1,008	1,443	288
JW2924	ansB	1,047	9,024	1,728
JW2925	yggN	720	4,378	1,218
JW2926	yggL	327	1,560	960
JW2927	yggH	720	5,944	1,645
JW2928	mutY	1,053	5,414	1,028
JW2929	yggX	276	485	347
JW2932	nupG	1,257	4,014	634
JW2934	yqgA	708	2,350	661
JW2935	yghD	537	3,151	1,170
JW2938	yghG	411	936	455
JW2939	pppA	810	2,370	588
JW2942	yghK	1,683	5,937	702
JW2943	glcB	2,172	3,754	346
JW2944	glcG	405	4,093	2,008
JW2946	glcD	1,500	11,653	1,556
JW2947	glcC	765	3,571	931
JW2949	insH	1,017	0	0
JW2952	yghR	759	3,063	805
JW2954	yghT	693	5	2

JW2955	pitB	1,500	9,752	1,303
JW2956	gss	1,860	11,586	1,246
JW2958	hybG	249	1,428	1,141
JW2960	hybE	489	1,875	758
JW2961	hybD	495	1,149	462
JW2962	hybC	1,704	19,297	2,266
JW2964	hybA	987	14,322	2,901
JW2965	hybO	1,119	4,877	870
JW2966	yghW	288	997	692
JW2970	yghZ	1,041	4,454	854
JW2971	yqhA	495	492	199
JW2972	yghA	885	2,356	531
JW2973	exbD	426	4,163	1,954
JW2974	exbB	735	3,901	1,056
JW2975	metC	1,188	4,433	744
JW2976	yghB	660	1,735	528
JW2978	yqhD	1,164	3,836	658
JW2982	yqhH	258	1,143	879
JW2985	sufI	1,413	7,965	1,123
JW2986	plsC	738	2,537	686
JW2987	parC	2,259	3,186	284
JW2988	ygiS	1,608	3,608	448
JW2989	ygiT	396	496	251
JW2990	ygiU	297	35	23
JW2992	ygiW	393	2,103	1,065
JW2993	qseB	660	6,616	1,990
JW2994	qseC	1,350	3,950	587
JW2995	ygiZ	333	345	211
JW2996	mdaB	582	1,823	623
JW2997	ygiN	315	1,706	1,079
JW2998	parE	1,893	4,581	486
JW2999	yqiA	582	3,828	1,313
JW3000	cpdA	828	3,264	785
JW3001	yqiB	423	2,556	1,202
JW3002	nudF	630	3,610	1,143
JW3006	ygiC	1,161	3,158	545
JW3007	zupT	816	4,490	1,098
JW3008	ygiE	774	0	0
JW3009	ribB	654	1,285	392
JW3011	ygiL	552	345	126
JW3012	insC	411	0	0
JW3013	insD	906	37	8
JW3021	glgS	201	53	51
JW3022	yqiJ	630	671	213
JW3023	yqiK	1,662	3,249	391
JW3024	rfaE	1,434	2,187	307
JW3025	glnE	2,841	4,374	308
JW3026	ygiF	1,302	1,707	260
JW3027	htrG	621	2,012	653
JW3028	cca	1,239	8,214	1,322
JW3029	bacA	822	2,314	560
JW3030	folB	369	5,823	3,138
JW3031	ygiH	618	1,862	601
JW3032	ygiP	933	13,414	2,862
JW3033	ttdA	912	4,576	1,005
JW3034	ttdB	606	8,243	2,708
JW3035	ygjE	1,464	9,066	1,236
JW3036	ygjD	1,014	5,017	992
JW3037	rpsU	216	363	331
JW3038	dnaG	1,746	15,242	1,747
JW3039	rpoD	1,842	20,440	2,221

JW3040	ygjF	507	1,777	698
JW3041	yqjH	765	4,441	1,158
JW3042	yqjI	624	5,054	1,624
JW3043	aer	1,521	8,021	1,055
JW3045	ygjH	333	2,538	1,523
JW3046	ebgR	984	2,443	495
JW3048	ebgC	450	1,742	775
JW3050	ygjJ	1,071	4,690	868
JW3051	ygjK	2,352	8,355	715
JW3052	fadH	2,019	7,994	793
JW3053	ygjM	417	2,565	1,224
JW3054	ygjN	315	262	167
JW3057	ygjQ	693	4,320	1,240
JW3058	ygjR	1,005	9,950	1,970
JW3060	sstT	1,245	2,133	343
JW3061	ygjV	552	4,154	1,501
JW3062	uxaA	1,488	3,849	521
JW3063	uxaC	1,413	3,852	545
JW3064	exuT	1,419	2,685	377
JW3065	exuR	777	4,457	1,148
JW3066	yqjA	663	745	224
JW3067	yqjB	384	3,524	1,828
JW3069	yqjD	306	128	85
JW3070	yqjE	405	2,145	1,064
JW3071	yqjK	300	1,505	1,001
JW3073	yqjG	987	1,739	351
JW3074	yhaH	366	4,484	2,455
JW3075	yhaI	357	259	144
JW3076	yhaJ	897	3,823	856
JW3077	yhaK	702	1,247	358
JW3087	tdcC	1,332	3,680	554
JW3088	tdcB	990	3,984	801
JW3089	tdcA	939	1,144	243
JW3091	yhaB	540	875	324
JW3092	yhaC	1,188	601	102
JW3093	garK	1,227	2,848	463
JW3095	garL	771	850	220
JW3096	garP	1,335	4,961	744
JW3097	garD	1,572	6,852	871
JW3098	sohA	336	874	519
JW3099	yhaV	465	0	0
JW3100	agaR	810	4,642	1,147
JW3101	kbaZ	1,281	18,261	2,843
JW3102	agaV	474	2,077	873
JW3103	agaW	402	4,710	2,330
JW3105	agaS	1,155	14,312	2,472
JW3106	kbaY	861	18,530	4,311
JW3107	agaB	477	741	311
JW3108	agaC	804	5,399	1,338
JW3109	agaD	792	3,458	875
JW3110	agaI	756	5,355	1,419
JW3111	yraH	585	965	329
JW3112	yraI	696	1,744	498
JW3113	yraJ	2,517	4,000	318
JW3114	yraK	1,092	4,492	825
JW3115	yraL	861	5,571	1,290
JW3116	yraM	2,037	8,685	857
JW3117	yraN	396	4,190	2,114
JW3118	yraO	591	752	252
JW3119	yraP	576	6,540	2,280
JW3120	yraQ	1,041	33,000	6,322

JW3123	yhbP	444	2,120	956
JW3124	yhbQ	303	2,149	1,421
JW3125	yhbS	504	5,119	2,022
JW3126	yhbT	525	2,286	868
JW3127	yhbU	996	5,901	1,180
JW3129	yhbW	1,008	8,334	1,657
JW3130	mtr	1,245	14,461	2,315
JW3132	nlpI	885	3,914	888
JW3134	rpsO	270	573	426
JW3135	truB	945	1,353	286
JW3136	rbfA	402	2,322	1,156
JW3137	infB	2,673	3,208	241
JW3138	nusA	1,488	6,906	929
JW3140	argG	1,344	9,703	1,447
JW3142	secG	333	870	516
JW3143	glmM	1,338	27,246	4,057
JW3144	folP	849	17,159	4,035
JW3145	ftsH	1,935	3,905	408
JW3146	rrmJ	630	3,590	1,140
JW3147	yhbY	294	466	320
JW3148	greA	477	3,290	1,373
JW3149	dacB	1,434	11,184	1,559
JW3150	obgE	1,173	5,341	908
JW3151	yhbE	966	6,346	1,309
JW3152	rpmA	258	979	754
JW3153	rplU	312	454	289
JW3154	ispB	972	8,403	1,723
JW3155	sfsB	279	274	198
JW3156	murA	1,260	11,073	1,759
JW3157	yrbA	255	426	332
JW3159	yrbC	636	2,614	820
JW3160	yrbD	552	2,289	829
JW3161	yrbE	783	11,890	3,016
JW3162	yrbF	810	20,782	5,128
JW3163	yrbG	978	10,022	2,045
JW3164	kdsD	987	45,517	9,179
JW3165	kdsC	567	3,001	1,056
JW3166	yrbK	576	1,092	381
JW3167	yhbN	558	579	209
JW3168	yhbG	726	15,093	4,132
JW3169	rpoN	1,434	2,968	412
JW3170	yhbH	288	660	459
JW3171	ptsN	492	3,805	1,529
JW3172	yhbJ	855	3,466	813
JW3173	npr	273	959	705
JW3174	yrbL	633	4,811	1,517
JW3175	mtgA	729	2,409	660
JW3176	elbB	654	8,703	2,648
JW3178	yhcC	930	8,109	1,744
JW3179	gltB	4,554	639	28
JW3180	gltD	1,419	9,433	1,323
JW3181	gltF	765	5,870	1,524
JW3182	yhcA	675	666	197
JW3183	yhcD	2,382	1,014	86
JW3184	yhcE	480	436	185
JW3185	insH	1,017	0	0
JW3187	yhcE	249	354	284
JW3188	yhcF	717	1,203	334
JW3189	yhcG	1,128	4,348	770
JW3190	yhcH	465	1,970	850
JW3192	nanE	690	6,631	1,917

JW3193	nanT	1,491	19,771	2,648
JW3194	nanA	894	1,933	432
JW3195	nanR	792	2,873	723
JW3196	dcuD	1,368	5,055	734
JW3197	sspB	498	3,873	1,558
JW3198	sspA	639	9,242	2,896
JW3199	rpsI	393	1,606	815
JW3200	rplM	429	2,328	1,088
JW3201	yhcM	1,128	6,186	1,098
JW3203	degQ	1,368	5,722	836
JW3204	degS	1,068	14,520	2,722
JW3205	mdh	939	3,876	832
JW3206	argR	471	2,318	984
JW3208	yhcO	273	169	123
JW3209	aaeB	1,968	4,040	410
JW3210	aaeA	933	8,008	1,718
JW3212	aaeR	930	3,840	822
JW3213	tldD	1,446	23,392	3,236
JW3216	rng	1,470	22,368	3,032
JW3217	yhdE	594	1,029	349
JW3218	mreD	489	1,858	765
JW3219	mreC	1,104	1,877	341
JW3220	mreB	1,044	6,440	1,234
JW3221	yhdA	1,941	7,305	752
JW3222	yhdH	975	14,260	2,922
JW3223	accB	471	2,789	1,176
JW3224	accC	1,350	6,981	1,033
JW3225	yhdT	243	1,671	1,368
JW3226	panF	1,452	10,574	1,458
JW3227	prmA	882	5,629	1,286
JW3228	dusB	966	12,820	2,656
JW3229	fis	297	281	190
JW3231	yhdU	180	117	132
JW3232	envR	663	5,443	1,641
JW3233	acrE	1,158	12,064	2,081
JW3234	acrF	3,105	3,770	244
JW3235	yhdV	222	657	587
JW3236	yhdW	918	1,862	405
JW3239	yhdZ	759	1,113	292
JW3241	yrdB	258	378	293
JW3242	aroE	819	1,118	274
JW3243	yrdC	573	2,301	806
JW3245	smg	474	241	102
JW3248	def	510	430	165
JW3249	fmt	948	1,877	395
JW3250	rsmB	1,290	5,856	909
JW3251	trkA	1,377	5,338	777
JW3252	mscL	411	1,339	647
JW3253	yhdL	219	1,018	927
JW3254	zntR	426	1,296	610
JW3255	yhdN	369	1	0
JW3256	rplQ	384	1,461	758
JW3257	rpoA	990	5,912	1,194
JW3258	rpsD	621	1,707	551
JW3259	rpsK	390	1,127	577
JW3260	rpsM	357	771	435
JW3261	rpmJ	117	59	97
JW3262	secY	1,332	3,135	468
JW3263	rplO	435	1,752	807
JW3264	rpmD	180	281	316
JW3265	rpsE	504	1,573	622

JW3266	rplR	354	2,007	1,119
JW3267	rplF	534	810	302
JW3268	rpsH	393	0	0
JW3269	rpsN	306	694	456
JW3270	rplE	540	1,101	407
JW3271	rplX	315	753	482
JW3272	rplN	372	883	477
JW3273	rpsQ	255	161	122
JW3274	rpmC	192	260	273
JW3275	rplP	411	2,282	1,107
JW3276	rpsC	702	5,711	1,632
JW3277	rplV	333	651	389
JW3278	rpsS	279	495	350
JW3279	rplB	822	4,510	1,100
JW3280	rplW	303	533	350
JW3281	rplD	606	781	258
JW3282	rplC	630	427	136
JW3283	rpsJ	312	1,208	781
JW3284	pioO	420	274	130
JW3285	gspA	1,470	2,802	380
JW3286	gspC	816	446	111
JW3288	gspE	1,482	7,879	1,067
JW3289	gspF	1,197	14,174	2,362
JW3290	gspG	438	1,440	656
JW3291	gspH	510	2,118	828
JW3293	gspJ	588	1,418	485
JW3294	gspK	984	6,808	1,380
JW3297	gspO	678	3,987	1,179
JW3298	bfr	477	199	82
JW3299	bfd	195	219	227
JW3300	chiA	2,694	4,865	362
JW3301	tufA	1,185	2,018	342
JW3302	fusA	2,115	10,296	976
JW3303	rpsG	540	3,276	1,207
JW3304	rpsL	375	4,829	2,562
JW3305	yheL	288	1,356	936
JW3306	yheM	360	2,411	1,335
JW3307	yheN	387	2,146	1,109
JW3309	fkpA	813	1,145	281
JW3310	slyX	219	188	169
JW3311	slyD	591	1,819	610
JW3312	yheV	201	326	326
JW3313	kefB	1,806	4,961	549
JW3314	kefG	555	741	265
JW3315	yheS	1,914	5,425	567
JW3316	yheT	1,023	9,652	1,881
JW3317	yheU	219	358	323
JW3318	prkB	870	3,164	728
JW3319	yhfA	405	2,209	1,084
JW3322	argD	1,221	10,397	1,696
JW3323	pabA	564	1,837	656
JW3324	fic	603	4,236	1,406
JW3325	yhfG	168	117	138
JW3326	ppiA	573	2,556	889
JW3327	tsgA	1,182	8,542	1,447
JW3328	nirB	2,544	2,201	174
JW3329	nirD	327	1,264	771
JW3330	nirC	807	2,010	500
JW3331	cysG	1,374	5,436	793
JW3332	yhfL	168	20	25
JW3333	frlA	1,338	3,043	451

JW3337	frlD	786	1,593	404
JW3339	yhfS	1,086	15,151	2,786
JW3340	yhfT	1,305	4,192	645
JW3342	php	879	4,048	923
JW3343	yhfW	1,227	5,817	950
JW3344	yhfX	1,164	1,654	286
JW3347	trpS	1,005	1,508	301
JW3348	gph	759	8,854	2,316
JW3349	rpe	678	4,429	1,299
JW3350	dam	837	1,013	240
JW3351	damX	1,287	2,048	317
JW3352	aroB	1,089	13,444	2,460
JW3354	hofQ	1,239	2,487	401
JW3356	yrfB	441	2,484	1,124
JW3357	yrfC	540	1,376	510
JW3359	mrcA	2,553	1,753	137
JW3360	nudE	561	954	341
JW3361	yrfF	2,136	2,649	249
JW3363	hslR	402	4,568	2,262
JW3365	yhgE	1,725	4,906	573
JW3366	pck	1,623	7,546	931
JW3367	envZ	1,353	5,521	817
JW3368	ompR	720	5,859	1,628
JW3369	greB	477	2,127	890
JW3370	yhgF	2,322	12,102	1,043
JW3371	feoA	228	544	476
JW3372	feoB	2,322	2,912	251
JW3373	yhgG	237	1,082	917
JW3374	yhgA	879	4,993	1,133
JW3375	bioH	771	1,247	324
JW3377	gntY	576	1,170	410
JW3379	malQ	2,085	34,978	3,355
JW3381	malT	2,706	11,127	822
JW3384	rtcB	1,227	1,375	224
JW3385	rtcR	1,599	15,262	1,910
JW3386	glpR	759	4,126	1,089
JW3388	glpE	327	1,752	1,070
JW3389	glpD	1,506	2,210	295
JW3390	yzgL	282	373	266
JW3391	glgP	2,448	5,364	440
JW3392	glgA	1,434	5,020	702
JW3393	glgC	1,296	4,215	652
JW3394	glgX	1,974	5,604	570
JW3395	glgB	2,187	3,569	326
JW3396	asd	1,104	8,435	1,531
JW3397	yhgN	594	156	53
JW3400	gntK	528	2,420	918
JW3402	yhhW	696	1,662	477
JW3403	yhhX	1,038	6,610	1,269
JW3405	yhhY	489	7,607	3,097
JW3406	yhhZ	1,179	384	66
JW3408	insA	276	9	7
JW3409	insB	504	20	7
JW3411	yrhB	285	167	119
JW3412	ggt	1,743	4,311	496
JW3413	yhhA	441	6,918	3,131
JW3414	ugpQ	744	4,372	1,172
JW3415	ugpC	1,071	13,879	2,593
JW3416	ugpE	846	3,367	790
JW3417	ugpA	888	8,535	1,912
JW3418	ugpB	1,317	2,553	388

JW3419	livF	714	6,005	1,673
JW3420	livG	768	5,846	1,515
JW3421	livM	1,278	3,701	578
JW3422	livH	927	4,253	912
JW3423	livK	1,110	7,068	1,268
JW3424	yhhK	384	1,336	697
JW3425	livJ	1,104	23,800	4,308
JW3426	rpoH	855	2,817	658
JW3427	ftsX	1,059	2,237	422
JW3428	ftsE	669	3,017	898
JW3429	ftsY	1,494	3,824	509
JW3430	yhhF	597	2,900	971
JW3432	yhhM	360	583	316
JW3433	yhhN	627	1,829	583
JW3434	zntA	2,199	2,677	244
JW3435	yhhP	246	391	315
JW3436	yhhQ	666	3,727	1,120
JW3440	acpT	588	2,705	914
JW3441	nikA	1,575	3,226	410
JW3442	nikB	945	12,729	2,686
JW3443	nikC	834	6,359	1,524
JW3444	nikD	765	6,950	1,815
JW3445	nikE	807	12,439	3,083
JW3446	nikR	402	1,483	731
JW3449	yhhH	384	200	103
JW3451	yhhI	1,137	1,138	202
JW3454	yhiI	1,068	2,879	542
JW3455	yhiJ	1,623	2,367	292
JW3457	yhiL	1,239	557	89
JW3459	yhiN	1,203	1,116	186
JW3460	pitA	1,500	4,422	587
JW3461	yhiO	336	965	581
JW3462	uspA	435	651	303
JW3463	yhiP	1,470	2,696	366
JW3465	prlC	2,043	9,713	952
JW3466	yhiR	843	5,528	1,306
JW3467	gor	1,353	8,444	1,246
JW3468	arsR	354	722	407
JW3469	arsB	1,290	15,216	2,343
JW3470	arsC	426	790	372
JW3471	yhiS	783	365	93
JW3472	insH	1,017	0	0
JW3474	slp	567	0	0
JW3475	yhiF	531	759	285
JW3478	hdeA	333	204	125
JW3479	hdeD	573	2,613	904
JW3480	gadE	528	222	85
JW3481	mdtE	1,158	7,528	1,301
JW3482	mdtF	3,114	9,781	627
JW3483	gadW	729	649	179
JW3484	gadX	825	1,173	285
JW3485	gadA	1,401	10,898	1,560
JW3486	yhjA	1,398	10,754	1,536
JW3487	treF	1,650	7,611	919
JW3488	yhjB	603	1,979	654
JW3489	yhjC	972	7,984	1,645
JW3490	yhjD	1,014	10,239	2,021
JW3491	yhjE	1,323	9,570	1,440
JW3492	yhjG	2,076	7,572	732
JW3493	yhjH	768	4,167	1,087
JW3495	yhjJ	1,497	21,163	2,829

JW3496	dctA	1,287	12,470	1,932
JW3499	bcsZ	1,107	7,883	1,429
JW3500	bcsB	2,340	3,698	316
JW3503	yhjR	189	454	479
JW3504	bcsE	1,572	13,234	1,684
JW3506	bcsG	1,680	7,531	899
JW3508	yhjV	1,272	3,942	619
JW3509	dppF	1,005	9,468	1,887
JW3510	dppD	984	5,237	1,063
JW3511	dppC	903	2,693	591
JW3512	dppB	1,020	13,910	2,725
JW3513	dppA	1,608	7,222	898
JW3516	yhjX	1,209	5,659	935
JW3518	tag	564	3,578	1,266
JW3519	yiaC	441	1,220	551
JW3524	yiaG	291	318	218
JW3525	cspA	213	154	146
JW3526	hokA	153	32	42
JW3527	insJ	522	0	0
JW3528	insK	852	0	0
JW3530	glyS	2,070	6,114	593
JW3531	glyQ	912	6,534	1,430
JW3532	ysaB	300	375	247
JW3533	yiaH	996	9,885	1,975
JW3534	yiaA	438	394	177
JW3536	xylB	1,455	2,049	283
JW3537	xylA	1,323	2,871	435
JW3538	xylF	993	1,727	348
JW3539	xylG	1,542	3,251	423
JW3540	xylH	1,182	12,664	2,126
JW3541	xylR	1,179	1,109	188
JW3543	malS	2,031	7,082	700
JW3545	yiaI	474	1,215	513
JW3546	yiaJ	849	2,708	637
JW3547	yiaK	999	4,543	908
JW3548	yiaL	468	2,685	1,152
JW3549	yiaM	474	810	340
JW3551	yiaO	987	6,939	1,409
JW3552	lyxK	1,497	9,349	1,249
JW3553	sgbH	663	7,752	2,331
JW3555	sgbE	696	4,811	1,375
JW3556	yiaT	741	4,928	1,330
JW3557	yiaU	975	6,437	1,317
JW3558	yiaV	1,137	6,856	1,210
JW3559	yiaW	324	324	202
JW3561	aldB	1,539	0	0
JW3563	selB	1,845	2,436	266
JW3564	selA	1,392	9,515	1,363
JW3565	yibF	609	5,842	1,907
JW3566	rhsA	4,134	2,265	109
JW3568	yibA	843	1,970	466
JW3570	yibG	462	92	40
JW3571	yibH	1,137	2,036	360
JW3572	yibI	363	380	210
JW3573	mtlA	1,914	4,371	459
JW3574	mtlD	1,149	4,191	729
JW3575	mtlR	588	4,434	1,503
JW3576	yibT	210	93	92
JW3577	yibL	363	873	479
JW3578	lldP	1,656	7,117	861
JW3579	lldR	777	4,830	1,244

JW3580	lldD	1,191	3,589	603
JW3581	yibK	474	7,553	3,174
JW3582	cysE	822	10,248	2,481
JW3583	gpsA	1,020	2,850	558
JW3584	secB	468	1,524	648
JW3585	grxC	252	359	286
JW3586	yibN	432	2,299	1,066
JW3587	gpmI	1,545	1,887	244
JW3590	yibD	1,035	1,089	211
JW3591	tdh	1,026	6,239	1,212
JW3592	kbl	1,197	9,011	1,504
JW3594	rfaD	933	9,116	1,951
JW3595	rfaF	1,047	4,790	916
JW3596	rfaC	960	11,844	2,463
JW3597	rfaL	1,260	1,279	205
JW3598	rfaK	1,074	154	31
JW3599	rfaZ	852	664	156
JW3600	rfaY	699	138	39
JW3601	rfaJ	1,017	61	12
JW3602	rfaI	1,020	193	37
JW3603	rfaB	1,110	179	33
JW3604	rfaS	936	354	75
JW3605	rfaP	798	2,016	505
JW3606	rfaG	1,125	327	58
JW3607	rfaQ	1,035	578	111
JW3608	kdtA	1,278	8,431	1,315
JW3609	coaD	480	6,047	2,506
JW3610	mutM	810	10,736	2,637
JW3611	rpmG	168	32	39
JW3612	rpmB	237	496	424
JW3615	dut	456	4,508	1,970
JW3617	pyrE	642	4,822	1,499
JW3618	rph	687	10,123	2,953
JW3619	yicC	864	4,238	981
JW3620	dinD	825	1,738	418
JW3621	yicG	618	6,481	2,090
JW3622	ligB	1,683	4,174	498
JW3623	gmk	624	4,655	1,479
JW3624	rpoZ	276	643	459
JW3625	spoT	2,109	23,674	2,243
JW3626	trmH	690	2,863	833
JW3627	recG	2,082	4,819	463
JW3628	gltS	1,206	4,103	682
JW3629	yicE	1,392	927	132
JW3630	yicH	1,710	5,078	598
JW3631	yicI	2,319	15,166	1,311
JW3633	setC	1,185	1,220	205
JW3634	yicL	924	1,638	355
JW3635	nlpA	819	3,749	921
JW3640	ade	1,767	10,735	1,215
JW3641	uhpT	1,392	4,355	624
JW3642	uhpC	1,320	2,739	415
JW3643	uhpB	1,503	1,012	134
JW3644	uhpA	591	2,761	937
JW3645	ilvN	291	406	279
JW3646	ilvB	1,689	7,553	895
JW3647	ivbL	99	49	102
JW3650	yidF	498	6,846	2,730
JW3651	yidG	363	1,051	579
JW3652	yidH	348	540	308
JW3653	yidI	450	1,130	502

JW3654	yidJ	1,494	1,464	194
JW3655	yidK	1,716	2,445	283
JW3656	yidL	924	4,326	931
JW3658	glvG	639	1,207	378
JW3659	glvB	486	512	210
JW3660	glvC	1,107	2,411	436
JW3661	yidP	717	12,625	3,526
JW3662	yidE	1,662	4,047	487
JW3663	ibpB	429	411	191
JW3664	ibpA	414	616	295
JW3670	dgoK	879	6,432	1,459
JW3674	yidA	813	4,287	1,054
JW3675	yidB	399	1,497	750
JW3677	recF	1,074	399	74
JW3678	dnaN	1,101	2,063	374
JW3679	dnaA	1,404	3,917	558
JW3680	rpmH	141	136	188
JW3681	rnpA	360	1,021	566
JW3682	yidD	258	2,536	1,955
JW3683	yidC	1,647	5,069	621
JW3684	trmE	1,365	7,322	1,071
JW3685	tnaC	75	9	26
JW3686	tnaA	1,416	5,711	804
JW3688	mdtL	1,176	4,496	766
JW3689	yidZ	960	19,192	4,004
JW3690	yieE	762	11,240	2,955
JW3691	yieF	567	2,112	744
JW3692	yieG	1,338	2,879	430
JW3693	yieH	666	497	153
JW3694	yieI	468	334	143
JW3695	cbrC	588	1,507	513
JW3698	bglH	1,617	840	104
JW3699	bglB	1,413	5,435	769
JW3700	bglF	1,878	9,028	959
JW3701	bglG	837	2,378	571
JW3702	phoU	726	8,037	2,214
JW3703	pstB	774	2,095	544
JW3704	pstA	891	2,103	473
JW3705	pstC	960	6,636	1,381
JW3706	pstS	1,041	2,712	521
JW3707	glmS	1,830	20,022	2,178
JW3708	glmU	1,371	3,322	483
JW3709	atpC	420	4,209	1,996
JW3710	atpD	1,383	6,182	893
JW3711	atpG	864	9,235	2,146
JW3712	atpA	1,542	14,446	1,873
JW3713	atpH	534	4,647	1,742
JW3714	atpF	471	3,558	1,521
JW3715	atpE	240	992	828
JW3716	atpB	816	3,921	961
JW3718	gidB	624	3,191	1,025
JW3719	gidA	1,890	5,121	543
JW3720	mioC	444	2,320	1,041
JW3721	asnC	459	3,528	1,532
JW3722	asnA	993	23,362	4,706
JW3725	yieN	1,497	1,733	231
JW3728	rbsA	1,506	18,793	2,502
JW3729	rbsC	966	11,529	2,388
JW3730	rbsB	891	3,435	766
JW3731	rbsK	930	2,697	580
JW3732	rbsR	993	12,857	2,594

JW3733	hsrA	1,428	5,918	828
JW3737	yifE	339	350	208
JW3738	yifB	1,551	8,046	1,039
JW3739	ilvL	99	39	76
JW3740	ilvG	984	5,988	1,221
JW3741	ilvG	582	12,596	4,318
JW3742	ilvM	264	218	160
JW3745	ilvA	1,545	6,825	884
JW3746	ilvY	894	4,781	1,067
JW3747	ilvC	1,476	8,735	1,179
JW3748	ppiC	282	217	155
JW3749	yifO	276	441	322
JW3750	yifN	219	100	89
JW3753	rhlB	1,266	5,354	845
JW3755	rhoL	102	0	0
JW3756	rho	1,260	12,567	2,001
JW3758	rfe	1,104	6,494	1,168
JW3763	rffH	882	8,575	1,945
JW3765	rffA	1,131	3,913	695
JW3766	wzxE	1,251	3,973	633
JW3769	wzyE	1,353	19,763	2,926
JW3770	rffM	741	6,662	1,782
JW3773	aslA	1,656	2,357	286
JW3774	hemY	1,197	4,267	712
JW3775	hemX	1,182	8,606	1,452
JW3776	hemD	741	5,637	1,522
JW3778	cyaA	2,547	1,843	145
JW3779	cyaY	321	1,312	818
JW3780	yzcX	486	4,755	1,950
JW3781	yifL	204	365	360
JW3783	yigA	708	14,607	4,134
JW3784	xerC	897	10,074	2,238
JW3785	yigB	717	8,836	2,457
JW3786	uvrD	2,163	403	37
JW3789	corA	951	6,528	1,376
JW3790	yigF	381	301	158
JW3794	pldA	870	13,444	3,090
JW3803	yigM	900	18,474	4,082
JW3804	metR	954	12,280	2,565
JW3805	metE	2,262	9,073	799
JW3808	udp	762	22,891	6,002
JW3809	rmuC	1,428	7,257	1,013
JW3811	yigP	606	27,237	8,930
JW3812	ubiB	1,641	5,690	695
JW3813	tatA	270	500	371
JW3815	tatC	777	6,145	1,568
JW3818	rfaH	489	16,393	6,698
JW3819	ubiD	1,494	17,041	2,281
JW3820	fre	702	5,905	1,676
JW3822	fadB	2,190	9,618	878
JW3823	pepQ	1,332	15,347	2,309
JW3827	hemG	546	6,501	2,375
JW3829	mobA	585	4,229	1,443
JW3830	yihD	270	398	295
JW3831	yihE	987	4,745	956
JW3832	dsbA	627	3,227	1,039
JW3834	yihG	933	743	160
JW3835	polA	2,787	3,038	219
JW3837	yihI	510	5,423	2,122
JW3838	hemN	1,374	2,876	422
JW3839	glnG	1,410	1,516	216

JW3840	glnL	1,050	7,814	1,493
JW3841	glnA	1,410	13,136	1,862
JW3843	yihL	711	2,924	824
JW3844	yihM	981	2,138	435
JW3845	yihN	1,266	5,941	937
JW3846	ompL	693	1,226	355
JW3848	yihP	1,407	1,696	241
JW3849	yihQ	2,037	5,718	560
JW3850	yihR	927	6,398	1,376
JW3852	yihT	879	11,283	2,563
JW3853	yihU	897	43,594	9,685
JW3857	rbn	873	5,009	1,150
JW3858	dtd	438	6,699	3,043
JW3859	yiiD	990	731	148
JW3862	fdhE	930	3,515	751
JW3863	fdoI	636	4,461	1,397
JW3864	fdoH	903	4,799	1,068
JW3865	fdoG	3,051	3,868	255
JW3866	fdhD	834	4,854	1,165
JW3867	yiiG	1,056	747	141
JW3868	frvR	1,749	5,411	619
JW3869	frvX	1,071	3,568	667
JW3871	frvA	447	2,287	1,021
JW3872	yiiL	315	0	0
JW3873	rhaD	825	8,451	2,047
JW3875	rhaB	1,470	26,195	3,555
JW3876	rhaS	837	6,169	1,469
JW3877	rhaR	939	19,497	4,151
JW3878	rhaT	1,035	2,124	409
JW3879	sodA	621	2,285	735
JW3882	cpxA	1,374	2,490	364
JW3883	cpxR	699	6,646	1,897
JW3886	fieF	903	4,459	982
JW3887	pfkA	963	9,084	1,889
JW3888	sbp	990	447	92
JW3889	cdh	756	4,670	1,233
JW3890	tpiA	768	3,954	1,030
JW3891	yiiQ	600	3,032	1,015
JW3892	yiiR	441	3,965	1,794
JW3893	yiiS	300	315	209
JW3894	yiiT	429	353	166
JW3895	fpr	747	2,104	564
JW3896	glpX	1,011	5,435	1,073
JW3897	glpK	1,509	4,032	533
JW3898	glpF	846	1,871	442
JW3899	yiiU	246	443	359
JW3900	rraA	486	3,932	1,613
JW3901	menA	927	2,581	555
JW3902	hslU	1,332	2,385	360
JW3903	hslV	531	2,507	944
JW3904	ftsN	960	8,505	1,778
JW3905	cytR	1,026	2,673	522
JW3906	priA	2,199	1,150	105
JW3907	rpmE	213	1,192	1,111
JW3908	yiiX	609	4,538	1,487
JW3909	metJ	318	3,929	2,450
JW3910	metB	1,161	607	106
JW3911	metL	2,433	3,212	264
JW3913	metF	891	4,392	988
JW3914	katG	2,181	17,595	1,611
JW3916	yijF	618	404	132

JW3918	fsaB	663	2,695	807
JW3921	frwC	1,080	3,439	634
JW3922	frwB	321	1,047	650
JW3923	pflD	2,298	15,392	1,345
JW3924	pflC	879	2,852	646
JW3925	frwD	342	2,189	1,274
JW3926	yijO	852	5,393	1,266
JW3927	yijP	1,734	4,402	512
JW3928	ppc	2,652	3,183	241
JW3929	argE	1,152	1,120	195
JW3930	argC	1,005	1,868	375
JW3932	argH	1,374	1,249	181
JW3933	oxyR	918	11,457	2,498
JW3935	fabR	705	4,506	1,283
JW3936	yijD	360	1,078	596
JW3937	trmA	1,101	8,016	1,462
JW3938	btuB	1,845	10,023	1,086
JW3940	murB	1,029	253	49
JW3941	birA	966	11,550	2,390
JW3942	coaA	951	939	199
JW3943	tufB	1,185	1,681	286
JW3944	secE	384	5,203	2,687
JW3945	nusG	546	2,019	737
JW3946	rplK	429	2,754	1,273
JW3947	rplA	705	2,781	790
JW3948	rplJ	498	1,420	572
JW3949	rplL	366	182	98
JW3950	rpoB	4,029	3,690	184
JW3951	rpoC	4,224	17,837	847
JW3952	htrC	540	842	313
JW3953	thiH	1,134	4,170	735
JW3955	thiS	201	28	28
JW3956	thiF	756	6,528	1,725
JW3957	thiE	636	4,222	1,316
JW3958	thiC	1,896	5,591	590
JW3959	rsd	477	7,068	2,952
JW3961	hemE	1,065	2,009	376
JW3963	yjaG	591	2,060	700
JW3964	hupA	273	977	714
JW3965	yjaH	696	4,585	1,317
JW3967	zraS	1,398	1,597	228
JW3968	zraR	1,326	1,143	171
JW3969	purD	1,290	5,499	853
JW3970	purH	1,590	3,938	499
JW3971	yjaA	384	139	73
JW3972	yjaB	444	1,958	880
JW3973	metA	930	259	56
JW3974	aceB	1,602	7,219	903
JW3975	aceA	1,305	9,419	1,446
JW3976	aceK	1,737	6,692	770
JW3977	arpA	2,187	2,823	258
JW3978	iclR	825	5,161	1,250
JW3979	metH	3,684	9,287	505
JW3980	yjbB	1,632	5,753	705
JW3981	pepE	690	7,795	2,248
JW3982	yjbC	873	1,760	404
JW3983	yjbD	273	1,428	1,042
JW3984	lysC	1,350	2,850	420
JW3985	pgi	1,650	5,782	700
JW3986	yjbE	243	299	242
JW3988	yjbG	738	2,138	574

JW3989	yjbH	2,097	7,477	712
JW3990	yjbA	411	5,085	2,467
JW3991	xylE	1,476	1,142	154
JW3992	malG	891	9,594	2,147
JW3993	malF	1,545	3,239	423
JW3994	malE	1,191	2,826	476
JW3995	malK	1,116	3,825	686
JW3996	lamB	1,341	820	122
JW3997	malM	921	5,370	1,167
JW3998	yjbI	1,329	413	62
JW4000	ubiA	873	21,364	4,875
JW4001	plsB	2,424	14,297	1,185
JW4002	dgkA	369	2,698	1,460
JW4003	lexA	609	9,238	3,024
JW4004	dinF	1,380	11,146	1,610
JW4005	yjbJ	210	21	20
JW4007	yjbL	255	91	70
JW4008	yjbM	708	905	257
JW4011	qor	984	5,011	1,018
JW4012	dnaB	1,416	2,890	408
JW4013	alr	1,080	1,154	213
JW4014	tyrB	1,194	1,863	313
JW4015	aphA	714	5,452	1,526
JW4017	yjbQ	417	1,056	506
JW4018	yjbR	357	2,131	1,192
JW4019	uvrA	2,823	26,485	1,879
JW4020	ssb	537	8,107	3,016
JW4022	yjcC	1,587	358	45
JW4023	soxS	324	1,269	785
JW4024	soxR	465	4,938	2,116
JW4025	yjcD	1,350	5,151	766
JW4026	yjcE	1,650	4,335	525
JW4027	yjcF	1,293	987	152
JW4028	actP	1,650	3,696	448
JW4029	yjcH	315	1,084	682
JW4030	acs	1,959	8,126	831
JW4031	nrfA	1,437	3,185	444
JW4032	nrfB	567	4,757	1,675
JW4033	nrfC	672	8,645	2,566
JW4034	nrfD	957	9,758	2,034
JW4035	nrfE	1,659	2,108	254
JW4036	nrfF	384	1,124	587
JW4037	nrfG	597	19,867	6,622
JW4038	gltP	1,314	4,253	644
JW4039	yjcO	690	11,597	3,346
JW4040	fdhF	2,148	28,542	2,659
JW4041	yjcP	1,467	3,354	459
JW4042	yjcQ	2,052	3,987	391
JW4043	yjcR	1,032	553	108
JW4046	alsE	696	4,269	1,227
JW4047	alsC	981	1,279	262
JW4048	alsA	1,533	1,155	150
JW4049	alsB	936	4,858	1,038
JW4050	rpiR	891	5,941	1,338
JW4051	rpiB	450	2,717	1,202
JW4053	phnP	759	645	169
JW4054	phnO	435	1,649	759
JW4055	phnN	558	31,122	11,132
JW4056	phnM	1,137	5,087	896
JW4057	phnL	681	9,050	2,655
JW4059	phnJ	846	0	0

JW4060	phnI	1,065	1,974	370
JW4061	phnH	585	4,447	1,522
JW4062	phnG	453	3,543	1,543
JW4063	phnF	726	9,035	2,481
JW4064	phnE	369	1,990	1,082
JW4065	phnE	621	6,159	1,973
JW4066	phnD	1,017	2,285	451
JW4067	phnC	789	5,934	1,501
JW4068	phnB	444	4,025	1,795
JW4069	phnA	336	483	289
JW4070	yjdA	2,229	8,528	766
JW4072	proP	1,503	2,595	344
JW4073	basS	1,092	2,612	477
JW4074	basR	669	6,905	2,052
JW4076	adiC	1,338	656	99
JW4077	adiY	762	4,098	1,075
JW4079	melR	909	1,950	434
JW4080	melA	1,356	2,457	365
JW4081	melB	1,410	1,771	250
JW4082	yjdF	630	5,170	1,639
JW4083	fumB	1,647	6,233	757
JW4084	dcuB	1,341	3,946	590
JW4085	dcuR	720	11,750	3,244
JW4086	dcuS	1,632	5,730	703
JW4087	yjdI	231	397	344
JW4088	yjdJ	273	165	121
JW4089	yjdK	297	54	36
JW4090	lysU	1,518	2,435	321
JW4091	yjdL	1,458	6,923	950
JW4092	cadA	2,148	1,768	166
JW4093	cadB	1,335	2,614	393
JW4094	cadC	1,539	1,145	149
JW4097	cutA	339	889	520
JW4099	aspA	1,437	4,054	565
JW4100	fxsA	477	2,118	891
JW4101	yjeH	1,257	6,396	1,016
JW4102	groS	294	1,538	1,046
JW4103	groL	1,647	3,439	417
JW4105	yjeJ	870	1,419	327
JW4106	yjeK	1,029	5,516	1,074
JW4107	efp	567	2,314	817
JW4108	ecnB	147	177	234
JW4110	blc	534	6,538	2,447
JW4111	ampC	1,134	4,802	845
JW4112	frdD	360	5,468	3,029
JW4113	frdC	396	1,550	788
JW4114	frdB	735	6,841	1,846
JW4115	frdA	1,809	5,074	560
JW4116	poxA	978	21,117	4,303
JW4118	yjeN	315	1,807	1,149
JW4119	yjeO	315	586	371
JW4120	yjeP	3,324	6,535	395
JW4121	psd	969	8,571	1,769
JW4122	rsgA	1,053	13,925	2,647
JW4124	yjeS	1,140	1,789	317
JW4125	yjeF	1,548	4,842	626
JW4126	yjeE	462	2,541	1,103
JW4127	amiB	1,338	1,082	163
JW4128	mutL	1,848	3,006	325
JW4129	miaA	951	1,404	290
JW4130	hfq	309	1,506	974

JW4131	hflX	1,281	6,184	962
JW4132	hflK	1,260	6,124	976
JW4133	hflC	1,005	2,018	401
JW4134	yjeT	198	233	229
JW4135	purA	1,299	17,843	2,739
JW4136	yjeB	426	1,965	919
JW4138	rlmB	732	5,326	1,446
JW4139	yjfI	402	2,195	1,090
JW4140	yjfJ	699	1,264	362
JW4141	yjfK	660	1,708	523
JW4142	yjfL	399	368	188
JW4143	yjfM	639	598	185
JW4144	yjfC	1,164	4,270	733
JW4148	yjfP	750	4,757	1,265
JW4149	ulaR	756	3,245	856
JW4152	ulaB	306	953	625
JW4153	ulaC	465	2,641	1,140
JW4154	ulaD	651	5,682	1,741
JW4155	ulaE	855	4,825	1,126
JW4156	ulaF	687	14,640	4,250
JW4157	yjfY	276	216	160
JW4158	rpsF	396	1,522	772
JW4159	priB	315	1,615	1,023
JW4160	rpsR	228	20	18
JW4161	rplI	450	2,017	891
JW4162	yjfZ	795	0	0
JW4163	ytfA	327	205	123
JW4166	cycA	1,413	4,839	685
JW4167	ytfE	663	3,512	1,061
JW4168	ytfF	975	12,931	2,650
JW4169	ytfG	861	3,271	761
JW4171	cpdB	1,944	8,150	840
JW4172	cysQ	741	0	0
JW4175	ytfJ	555	10,989	3,944
JW4177	ytfL	1,344	5,112	762
JW4178	msrA	639	5,931	1,857
JW4179	ytfM	1,734	11,535	1,333
JW4180	ytfN	3,780	2,189	116
JW4181	ytfP	342	3,273	1,901
JW4182	yzfA	270	2,561	1,893
JW4184	chpB	351	1,944	1,109
JW4185	ppa	531	4,320	1,620
JW4186	ytfQ	957	4,007	836
JW4191	fbp	999	4,471	892
JW4192	mpl	1,374	5,339	777
JW4193	yjgA	552	2,490	904
JW4194	pmbA	1,353	9,707	1,432
JW4195	cybC	303	2,577	1,698
JW4196	nrdG	465	4,683	2,017
JW4197	nrdD	2,139	6,791	635
JW4198	treC	1,656	15,176	1,832
JW4199	treB	1,422	11,164	1,570
JW4200	treR	948	3,099	653
JW4201	mgtA	2,697	3,538	262
JW4203	pyrI	462	1,753	760
JW4204	pyrB	936	2,960	631
JW4205	pyrL	135	445	661
JW4206	yjgH	396	66	35
JW4207	yjgI	714	1,584	443
JW4208	yjgJ	594	1,203	403
JW4211	argI	1,005	24,534	4,881

JW4212	yjgD	417	504	245
JW4215	valS	2,856	4,208	293
JW4216	holC	444	872	390
JW4217	pepA	1,512	4,048	534
JW4218	yjgP	1,101	2,286	416
JW4220	yjgR	1,503	5,615	745
JW4221	idnR	999	3,040	606
JW4222	idnT	1,320	5,156	782
JW4223	idnO	765	6,699	1,747
JW4224	idnD	1,032	4,364	847
JW4225	idnK	564	421	151
JW4227	intB	1,191	750	126
JW4229	insC	411	0	0
JW4230	insD	906	21	4
JW4233	yjgW	336	414	249
JW4234	yjgX	447	215	97
JW4236	yjgZ	330	849	513
JW4242	yjhE	249	1,025	812
JW4244	insI	1,152	14	2
JW4245	insO	597	0	0
JW4246	yjhV	414	542	261
JW4247	fecE	768	3,642	948
JW4248	fecD	957	2,864	600
JW4249	fecC	999	451	89
JW4250	fecB	903	8,398	1,857
JW4251	fecA	2,325	3,306	284
JW4252	fecR	954	6,005	1,263
JW4253	fecI	522	918	349
JW4254	insA	276	0	0
JW4255	insB	294	0	0
JW4256	insB	210	1	1
JW4258	yjhF	1,350	8,120	1,197
JW4259	yjhG	1,968	5,884	595
JW4261	yjhI	789	406	102
JW4262	sgcR	783	1,739	444
JW4263	sgcE	633	3,841	1,212
JW4264	sgcA	432	1,597	741
JW4265	sgcQ	807	7,355	1,818
JW4266	sgcC	1,314	10,118	1,547
JW4268	yjhP	747	1,212	325
JW4269	yjhQ	546	4,681	1,706
JW4271	yjhR	1,017	606	119
JW4272	yjhS	981	4,669	952
JW4275	fimB	603	728	240
JW4276	fimE	597	1,186	396
JW4277	fimA	549	1,770	640
JW4279	fimC	726	866	237
JW4281	fimF	531	1,362	515
JW4282	fimG	504	1,184	474
JW4283	fimH	903	9,695	2,145
JW4284	gntP	1,344	16,180	2,396
JW4285	uxuA	1,185	5,278	894
JW4286	uxuB	1,461	8,898	1,220
JW4287	uxuR	774	3,030	779
JW4288	yjiC	831	269	64
JW4290	yjiE	912	623	137
JW4291	iadA	1,173	5,872	1,004
JW4292	yjiG	462	2,386	1,033
JW4295	yjiJ	1,179	6,383	1,082
JW4299	yjiN	1,281	4,909	766
JW4300	yjiO	1,233	10,518	1,707

JW4302	yjiQ	561	1,195	426
JW4303	yjiR	1,413	14,472	2,048
JW4304	yjiS	165	525	635
JW4310	yjiW	399	195	97
JW4311	hsdS	1,395	1,229	177
JW4312	hsdM	1,590	17,245	2,172
JW4313	hsdR	3,567	1,736	97
JW4314	mrr	915	5,094	1,117
JW4316	yjiX	204	121	120
JW4318	tsr	1,656	13,073	1,581
JW4319	yjiZ	1,362	8,871	1,303
JW4325	dnaC	738	3,067	831
JW4326	dnaT	540	2,660	984
JW4327	yjjB	327	134	81
JW4329	yjjQ	726	1,036	284
JW4331	fhuF	789	10,762	2,726
JW4333	rsmC	1,032	2,940	565
JW4334	holD	414	6,818	3,281
JW4335	rimI	447	3,343	1,490
JW4336	yjjG	678	4,770	1,408
JW4338	osmY	606	6,067	2,001
JW4340	yjjU	1,074	1,068	200
JW4341	yjjV	780	15,220	3,886
JW4342	yjjW	864	8,676	2,011
JW4343	yjjI	1,551	6,063	783
JW4344	deoC	780	4,340	1,114
JW4345	deoA	1,323	5,631	854
JW4346	deoB	1,224	2,924	481
JW4347	deoD	720	6,120	1,698
JW4348	yjjJ	1,332	8,820	1,318
JW4349	lplA	1,017	3,425	674
JW4350	ytjB	645	3,167	980
JW4351	serB	969	3,334	689
JW4352	radA	1,383	7,250	1,047
JW4354	yjjK	1,668	4,269	511
JW4355	slt	1,938	5,692	587
JW4356	trpR	327	3,696	2,253
JW4358	ytjC	648	4,248	1,315
JW4359	rob	870	12,268	2,819
JW4360	creA	474	2,613	1,101
JW4361	creB	690	10,692	3,068
JW4362	creC	1,425	6,068	852
JW4363	creD	1,353	6,113	903
JW4364	arcA	717	2,031	562
JW4365	yjjY	141	66	98
JW4366	yjtD	687	5,220	1,521
JW4367	thrL	66	1	3
JW5001	htgA	486	1,585	656
JW5002	hokC	153	33	43
JW5003	yaaY	219	474	432
JW5004	caiE	591	10,520	3,545
JW5005	yabI	765	1,205	316
JW5007	secM	513	2,287	893
JW5008	yacG	198	382	386
JW5009	hpt	537	1,050	396
JW5010	yadD	903	34	8
JW5011	ligT	531	2,418	902
JW5012	clcA	1,422	1,291	182
JW5013	cdaR	1,158	19,800	3,416
JW5014	yaeI	813	5,942	1,459
JW5016	yaeF	825	2,276	549

JW5017	yafD	801	6,295	1,569
JW5018	mltD	1,359	4,992	731
JW5019	yafV	771	5,536	1,433
JW5020	fadE	2,445	4,258	350
JW5022	yafX	459	4,440	1,927
JW5023	ykfF	240	1,320	1,092
JW5024	insN	405	0	0
JW5027	mmuP	1,404	982	138
JW5030	yagV	711	2,791	784
JW5031	ykgK	591	456	155
JW5033	ykgL	228	381	334
JW5034	ykgM	141	140	199
JW5035	ykgM	264	57	43
JW5036	insE	309	113	75
JW5037	ykgA	687	637	187
JW5038	ykgB	594	744	249
JW5039	ykgI	237	161	137
JW5040	ykgC	1,326	4,202	633
JW5041	ykgE	720	1,481	413
JW5042	ykgG	696	7,281	2,082
JW5044	yahM	246	80	67
JW5046	mhpT	1,212	9,670	1,596
JW5049	ykiB	219	22	19
JW5050	insE	309	127	83
JW5051	yaiU	1,404	7,004	1,003
JW5052	ampH	1,158	11,243	1,941
JW5053	yaiZ	213	346	326
JW5054	psiF	321	2,763	1,723
JW5055	proY	1,374	7,027	1,021
JW5056	yajI	540	693	257
JW5057	yajL	591	2,471	838
JW5058	yajQ	492	3,358	1,371
JW5059	yajR	1,365	11,793	1,734
JW5060	bolA	318	142	92
JW5061	mdlB	1,782	21,432	2,398
JW5062	ylaB	1,551	3,334	431
JW5063	ylaC	471	689	292
JW5065	ybbJ	456	1,617	709
JW5066	ybbM	780	16	4
JW5067	ybbN	855	4,705	1,103
JW5070	ybcJ	213	1,562	1,456
JW5071	sfmH	978	11,609	2,368
JW5072	sfmF	516	2,933	1,129
JW5073	fimZ	633	476	152
JW5074	insE	309	126	82
JW5076	ylcG	141	52	74
JW5078	nmpC	1,128	973	174
JW5079	rzpD	462	3,442	1,491
JW5080	rzoD	183	996	1,089
JW5081	ybcV	411	200	98
JW5082	cusS	1,443	3,438	478
JW5083	ybdF	369	127	70
JW5084	hokE	153	20	27
JW5085	entD	630	1,538	489
JW5086	fepA	2,241	1,895	169
JW5087	citF	1,533	3,947	514
JW5089	lipB	642	2,278	707
JW5090	ybeB	318	213	135
JW5091	ybeQ	978	4,058	830
JW5092	gltI	909	3,263	711
JW5094	ybfG	411	3,287	1,587

JW5095	ybfH	210	61	59
JW5096	kdpE	678	5,165	1,517
JW5097	abrB	1,047	16	3
JW5098	ybgO	1,062	166	31
JW5099	ybgQ	2,448	8,554	698
JW5100	tolB	1,293	17,072	2,648
JW5102	ybhT	150	30	40
JW5103	ybhJ	2,262	10,723	954
JW5104	ybhF	1,737	4,140	479
JW5105	ybiX	678	4,398	1,295
JW5106	ybiM	261	994	758
JW5107	ybiN	927	3,291	706
JW5108	ybiO	2,226	18,176	1,634
JW5109	fsaA	663	8,135	2,450
JW5111	yliB	1,539	2,666	347
JW5112	ybjG	597	4,978	1,666
JW5113	ybjI	816	2,901	710
JW5114	ybjK	537	2,322	871
JW5116	ybjT	1,431	536	76
JW5117	hcr	969	5,452	1,128
JW5118	dmsA	2,445	6,281	514
JW5119	ycaM	1,431	6,056	846
JW5120	ycaI	2,265	5,526	488
JW5121	ssuC	792	6,267	1,575
JW5122	ycbQ	540	395	144
JW5123	ycbV	516	2,140	834
JW5124	ycbF	711	2,165	608
JW5125	ycbW	543	373	139
JW5126	ycbX	1,110	697	124
JW5127	ymbA	549	3,028	1,100
JW5128	yccS	2,154	12,625	1,174
JW5129	mgsA	459	2,417	1,054
JW5130	yccU	414	625	302
JW5131	yccX	279	1,512	1,073
JW5132	etp	447	1,798	800
JW5133	ymcD	306	2,179	1,424
JW5134	cspH	213	527	492
JW5135	torS	2,745	1,998	146
JW5136	ymdF	174	886	1,015
JW5137	ycdG	1,329	2,213	330
JW5138	ycdH	495	10,938	4,404
JW5139	ycdL	693	3,988	1,144
JW5141	ycdN	111	17	32
JW5142	ycdR	2,019	2,367	234
JW5143	ycdT	1,359	0	0
JW5144	insE	309	111	74
JW5145	ymdE	303	100	66
JW5146	ycdW	939	9,704	2,063
JW5147	ycdZ	492	6,150	2,487
JW5150	ymdC	1,422	2,471	348
JW5151	yceK	228	1,440	1,264
JW5152	yceP	255	256	202
JW5153	flgH	699	7,829	2,239
JW5154	yceQ	321	86	53
JW5155	yceF	585	4,447	1,513
JW5156	plsX	1,071	2,006	375
JW5157	ycfM	642	1,487	464
JW5158	ycfP	543	1,129	413
JW5159	ycfQ	633	7,247	2,284
JW5161	lolC	1,200	7,110	1,179
JW5162	lolD	702	5,704	1,623

JW5164	ymfA	462	851	370
JW5165	hflD	642	9,809	3,051
JW5166	ymfE	705	358	103
JW5168	ymfI	342	677	399
JW5169	ymfT	201	54	53
JW5170	ymfP	663	17,241	5,195
JW5171	ymfS	414	403	195
JW5172	stfE	537	1,039	383
JW5173	icdC	153	136	185
JW5174	ycgG	1,524	261	34
JW5176	ycgH	1,017	3,774	740
JW5177	ymgD	330	1,085	655
JW5178	ymgG	276	1,738	1,270
JW5179	ymgH	222	328	298
JW5180	ycgN	447	798	362
JW5181	hlyE	912	2,182	477
JW5182	dsbB	531	2,227	840
JW5184	cvrA	1,737	7,047	811
JW5185	dhaH	1,419	385	55
JW5186	dhaL	633	8,887	2,799
JW5187	dhaK	1,071	27,000	5,038
JW5188	dhaR	1,920	11,634	1,211
JW5189	ychM	1,680	1,558	185
JW5195	tonB	720	6,352	1,760
JW5196	yciO	621	2,278	736
JW5197	yciQ	1,896	129	14
JW5198	yciX	129	120	185
JW5199	yciX	189	74	77
JW5200	yciW	1,128	149	26
JW5201	puuA	1,419	1,396	196
JW5202	ycjR	789	3,434	870
JW5203	ymjB	114	39	69
JW5205	abgA	1,311	8,619	1,318
JW5206	ydaM	1,233	2,398	386
JW5207	ydaQ	216	52	47
JW5208	lar	195	121	121
JW5209	sieB	489	295	121
JW5210	ydaG	135	89	131
JW5211	ydaW	561	4,336	1,531
JW5212	rzpR	300	2,268	1,518
JW5213	rzoR	186	1,011	1,072
JW5215	ydbJ	267	572	430
JW5216	ydbL	327	387	233
JW5217	paaD	498	11,741	4,714
JW5218	paaK	1,314	0	0
JW5221	ydbD	2,307	4,743	411
JW5224	cybB	531	2,166	812
JW5225	hokB	150	164	216
JW5226	ydcI	924	2,517	545
JW5227	yncK	168	373	441
JW5228	ydcM	1,209	2,331	381
JW5229	ydcO	1,176	0	0
JW5230	yncN	177	315	358
JW5232	ydcX	174	264	296
JW5233	yncA	519	1,378	530
JW5234	ansP	1,500	14,693	1,955
JW5235	yncH	213	32	32
JW5237	yncM	201	39	38
JW5238	sfcA	1,698	1,029	121
JW5239	bdm	216	248	230
JW5240	ddpA	1,551	351	45

JW5241	yddV	1,044	377	72
JW5242	yddA	1,686	999	119
JW5243	ydeN	1,683	1,268	152
JW5244	yneL	180	115	127
JW5245	yneE	915	5,738	1,250
JW5247	yneI	1,389	3,826	552
JW5248	marR	435	2,780	1,274
JW5249	marA	384	308	161
JW5250	eamA	900	537	119
JW5251	ynfO	234	234	198
JW5252	ydfO	411	179	91
JW5253	gnsB	174	19	22
JW5254	ynfN	156	58	74
JW5255	essQ	216	134	125
JW5257	ynfP	111	0	0
JW5258	ynfC	711	0	0
JW5259	ynfD	306	1,341	869
JW5260	ynfF	2,424	9,150	756
JW5261	ynfH	855	3,323	780
JW5262	dmsD	615	5,435	1,766
JW5263	clcB	1,257	1,241	196
JW5264	ynfK	696	5,258	1,507
JW5265	ydgJ	1,041	14,304	2,752
JW5267	slyA	435	1,820	842
JW5270	ydhO	816	1,484	365
JW5271	ydhX	669	651	192
JW5272	ydhV	2,103	422	40
JW5273	sufB	1,488	1,731	233
JW5274	ydiN	1,266	2,072	327
JW5275	ydiO	1,152	2,026	353
JW5276	ydiQ	765	2,457	645
JW5277	pheS	984	10,050	2,040
JW5278	arpB	474	100	42
JW5280	pfkB	930	4,488	965
JW5281	ydjM	591	2,120	716
JW5282	astD	1,479	11,072	1,494
JW5283	ydjY	678	2,022	590
JW5284	ynjB	1,167	2,973	509
JW5285	ynjC	1,491	6,405	862
JW5286	ynjD	654	8,040	2,455
JW5287	ynjE	1,308	13,421	2,046
JW5288	ynjI	1,041	589	114
JW5289	ydjH	948	3,369	708
JW5290	ydjK	1,380	17,657	2,557
JW5291	yeaJ	1,491	498	67
JW5292	yeaP	1,026	2,472	482
JW5293	yeaV	1,446	4,581	634
JW5294	yeaW	1,125	0	0
JW5295	yoaB	345	6,518	3,759
JW5296	yoaC	360	259	145
JW5298	yobH	240	99	83
JW5299	yebQ	1,374	10,674	1,554
JW5300	proQ	699	3,639	1,041
JW5301	yebU	1,440	2,333	324
JW5302	yebV	237	81	69
JW5303	yebW	192	201	212
JW5304	yebA	1,323	3,269	493
JW5306	yebB	603	2,282	757
JW5307	yecD	567	7,338	2,586
JW5308	yecN	396	973	492
JW5309	yecM	567	1,312	464

JW5310	yecT	489	208	85
JW5312	otsA	1,425	4,232	594
JW5313	yedO	987	2,790	561
JW5316	fliO	366	3,927	2,150
JW5317	yodD	228	721	626
JW5319	yedS	486	2,563	1,054
JW5322	yedW	672	455	136
JW5323	yodB	531	455	172
JW5325	yeeL	327	348	215
JW5326	yoeA	393	692	351
JW5327	yeeP	426	10,930	5,115
JW5328	yoeF	357	7,340	4,097
JW5329	dacD	1,167	8,406	1,441
JW5330	yeeF	1,359	8,241	1,208
JW5331	yoeB	255	11	8
JW5335	nudD	480	4,513	1,873
JW5336	yegH	1,584	508	64
JW5338	mdtA	1,248	9,386	1,503
JW5339	yegP	333	1,479	881
JW5340	gatR	447	801	359
JW5341	insE	309	123	78
JW5343	gatY	855	4,295	1,005
JW5344	fbaB	1,053	11,930	2,264
JW5345	yegX	819	1,300	319
JW5346	yohN	339	7,762	4,561
JW5349	yehL	1,089	7,279	1,332
JW5350	yehP	1,137	3,315	581
JW5351	yehR	462	543	234
JW5352	yehT	720	2,595	719
JW5353	yehU	1,686	1,120	132
JW5354	yohO	108	136	250
JW5355	pbpG	933	782	167
JW5356	yohC	588	3,589	1,222
JW5358	yohH	138	0	0
JW5359	yeiS	240	169	140
JW5361	yeiW	255	4,551	3,562
JW5362	yeiP	573	2,430	844
JW5363	bcr	1,191	2,011	336
JW5366	ccmA	624	2,416	770
JW5367	napB	450	0	0
JW5368	yojL	1,056	6,554	1,243
JW5371	yfaZ	543	2	1
JW5372	yfbE	1,158	6,320	1,093
JW5373	yfbJ	387	354	184
JW5374	menD	1,671	7,395	888
JW5375	nuoC	1,803	4,236	471
JW5376	yfbT	651	2,769	849
JW5377	yfcE	555	3,661	1,323
JW5378	dedD	663	8,263	2,486
JW5380	trmC	2,007	1,148	114
JW5381	yfcM	549	2,966	1,080
JW5382	yfdI	1,332	204	31
JW5383	tfaS	303	2,696	1,785
JW5384	yfdL	519	4,211	1,618
JW5385	yfdN	495	7,938	3,203
JW5386	ypdJ	141	69	95
JW5387	torI	201	140	138
JW5388	ypdA	1,698	160	18
JW5389	ypdH	327	1,333	818
JW5390	insL	1,113	3	1
JW5391	yfeA	2,190	3,740	342

JW5394	ucpA	792	7,904	1,987
JW5395	yfeW	1,305	1,002	157
JW5396	ypfH	699	6,077	1,735
JW5397	hda	747	9,739	2,598
JW5399	yfgG	192	177	187
JW5400	yfgH	519	2,217	856
JW5401	guaB	1,467	3,165	434
JW5402	yfgJ	216	1,281	1,178
JW5403	der	1,473	5,441	739
JW5404	sseB	777	6,335	1,619
JW5405	yphG	3,282	2,362	144
JW5406	yphH	1,194	6,578	1,106
JW5407	yfhK	1,428	3,330	467
JW5408	yfhB	636	9,812	3,082
JW5409	yfiP	699	5,809	1,655
JW5412	yfiL	366	1,577	861
JW5413	rimM	549	1,689	618
JW5414	ffh	1,362	2,331	342
JW5415	yfjD	1,197	4,596	769
JW5416	recN	1,662	5,402	652
JW5418	yfjO	372	262	142
JW5419	yfjP	864	7,030	1,625
JW5420	ypjM	183	1,083	1,176
JW5421	ypjJ	201	266	264
JW5422	ypjA	4,581	1,638	72
JW5423	pinH	144	253	360
JW5424	ypjC	483	145	62
JW5425	ygaQ	333	470	283
JW5426	yqaC	597	539	182
JW5427	ygaT	978	769	157
JW5428	ygaY	882	6,330	1,434
JW5429	srlA	564	4,606	1,626
JW5430	srlE	960	2,215	459
JW5431	gutQ	966	14,557	3,015
JW5433	hypF	2,253	520	47
JW5434	ascG	1,014	11,128	2,195
JW5435	ascF	1,458	6,880	944
JW5437	rpoS	876	14,181	3,233
JW5438	ygbF	285	1,157	816
JW5440	ygcQ	861	4,548	1,051
JW5441	ygcR	780	5,250	1,341
JW5442	ygcU	1,455	2,330	320
JW5443	ygcW	786	796	202
JW5444	ygcE	1,479	5,767	778
JW5445	ygcG	873	0	0
JW5446	exo	846	6,318	1,500
JW5448	ygdI	228	394	339
JW5449	amiC	1,254	12,976	2,071
JW5450	ygdB	408	1,242	609
JW5451	ppdB	564	4,621	1,636
JW5453	yqeF	1,182	2,002	340
JW5454	yqeH	633	683	217
JW5455	yqeJ	483	502	205
JW5456	ygeI	219	100	92
JW5457	pbl	375	119	65
JW5458	ygeK	444	196	85
JW5459	ygeM	432	623	290
JW5460	ygeN	273	100	76
JW5461	ygeQ	795	1,693	424
JW5462	xdhA	2,259	7,389	656
JW5463	ygeW	1,191	2,996	504

JW5464	yqeC	771	4,149	1,078
JW5466	guaD	1,320	11,846	1,792
JW5467	ygfQ	1,368	3,908	571
JW5468	ygfS	489	721	296
JW5469	ygfT	1,920	1,733	180
JW5470	ygfU	1,449	4,673	643
JW5473	ygfB	579	1,132	387
JW5475	rpiA	660	15,371	4,640
JW5476	ygfI	897	2,482	557
JW5477	yggP	1,278	9,586	1,496
JW5478	tktA	1,992	18,107	1,818
JW5479	yggU	291	1,589	1,098
JW5481	mltC	1,080	9,007	1,656
JW5482	speC	2,136	5,113	482
JW5484	yghF	867	3,009	693
JW5486	glcF	1,224	2,781	454
JW5487	glcE	1,053	11,394	2,159
JW5490	yghQ	1,068	2,108	392
JW5491	yghS	714	2,749	773
JW5492	yghU	867	12,374	2,845
JW5493	hybF	342	3,695	2,150
JW5494	hybB	1,179	8,123	1,377
JW5496	yghY	354	2,196	1,240
JW5499	dkgA	828	1,063	259
JW5500	yqhG	927	3,814	823
JW5501	ygiQ	2,220	1,994	181
JW5502	ygiV	483	5,325	2,195
JW5503	tolC	1,482	937	126
JW5505	yqiC	291	762	523
JW5507	yqiG	2,466	4,432	361
JW5508	yqiH	750	970	260
JW5509	yqiI	1,065	2,888	544
JW5510	ygjG	1,407	8,718	1,236
JW5511	ebgA	3,093	3,665	237
JW5512	ygjI	1,434	3,099	432
JW5513	ygjO	1,137	4,213	741
JW5514	ygjP	504	4,799	1,901
JW5515	alx	966	7,766	1,601
JW5516	yqjC	369	906	488
JW5517	yhaL	165	0	0
JW5518	yhaM	1,311	9,514	1,453
JW5519	yhaO	1,269	2,648	420
JW5520	tdcG	1,365	6,713	979
JW5521	tdcF	390	0	0
JW5522	tdcE	2,295	3,087	268
JW5525	tdcR	219	11	10
JW5526	garR	891	3,894	877
JW5527	agaA	504	5,521	2,189
JW5528	yraR	636	4,116	1,293
JW5529	yhbO	519	873	339
JW5530	yhbV	879	4,782	1,087
JW5531	deaD	1,890	24,382	2,583
JW5533	yhbC	453	4,057	1,784
JW5534	yhbX	1,626	1,359	167
JW5535	yrbB	294	805	551
JW5536	arcB	2,337	645	54
JW5538	nanK	876	8,737	1,983
JW5539	yhcB	399	2,612	1,310
JW5540	yhcN	264	162	120
JW5541	aaeX	204	167	160
JW5542	yhdP	3,801	1	0

JW5543	yhdJ	885	247	56
JW5544	yhdX	1,182	969	163
JW5545	yhdY	1,104	3,510	639
JW5546	zraP	426	0	0
JW5547	nfi	672	4,936	1,465
JW5548	nudC	774	13,878	3,578
JW5549	thiG	771	7,490	1,951
JW5550	murI	858	5,406	1,257
JW5551	sthA	1,401	7,509	1,069
JW5553	argB	774	6,915	1,778
JW5555	ptsA	2,502	18,552	1,487
JW5556	gldA	1,104	21,726	3,938
JW5557	yijE	906	4,130	915
JW5558	cpxP	501	3,370	1,338
JW5559	yiiM	675	4,035	1,193
JW5560	kdgT	984	5,091	1,036
JW5561	rhaA	1,260	2,930	467
JW5562	frvB	1,452	3,785	523
JW5563	yiiF	219	28	26
JW5566	yihX	600	1,091	360
JW5567	yihW	786	4,421	1,125
JW5568	yihV	897	4,884	1,092
JW5569	yihS	1,242	9,473	1,525
JW5571	bipA	1,824	10,363	1,139
JW5574	yihF	1,431	965	135
JW5575	mobB	513	471	182
JW5576	trkH	1,452	12,206	1,684
JW5577	yigZ	615	11,703	3,785
JW5578	fadA	1,164	13,391	2,303
JW5580	tatB	516	4,157	1,610
JW5581	ubiE	756	12,294	3,243
JW5584	pldB	1,023	20,337	3,968
JW5585	rhtB	621	5,789	1,855
JW5586	rhtC	621	9,442	3,017
JW5588	yigI	468	9,494	4,043
JW5589	rarD	891	5,722	1,282
JW5590	yigG	381	161	84
JW5591	yigE	765	2,152	560
JW5592	dapF	825	6,974	1,685
JW5594	aslB	1,236	11,927	1,929
JW5595	yifK	1,386	22,926	3,295
JW5596	rffT	1,080	16,481	3,042
JW5597	rffC	546	6,602	2,409
JW5598	rffG	1,068	4,954	925
JW5599	rffD	1,263	18,248	2,901
JW5600	rffE	1,131	6,905	1,216
JW5601	wzzE	1,047	3,152	603
JW5603	gpp	1,485	8,835	1,192
JW5604	rep	2,022	3,764	375
JW5605	ilvD	1,851	2,868	313
JW5606	ilvE	930	10,396	2,241
JW5607	hdfR	840	3,584	850
JW5608	yieP	693	1,273	366
JW5609	trkD	1,869	13,903	1,483
JW5610	yieM	1,452	3,547	489
JW5611	atpI	381	1,415	737
JW5612	yieL	1,197	2,083	348
JW5613	yieK	723	3,936	1,087
JW5619	tnaB	447	2,568	1,145
JW5622	tnaB	984	1,855	376
JW5625	gyrB	2,415	6,195	512

JW5627	dgoR	690	4,121	1,185
JW5628	dgoA	618	12,624	4,069
JW5629	dgoD	1,149	9,943	1,730
JW5631	cbrA	1,065	5,911	1,110
JW5633	yidQ	333	5,982	3,581
JW5634	emrD	1,185	4,320	729
JW5636	yicO	1,335	1,090	164
JW5637	yicN	453	3,081	1,358
JW5641	ttk	597	7,189	2,408
JW5642	dfp	1,221	12,706	2,082
JW5643	yicR	669	1,078	322
JW5644	htrL	858	130	31
JW5645	yibQ	960	1,476	305
JW5646	envC	1,260	8,015	1,277
JW5647	yibJ	702	1,943	554
JW5648	yiaY	1,152	6,327	1,097
JW5650	sgbU	861	9,464	2,198
JW5651	yiaN	1,278	2,487	389
JW5652	avtA	1,254	3,600	572
JW5653	bax	825	5,662	1,372
JW5654	yiaB	342	543	321
JW5655	yiaF	711	5,626	1,585
JW5656	tiaE	975	3,312	677
JW5657	yiaD	660	5,542	1,672
JW5659	yhjY	699	5,190	1,483
JW5660	eptB	1,692	2,338	276
JW5663	bcsF	192	207	213
JW5665	bcsA	2,619	4,428	339
JW5668	kdgK	930	2,156	465
JW5669	hdeB	327	231	142
JW5670	yhiD	648	288	89
JW5672	yhiQ	753	5,509	1,460
JW5674	yhiK	393	580	295
JW5676	rbbA	2,736	8,002	586
JW5677	yhhJ	1,125	10,297	1,818
JW5678	yrhC	246	14	11
JW5679	rhsB	4,236	3,902	184
JW5680	yhhT	1,050	6,863	1,307
JW5682	dcrB	558	1,787	639
JW5683	yhhL	270	249	186
JW5686	gntU	1,341	7,852	1,167
JW5687	glpG	831	4,897	1,176
JW5688	rtcA	1,017	1,735	340
JW5689	malP	2,394	2,426	203
JW5690	gntT	1,317	2,426	368
JW5691	gntX	684	4,359	1,277
JW5692	hslO	879	9,790	2,217
JW5693	yrfD	780	4,368	1,119
JW5694	yrfA	405	1,146	563
JW5696	yhfY	363	3	1
JW5697	yhfU	354	4,425	2,500
JW5698	frlR	732	0	0
JW5699	frlC	831	10,956	2,631
JW5700	frlB	1,023	5,152	1,008
JW5701	yhfK	2,103	1,974	187
JW5702	crp	633	1,695	536
JW5703	yheO	723	749	209
JW5704	gspM	462	1,730	749
JW5705	gspL	1,164	2,370	405
JW5706	gspI	378	933	488
JW5707	gspD	1,953	5,537	565

JW5708	smf	1,125	9,273	1,643
JW5710	yrdA	555	1,430	511
JW5711	yjbF	639	3,517	1,096
JW5713	ubiC	498	1,919	769
JW5714	zur	516	6,449	2,488
JW5716	yjbO	243	298	247
JW5718	yjcB	282	364	256
JW5721	yjcS	1,986	1,834	184
JW5724	alsK	1,029	11,776	2,286
JW5727	phnK	759	11,365	2,978
JW5729	yjcZ	879	13,098	2,990
JW5730	eptA	1,644	18,641	2,270
JW5731	adiA	2,271	3,569	313
JW5732	yjdO	174	23	26
JW5733	yjdC	576	2,892	1,001
JW5734	dipZ	1,698	2,824	331
JW5735	dcuA	1,116	6,357	1,143
JW5736	yjeI	354	9,098	5,142
JW5737	ecnA	126	160	249
JW5738	sugE	318	3,552	2,222
JW5739	yjeM	1,503	1,741	230
JW5740	orn	546	4,169	1,525
JW5741	rnr	2,442	3,664	298
JW5742	yjfN	276	1,366	986
JW5743	yjfO	330	2,489	1,502
JW5744	ulaA	1,398	11,220	1,604
JW5745	ytfB	639	5,261	1,641
JW5746	fklB	621	5,283	1,698
JW5747	ytfH	381	760	400
JW5748	ytfI	939	689	146
JW5749	ytfK	207	153	148
JW5750	chpS	252	454	356
JW5752	ytfR	1,503	4,334	574
JW5753	ytfT	1,023	4,448	866
JW5754	yjfF	996	6,061	1,216
JW5755	yjgF	387	1,107	567
JW5756	yjgK	453	2,595	1,147
JW5757	yjgL	1,815	440	48
JW5758	yjgM	504	2,868	1,145
JW5759	yjgN	1,197	347	57
JW5760	yjgQ	1,083	1,122	204
JW5761	yjgB	1,020	18,745	3,672
JW5763	yjgX	243	309	257
JW5764	yjgX	360	2,515	1,394
JW5767	insG	1,329	0	0
JW5768	yjhB	1,218	462	75
JW5769	yjhC	1,119	131	24
JW5770	yjhD	231	273	233
JW5771	insN	267	0	0
JW5772	insM	369	0	0
JW5773	yjhW	243	26	22
JW5775	yjhH	906	932	206
JW5776	sgcX	1,122	7,652	1,363
JW5777	yjhT	1,107	1,622	294
JW5778	yjhA	717	2,118	587
JW5779	fimI	540	544	199
JW5780	fimD	2,637	1,441	109
JW5782	yjiD	393	2,492	1,267
JW5783	yjiH	684	5,547	1,625
JW5784	kptA	555	1,019	367
JW5785	yjiL	768	9,135	2,374

JW5786	yjiM	1,152	5,619	976
JW5787	yjiT	1,518	106	14
JW5789	mcrC	1,047	442	84
JW5790	yjiA	957	7,382	1,538
JW5791	yjiY	2,151	4,666	435
JW5792	yjjM	915	1,758	384
JW5793	yjjN	1,023	10,487	2,043
JW5794	mdoB	2,292	9,924	864
JW5795	yjjA	495	4,760	1,920
JW5796	yjjP	771	9,435	2,444
JW5797	yjjZ	237	213	189
JW5800	nadR	1,233	2	0
JW5801	yjjX	513	3,852	1,500
JW5802	ydbA	2,559	13,450	1,052
JW5803	ybhR	1,107	4,173	754
JW5804	ycjY	933	6,143	1,313
JW5805	hyfI	759	6,900	1,813
JW5806	tdcD	1,296	10,787	1,670
JW5807	leuB	1,092	6,878	1,258
JW5808	pcnB	1,398	15,099	2,158
JW5810	cdsA	858	6,855	1,599
JW5811	fhiA	1,713	3,992	465
JW5812	mbhA	756	9,611	2,535
JW5813	ykfC	1,146	3,023	528
JW5814	yaiF	387	1,164	593
JW5815	tfaD	564	818	291
JW5816	ybfE	294	237	160
JW5818	potG	1,134	11,881	2,092
JW5819	ybjS	1,014	6,847	1,349
JW5820	ycfS	963	11,754	2,442
JW5821	emtA	612	4,300	1,395
JW5822	abgT	1,527	2,758	361
JW5823	ydcH	225	154	137
JW5825	yneF	948	2,347	492
JW5826	asr	309	2,495	1,604
JW5827	ydhL	240	0	0
JW5829	infC	543	2,349	863
JW5830	yebN	567	5,552	1,947
JW5831	znuA	933	7,715	1,656
JW5832	yedQ	1,695	7,470	885
JW5833	yeeJ	7,104	1	0
JW5834	yeeY	930	6,808	1,459
JW5835	yefM	252	425	336
JW5836	cld	981	765	154
JW5837	yegR	318	51	34
JW5838	yohG	1,089	597	111
JW5839	yejO	2,511	2,598	208
JW5840	elaD	1,212	312	52
JW5841	prmB	933	5,578	1,196
JW5842	yphC	1,062	5,562	1,039
JW5843	norR	1,515	8,249	1,094
JW5844	ygcI	675	7,841	2,309
JW5845	ygcS	1,338	675	101
JW5846	ygeO	426	945	442
JW5847	prfB	1,099	396	72
JW5848	yghO	1,173	13,096	2,230
JW5849	yqhC	957	6,253	1,305
JW5850	yqjF	393	2,659	1,356
JW5851	pnp	2,205	2,929	264
JW5852	yihO	1,404	1,683	237
JW5853	ysgA	816	9,487	2,320

JW5854	yigL	801	4,027	1,008
JW5855	recQ	1,830	196	21
JW5856	trxA	330	2,656	1,593
JW5857	rbsD	420	1,049	502
JW5858	yidX	657	274	83
JW5859	dgoT	1,293	1,487	230
JW5860	yidR	1,215	12,135	1,999
JW5864	yrhA	414	242	117
JW5865	yrfG	669	2,824	848
JW5867	aidB	1,626	3,970	491
JW5868	ulaG	1,065	1,675	314
JW5869	yjiK	969	728	153
JW5871	mcrB	1,380	318	46
JW5873	prfC	1,590	3,099	388
JW5874	ydhM	516	8,211	3,184
JW5875	nuoB	663	4,880	1,475
JW5876	yfeH	999	17,397	3,468
JW5877	ypeB	219	267	243
JW5878	csiE	1,281	1,737	269
JW5879	mokC	210	22	20
JW5880	ylbG	372	0	0
JW5881	ychS	276	2	1
JW5882	mokB	168	67	79
JW5883	ychG	231	45	38
JW5884	lomR	156	0	0
JW5885	yoaI	105	0	0
JW5886	yegZ	219	19	17
JW5887	yfdT	306	16	10
JW5888	ypjK	237	0	0
JW5889	ygiA	261	1	1
JW5890	yjdP	330	0	0
JW5891	ytjA	180	29	31
JW5892	yadB	927	1	0
JW5893	yahH	297	0	0
JW5894	cynR	900	2,723	603
JW5895	insH	981	0	0
JW5896	ybhD	1,017	0	0
JW5897	yliA	1,839	0	0
JW5898	yccW	1,104	0	0
JW5899	insD	924	36	8
JW5900	insC	366	0	0
JW5901	ycgH	321	0	0
JW5902	insC	366	0	0
JW5903	insD	924	31	7
JW5904	lomR	171	0	0
JW5905	hrpA	3,846	0	0
JW5906	gapC	753	0	0
JW5907	yncB	1,038	0	0
JW5908	yddM	363	0	0
JW5909	ydfU	1,050	0	0
JW5910	ydiD	1,701	0	0
JW5911	yniD	123	0	0
JW5912	yedN	321	0	0
JW5913	intG	279	223	158
JW5914	insH	981	0	0
JW5915	molR	1,938	5,198	541
JW5916	molR	948	1	0
JW5917	rcsC	375	0	0
JW5918	insD	924	30	7
JW5919	insC	366	1	1
JW5920	rcsC	2,424	1	0

JW5921	yfjS	444	0	0
JW5922	ypjL	519	0	0
JW5923	ygfK	3,099	0	0
JW5924	yghE	861	97	22
JW5925	yghJ	4,563	0	0
JW5926	yghX	411	0	0
JW5927	ygiB	576	0	0
JW5928	insH	981	0	0
JW5929	yiiE	219	0	0
JW5930	yihA	597	107	35
JW5931	tatD	783	3,696	937
JW5932	hemC	942	0	0
JW5933	insD	924	26	6
JW5934	insC	366	0	0
JW5935	insH	981	0	0
JW5936	insH	981	0	0
JW5937	ysdC	177	0	0
JW5938	yicM	1,356	0	0
JW5939	yicJ	1,383	0	0
JW5940	bisC	2,280	0	0
JW5941	yhjQ	729	626	173
JW5942	bcsC	3,474	0	0
JW5943	yhjK	1,950	0	0
JW5944	yhiM	1,053	1,175	224
JW5945	yhhS	1,218	0	0
JW5946	gntR	996	0	0
JW5947	aroK	522	0	0
JW5948	yhfZ	906	0	0
JW5949	yrdD	543	0	0
JW5950	yjbN	1,038	0	0
JW5951	insH	981	0	0
JW5952	yjhU	987	0	0
JW5953	yjiP	312	0	0
JW5954	yjiV	2,721	0	0
JW5955	bglJ	624	0	0
JW5956	ykfH	222	800	719
JW5957	ldrA	108	20	38
JW5958	ldrB	108	1	2
JW5959	ldrC	108	10	19
JW5960	ymjC	183	21	23
JW5961	yncK	288	207	146
JW5962	sra	138	90	133
JW5963	blr	126	27	44
JW5964	ypaA	186	0	0
JW5965	yicS	294	230	157
JW5966	ldrD	108	156	295
JW5967	sgcB	279	386	278
JW5968	yjhX	258	546	418
JW5969	yliL	270	0	0
JW5970	yccV	318	0	0

Supplementary Table 2
Genes included in isolated liposomes

Fluorescent liposome 1		Fluorescent liposome 2		Fluorescent liposome 3		Fluorescent liposome 4		Fluorescent liposome 5		Fluorescent liposome 6		Fluorescent liposome 7		Fluorescent liposome 8		Fluorescent liposome 9		Fluorescent liposome 10	
ORF No.	ORF name	ORF No.	ORF name	ORF No.	ORF name	ORF No.	ORF name	ORF No.	ORF name	ORF No.	ORF name	ORF No.	ORF name	ORF No.	ORF name	ORF No.	ORF name	ORF No.	ORF name
JW0335	lacZ	JW0169	ispU	JW0223	yafO	JW0335	lacZ	JW0335	lacZ	JW0163	map	JW0258	ykgN	JW0335	lacZ	JW0067	tbpA	JW0335	lacZ
JW0957	hyaD	JW0335	lacZ	JW0335	lacZ	JW0629	mrdB	JW1430	ydcN	JW0335	lacZ	JW0335	lacZ	JW0579	fepE	JW0335	lacZ	JW0933	pqiA
JW1149	ycgE	JW0429	lon	JW2473	hyfH	JW0647	gltL	JW1941	yedI	JW1700	btuE	JW0695	ybfL	JW1279	rnb	JW0456	priC	JW2694	hycB
JW1669	sufE	JW0448	maa	JW3922	frwB	JW0649	gltJ	JW1980	yoeE	JW2770	fucO	JW0696	ybfD	JW1841	zwf	JW0846	artQ	JW3200	rplM
JW2185	ccmE	JW0874	lolA	JW3982	yjbC	JW2262	elaA	JW2096	yehB	JW3331	cysG	JW0766	moaC	JW2432	eutL	JW1461	narW	JW3209	aaeB
JW2214	atoC	JW1489	pqqL			JW2452	aegA	JW2439	eutE	JW3413	yhhA	JW1455	ydcC	JW2466	hyfA	JW1469	yddG	JW5962	sra
JW2347	yfdH	JW5966	ldrD			JW2703	mutS	JW2704	pphB	JW3930	argC	JW1941	yedI	JW2702	ygbA	JW1519	yneJ		
JW3568	yibA					JW2939	pppA	JW3096	garP	JW5797	yjjZ	JW2093	yohM	JW3155	sfsB	JW1818	htpX		
JW4204	pyrB					JW5924	yghE	JW3492	yhjG	JW5961	yncK	JW2339	yfcY	JW3597	rfaL	JW2077	gatB		
JW5130	yccU											JW3451	yhhI	JW4005	yjbJ	JW2086	yegU		
JW5184	cvrA											JW3877	rhaR	JW5645	yibQ	JW2542	yfhD		
JW5761	yjgB											JW3886	fieF						
												JW3889	cdh						
												JW3891	yiiQ						
												JW5124	ycbF						
												JW5559	yiiM						
												JW5832	yedQ						
												JW5873	prfC						

Non-fluorescent liposome 1		Non-fluorescent liposome 2		Non-fluorescent liposome 3		Non-fluorescent liposome 4		Non-fluorescent liposome 5		Non-fluorescent liposome 6		Non-fluorescent liposome 7		Non-fluorescent liposome 8		Non-fluorescent liposome 9		Non-fluorescent liposome 10	
ORF No.	ORF name	ORF No.	ORF name	ORF No.	ORF name	ORF No.	ORF name	ORF No.	ORF name	ORF No.	ORF name	ORF No.	ORF name	ORF No.	ORF name	ORF No.	ORF name	ORF No.	ORF name
JW1264	sohB	JW3422	livH	JW0642	ybeT	JW0436	cof	JW1442	ydcZ	JW0053	imp	JW0408	pgpA	JW0057	rluA	JW0144	hrpB	JW1253	trpB
JW5553	argB	JW3909	metJ	JW1987	yeeV	JW0564	cusA	JW1458	nhoA	JW0214	yafK	JW0410	dxs	JW0725	ybgE	JW0729	tolA	JW2367	evgS
JW5957	ldrA	JW4194	pmbA	JW2028	wcaM	JW1558	flxA	JW1571	intQ	JW1520	yneK	JW1496	ydeQ	JW1359	ynaK	JW2393	yfeC	JW2880	serA
JW5959	ldrC	JW4361	creB	JW2063	baeS	JW2535	glyA	JW3598	rfaK	JW2004	hisB	JW1546	ydfQ	JW1715	yniB	JW2972	yghA	JW3152	rpmA
		JW5622	tnaB	JW3201	yhcM	JW2942	yghK	JW3947	rplA	JW2041	wcaD	JW1587	ynfL	JW1983	yeeR	JW3067	yqjB	JW3820	fre
				JW3688	mdtL	JW4057	phnL	JW4066	phnD	JW2655	ygaX	JW1677	ydiJ	JW2192	napH	JW5516	yqjC	JW3827	hemG
				JW5178	ymgG	JW4276	fimE			JW3206	argR	JW1906	fliZ	JW3693	yieH			JW3996	lamB
										JW5590	yigG	JW2440	cchB	JW4233	yjgW			JW5049	ykiB
												JW2520	hcaT	JW5394	ucpA			JW5137	ycdG
												JW3612	rpmB	JW5438	ygbF			JW5173	icdC
												JW5189	ychM	JW5763	yjgX			JW5577	yigZ
												JW5757	yjgL	JW5846	ygeO			JW5931	tatD
